
JOURNAL INFORMATION
==============================
NLM Title Abbreviation: J Med Phys
NLM Journal ID: 9441104
Journal ID: JMP

Journal of Medical Physics / Association of Medical Physicists of India

ISSN: 0971-6203
EISSN: 1998-3913
Publisher: Wolters Kluwer -- Medknow Publications

ARTICLE INFORMATION
==============================
PMCID: PMC3146082
Article ID: JMP-31-116
Article version: 1
Subjects: Synopses of Papers Presented at Ampicon 2006

Synopses of Oral Papers

Print publication date: 2006 Jul-Sep
Volume: 31
Issue: 3
First page: 116
Last page: 153
Copyright: © Journal of Medical Physics
Copyright year: 2006
License: This is an open-access article distributed under the terms of the Creative Commons Attribution License, which permits unrestricted use, distribution, and reproduction in any medium, provided the original work is properly cited.
License URL: https://creativecommons.org/licenses/by/2.0/

Conference date: November 9-12, 2006
Conference name: 27th Annual Conference of Association of Medical Physicists of India
Conference Acronym: AMPICON-2006
Conference location: Hotel Mayfair Lagoon, Bhubaneswar
Conference sponsor: Hemalata Hospitals and Research Centre:

==============================
[Downloaded free from http://www.jmp.org.in on Friday, July 29, 2011, IP: 130.14.254.25] || Click here to download free Android application for t

International Conference on Medical Physics
27th Annual Conference of Association of Medical Physicists of India
(AMPICON-2006)
November 9-12, 2006, Hotel Mayfair Lagoon, Bhubaneswar,
(Host: Hemlata Hospitals and Research Centre)

### SYNOPSES OF ORAL PAPERS

### O-01
### A comparative study of three coplanar IMRT plans for fatty prostate patients by using pinnacle 3-D treatment planning system

Suvendu Sahoo, AK Rath, NK Painuly, BK Mohanta, H Mod*, S Pattnaik*. *Dept. of Medical Physics and *Radiation Oncology, Hemalata Hospitals and Research Center, Bhubaneswar, Orissa, India*

**Introduction:** For localized prostate cancer; radiation therapy is an effective modality. Prostate is one of the sites that is well suited for IMRT. IMRT planning and treatment delivery show significant potential for further improving the therapeutic ratio and reducing toxicity and thereby improving the quality of life. It is the responsibility of the medical physicist (planner) to obtain an optimal IMRT plan. The purpose of this study is to highlight some important points that need to be taken care of before embarking upon the IMRT plan. These are: (A) the treatment site (B) the facilities available with machine i.e., number of photon beams, MLC or mMLC and (C) the patient separation (thickness) from all sides. Of the above three points; first two points are well known to a planner. We want to highlight the third point i.e., the patient thickness, which is an important parameter when planning IMRT for prostate cancer. Our objective is to show by comparing three co-planner particular IMRT plans; the effect of numbers of beams from various directions on the final dose distribution.

**Materials and Methods:** Our centre is equipped with PINNACLE 3-D treatment planning system and digital linear accelerator (ELEKTA) having 40 pairs MLC facility, each MLC width is 1 cm at isocenter. For our study we took 5 patients of localized prostate cancer, whose AP-PA separations were 26 to 30 cm and lateral separations were 36 to 40 cm. For the above comparative study we generated three coplanar IMRT plans by using 6 MV photon beam. For the first and second plans we used 5 fields. The gantry angles were 35°, 100°, 180°, 270° and 325° and 0°, 75°, 135°, 225° and 285° respectively; with couch angle 0°. The third plan had 7 fields for which the gantry angles were 0°, 51°, 102°, 153°, 204°, 255° and 306° with couch angle 0°. ICRU 50 recommendation was followed for the contouring of CTV and other critical structures. The PTV was generated from CTV having 5 mm 3D-marigin. The critical structures taken were rectum, bladder and the femoral heads. Our study has compared three plans on the basis of dose volume histograms (DVH) of all parameters like PTV and other all critical structures.

**Results and Discussion:** The effect of number of beams in IMRT planning for fatty cases is an important decision for a planner. The mean data of DVH of 5 patients for 3 IMRT plans are given in the Tables. It was seen that, out of the three plans; the third plan was most suitable for patient treatment because the out side PTV maximum dose was much higher in first two plans and comparatively less in the third plan. It was seen that in the case of fatty patients the beam path length is more in patient body thereby leading to more deposition of dose in the patient body comparative to Planning Target Volume in 5 field plans. But in case of 7 fields plan, due to more number of beams the deposition of dose out side PTV was less thereby delivering the maximum dose to planning target volume.

**Conclusion:** This study states that, for fatty prostate patients the 7 fields IMRT plan is a better optimum plan as compared to a 5 fields IMRT plan. This conclusion will thus be helpful to a planner during a prostate IMRT planning.

Table 1: PTV dose comparison

| | PTV coverage | PTV min dose (Gy.) | PTV max dose (Gy.) | PTV mean dose (Gy.) |
|---|---|---|---|---|
| PLAN-1 (5 FIELDS) | 95% | 58.16 | 80.42 | 74.92 |
| PLAN-2 (5 FIELDS) | 95% | 67.05 | 79.48 | 75.57 |
| PLAN-3 (7 FIELDS) | 98% | 72.0 | 78.0 | 76.02 |

(Total planned dose 75.6 Gy. to PTV), PTV - Planning target volume

Table 2: Organ at risk volume dose comparison

| | Rectum dose (Gy.) | | | Bladder dose (Gy.) | | |
|---|---|---|---|---|---|---|
| | Max | Min | Mean | Max | Min | Mean |
| PLAN-1 (5 FIELDS) | 62.40 | 2.0 | 28.01 | 104 | 1.7 | 9.4 |
| PLAN-2 (5 FIELDS) | 60.1 | 1.9 | 19.75 | 77.85 | 1.0 | 7.40 |
| PLAN-3 (7 FIELDS) | 60.6 | 3.15 | 23.37 | 72.6 | 3.30 | 9.0 |

Table 3: Healthy tissue volume dose comparison

| | Dose out side PTV (Gy.) | | |
|---|---|---|---|
| | Max dose | Min dose | Mean dose |
| PLAN-1 (5 FIELDS) | 90.5 | – | 20.37 |
| PLAN-2 (5 FIELDS) | 85.6 | – | 21.2 |
| PLAN-3 (7 FIELDS) | 65.00 | 6.75 | 22.0 |

PTV - Planning target volume

### O-02
### Study of dose modeling for IMRT beamlets

Sudesh Deshpande, Suresh Chaudhari, V Anand, Sandeep De, V Kannan. *Department of Radiation Oncology, P. D. Hinduja National Hospital and Medical Research Center, Mahim, Mumbai, India*

**Introduction:** Intensity modulated radiation therapy (IMRT) beams are created by using a segmentation algorithm that converts the ideal fluence map into deliverable beam segments. This fluence can be delivered using step and shoot technique or dynamic sliding window technique. For accurate optimization, dose calculation and segmentation treatment planning system (TPS) need to be accurately modeled. AAPM TG report 53 and TRS 423 described different quality assurance tests for treatment planning system. All commercial treatment planning system passes these tests well within specified limit. Since these tests are designed basically for 3D-CRT most of the test are for square and rectangular fields. Many treatment planning systems require dosimetric data from field size 4*4 to 40*40 cm$^2$ shaped with secondary collimator. In case of single focus MLC IMRT delivery; secondary jaws are away from MLC leaf end therefore scattering, leakage and dosimetric property will change. It is important for treatment planning systems to compute the dose distributions created by small MLC beamlets and off axis from the center accurately. The purpose of this work is to perform quality assurance tests that simulate different segments of IMRT field. Dose measurements are done within field and outside fields and compared with treatment planning system.

**Materials and Methods:** To simulate IMRT beamlets seven static elongated fields of field size (1*20) were created in varis shaper terminal using MLC (40 pairs of I cm leaf width at Isocentre). The field shape

[Downloaded free from http://www.jmp.org.in on Friday, July 29, 2011, IP: 130.14.254.25] || Click here to download free Android application for t was positioned away from beam central axis in 1-cm steps. So that the final seventh field was centered 6 cm away from the central axis. Secondary jaws position were kept at 13.6*20.4 $cm^2$, which was a same, as recommended jaw position for IMRT field of 12*20. Percentage depth dose (PDD) was measured for all seven beamlets with 0.01cc-chamber and photon diode in RFA 300 (Scanditronix and Wellhofer Germany). All PDD curves were normalized at 1.5 cm. All seven MLC beamlets were used to measure dose profile parallel to direction of beam travel passing through center axis. Dose profile measurement was done at 5 cm using EDR2 film in solid phantom. Film was analyzed with Ominopro software. The LINAC used for this study has single focused MLC of 80 leaves (Varian 2300 CD). Only 6MV-photon beam was used in this study. In eclipse treatment planning system 30*30 $cm^2$ water phantom was created With same MLC shaped seven individual plan was created.With calculation grid of 2.5 mm calculation was performed. For dose profile study 1.25 mm grid was used for calculation. Calculated dose profiles and PDD in treatment planning system were compared with measured values. For comparison of dose profile 80-20% penumbra was used.

**Result and Discussion:** PDD measurement was done perpendicular to central axis. Because of divergent nature of radiation beam some part of PDD was measured outside radiation beam at higher depth. We found depth dose shapes accurately modeled by treatment planning system for depth greater than 2 cm in radiation field. Accuracy of treatment planning system in radiation field is well within 2% for all depths. But noticeable difference is found when depth dose measured outside radiation field at depth 15 cm and above. Measured depth dose curves shows bulging outside radiation field for higher depths. This bulging of curve increases as we move away from central axis. This bulging of curves could not model accurately by treatment planning system. Treatment planning system underestimate dose outside radiation area. The profile penumbra sharpens is accurately modeled by treatment planning system. Calculated value of 80-20% penumbra from planning system well agrees with measured values. Variation in depth dose curve outside radiation field needs further detail investigation

**O-03**

**Fluence map verification of step and shoot IMRT fields using amorphous - Silicon EPID**

Raghavendra Holla, TR Vevek, Preeti Deka. *Manipal Hospital, Bangalore, India*

**Introduction:** Amorphous - Silicon flat panel detectors are currently used to acquire portal images with good image quality for patient alignment before external beam radiation therapy. Although originally designed for imaging, the ability of the EPID to acquire rapidly a large two-dimensional array of digitized X-ray data is extremely attractive for dosimetry measurement as well. IMRT QA process can be tedious and the film dosimetry verification on intensity map is very time consuming. The aim of this study is to test the feasibility of using an EPID system to independently measure the beam fluence during IMRT delivery.

**Materials and Methods:** An Elekta Precise Digital accelerator with I View GT Amorphous - Silicon flat panel imaging system along with Scanditronix Omnipro IMRT verification software was used in this study. Once the IMRT plan is acceptable clinically, the same plan is exposed over a flat surface phantom. All the beam angles and collimator angles are set to zero for simplicity. To study the impact of beam attenuation as encountered in clinical practice, EPID images were acquired with homogenous solid phantom of 5 cm thickness placed in the beam. In normal operation, the I View GT EPID continuously acquires images as a series of frames of fixed time (around 0.320 sec). At the end of the acquisition final 16 bit image is created by averaging all the frames, then normalizing to the highest gray scale region in the image thus discarding all dosimetric data. To prevent this the number of frames were recorded for each segment image. Each segment image is recorded separately, which carries individual renormalization factor called pixel factor. All the segment images are imported to Omnipro IMRT Software with original pixel factor. Composing all the segment images at Omnipro IMRT system created an image proportional to dose. Renormalized the composed image at central axis to 100%. The Adac Pinnacle planning system used for IMRT planning calculates planar dose at a predefined source to plane distance. Planar dose at 5 cm depth for the corresponding field was imported to the software from the planning system. Each field was evaluated by comparing two dimensional reconstructed fluence map from EPID with planar dose map from Pinnacle system. For this purpose, the gamma evaluation method was used with a dose difference criterion of 2% of dose maximum and a distance to agreement criterion of 2 mm. Results and conclusion: The results obtained with EPID have been compared with the fluence distribution obtained with film. Relative to the film, the results are comparable and are within 3%. Excellent agreement was found between TPS and EPID measurements with 95% of pixel counts falling with in the pass criteria. Amorphous - Silicon EPID offers time saving method for checking beam fluence for step and shoot IMRT delivery. Though the absolute dosimetry information of the intensity map was not generated during the normalization process, the Gamma evaluation with TPS fluence can provide a efficient way to verify intensity maps in IMRT. In addition to the fluence map verification, other checks like profile at low monitor units, leaf positioning of MLC leafs can also be performed using EPID.

**O-04**

**Evaluation of aperture based inverse planning for intensity modulated radiotherapy**

Tushar Bopche, Kalpana Thakur, Ruchita Shah, Satish Pelagade, RK Vyas. *Department of Medical Physics and Radiotherapy, Gujarat Cancer Research Institute, Ahmedabad, India*

**Introduction:** To study the clinical efficiency of aperture based inverse planning for intensity modulated radiotherapy by evaluating dose volume histograms (DVH).

**Materials and Methods:** Aperture based inverse planning method uses a predefined set of apertures. The apertures or segments can be designed by giving different margins to include target volume and to exclude critical structures. Some rules are used to establish aperture shapes. First a conformal segment that encompasses the entire target including critical organs if any coming in field is defined. Second, a field segment that conforms to the target but stops short of including the critical organs is designed. Third a field segment that covers the remaining portion of target without including the critical organ is established. There can be as many apertures as one can design with several combinations of the margins to target and normal tissue. The number of such apertures is typically smaller than 100 for even the most complex cases. Based on dose constraints for targets and critical structures, weights are determined for these apertures only. The dose volume optimisation may discard many of the apertures which are not significant for desired dose distribution and end up with an even smaller final number of apertures. At last we can delete low MU segments and those segments which are having very small area. The lower limitation on MU and field size is dependent on treatment machine parameters. Finally plans are analyzed in 2D and 3D also with DVH.

**Results and Discussion:** The DVH analysis showed good target coverage and significant normal tissue sparing. The aperture based inverse planning significantly reduces both the number of segments and monitor units used for IMRT. This is accomplished without loss of dose coverage to for the targets and sparing of nearby critical structures. The large decrease in number of apertures reduces wear and tear of MLC system and lower monitor units results in reduced total body dose of the patient also.

**O-05**

**Evaluation of pixel ionization chamber for IMRT planner dose verification**

G Arun, M Dinesh Kumar, K Ramalingam, M Babaiah. *Department of Radiotherapy, Yashoda Cancer Institute, Secunderabad - 500 003, AP, India*

**Introduction:** A recent advance in external beam radiation therapy

This PDF is available for free download from a site hosted by Medknow Publications (www.medknow.com).

[Downloaded free from http://www.jmp.org.in on Friday, July 29, 2011, IP: 130.14.254.25] || Click here to download free Android application for t is the use of non-uniform intensity photon fields to produce dose distribution conformed to complex targets. Due to the complexity of the IMRT, the verification of dose delivery is decisive and clinical implementation of IMRT requires verifying the consistency between calculated and delivered dose distribution. In general, IMRT QA is broadly divided into machine and plan (patient) specific. Lengthy and tiresome procedure in plan (patient) specific IMRT QA is planar dose verification. Conventionally we do this with Film dosimetry. But the time spent to collect data with the film is quite long and influence of uncertainty is high in film processing. In the present work, the performance of Pixel Ionization Chamber array has been evaluated for imrt fluence verification.

**Materials and Methods:** A pixel ionization chamber (PIC) array (I'mRT MatriXX) from Scanditronix has been used for this study. The detector features a 24.4x24.4 cm$^2$ active area divided in 1020 independent vented plane-parallel ionization chambers and can read out with a minimum sampling time of 20 ms per 2D field without introducing dead time. Each chamber has 4.5 mm diameter and 5 mm height; a distance of 7.62 mm separates the centre of adjacent chambers. The sensitive volume of each single ionization chamber is 0.07 cm$^3$. For this study, CT images of IMRT matrix and perspex phantom were acquired and test patterns of X-wedges, Y-wedges, Dose well and two clinical IMRT fluency maps were imported into these phantoms as a separate plan in the Eclipse Planning system. All plans were normalized at isocentre at a depth of 2.5 cm from the surface. Measurements were performed in dMLC mode, on a Varian Clinac DMX delivering a 6 MV photon beam, equipped with Varian Millennium 52 leaf MLC. Delivery setups for all measurement were analogous to planning setups and ambient corrections have been applied for PIC. Beam profiles for different field dimensions and dose distribution for IMRT test patterns were measured with the PIC and Kodak EDR2 film. Films were scanned by VIDAR, VXR-16 scanner. The analysis of measured (Film, PIC) versus calculated (TPS) absorbed dose distributions have been evaluated using gamma evaluation method in Omni-Pro IMRT software from Scanditronix.

**Result and Discussion:** It was found from all measurements that the agreement between the PIC measurements and treatment planning calculations were within the experimental uncertainties and it accepted 3% Dose Difference (DD) and 3 mm Distance to Agreement (DTA) criteria. Results obtained from the film agreed 3% DD and 3mm DTA criteria with the PIC measurements. While comparing the film distribution with PIC and TPS dose distribution in the high dose gradient and penumbral region, small discrepancies were found, because of its low resolution.

**Conclusion:** In summary, for fast, reliable and real time planar dose verification, the pixel ionization chamber array (IMRT matrix) can be useful.

### O-06
### Evaluation of stereotactic field detector for small field dosimetry

N. Karthikeyan, M Dinesh Kumar, G Arun, K Ramalingam, M Babaiah. *Department of Radiotherapy, Yashoda Cancer Institute, Secunderabad - 500 003, AP, India*
E-mail: arunharisri@yahoo.com

**Introduction:** The modern advances in Linear accelerators and treatment planning computing now allow for small, tumor-shaped fields to be used in everyday radiotherapy. Recent Treatment planning systems require accurate modeling of the beam that is specific to each treatment machine and beam energy and then used for accurate dose calculations. Those data have to be measure systematically in terms of relative scatter factor, percent depth dose and beam profile with suitable detector. In the present work, dosimetrical characteristics of small field have been investigated with different types of detectors.

**Materials and Methods:** The measurements were carried out on the Varian Clinac DMX dual energy [6MV and 16MV] linear accelerator, equipped with Millennium 52 leaf MLC which creates a leaf width of 1 cm at isocentre. A Micro Multi Leaf Collimator (mMLC) from Brain Lab can be attached separately with the Linac for SRS and SRT. Small field sizes were created with the mMLC having the leaf widths projected to the isocentre are 3 mm, 4.5 mm and 5.5 mm for the inner (14), middle (6) and outer (6) leaves, respectively and the photon energy of 6MV is used for all measurements. For this study, Relative scatter data, Percentage depth dose and Beam profiles have been measured and investigated by using Stereotactic Field Detector (SFD), farmer type ion chamber FC65G (0.6cc), mini-ion chamber CC13 (0.13cc), micro chamber A14 (0.01cc), RK chamber and conventional Kodak EDR2 films. For scatter factor measurement, test files were created using the Varian Shaper Program and data were acquired by using 0.6cc, 0.13cc, 0.01cc chambers, Stereotactic Field Detector SFD and Film. The scatter factors were measured for constant monitoring units (100 MU) at the depth of 5 cm and SSD kept at 100 cm for a matrix of combinations of square mMLC fields Times Square jaw settings. The normalized measured scatter factors were compared with Monte Carlo values. Computer controlled Radiation Field Analyzer (RFA300) and SFD have been used to acquire percentage depth dose data. The mMLC field size has been kept always equal to collimator jaw setting and the PDDs for the mMLC square field sizes of 6x6, 12x12, 18x18, 24x24, 30x30, 36x36, 42x42, 60x60, 80x80, 100x100 [mm$^2$] were measured. The beam profiles have been acquired by using SFD, RK Chamber and Film for mMLC field size 6 x 6 cm$^2$ at the depth of 5 cm and SSD kept at 100 cm. PMMA Phantom has been used for film experiments. Penumbra has been analyzed from SFD and film for the mMLC square field sizes 6x6, 12x12, 18x18, 48x48, 60x60, 10x10 (mm$^2$).

**Result and Discussion:** In relative scatter factor measurements, the results shows that SFD value is much closer to the reference value and high deviation were found for ion chamber measurement due to partial irradiation of the active volume and results in the lack of lateral electronic equilibrium inside the volume in small field sizes. Though the volume of the micro chamber is very low, it gives the underestimation of dose because of low signal collection in small fields. The beam profile results shows that profiles obtained by SFD, RK chamber and film are almost same. Film gives lesser penumbra and it indicates that the beam penumbra increase as the diameter of mMLC field size increases.

**Conclusion:** In general, a dedicated Stereotactic Field Detector provides more reliable and accurate beam data and it can be used for small field dosimetry.

### O-07
### Entrance dose calibration for MOSFET detectors in 6MV and 15MV photon beams

G Bharanidharan, D Manigandan, K Devan, D Elangovan*, R Tamilarasan*, A Vasanthan*, P Aruna, S Ganesan. *Division of Medical Physics and Lasers, Department of Physics, Anna University, Chennai-600 025, Tamil Nadu, *Department of Radiation Oncology, Kailash Cancer Hospital and Research Center, Muniseva ashram, Goraj - 391760, Ta. Vagodhia Dist. Vadodara, Gujarat, India*

**Introduction:** *In vivo* patient dose verification is considered to be an important part of quality assurance in radiotherapy. The discrepancies between the planned dose and the delivered dose can also be verified usinginvivo dosimetry. The commonlyused detectors in *in vivo* dosimetryare silicon diode detectors and thermoluminescent dosimeters. Entrance *in vivo* dosimetry is done for not only to detect dosimetric errors such as human errors in data generation and data transfer, but also those due to equipment breakdown or malfunctioning. In general, entrance dose measurements serve to check the output and performance of the treatment device, the accuracy of patient setup and the calculation of the number of monitor units. In this context, semiconductor detectors seemed suitable for entrance *in vivo* dose measurements. Generally such measurements are carried out by placing the detector on the patients skin and the detectors should be covered with proper build-up cap, This may confirms that the detector is measured under electronic equilibrium conditions. In the case of insufficient build-up cap large correction factors are required. This may influence the accuracy of the *in vivo* measurements. However, MOSFET detectors are supplied without build up caps. By considering these, the current study is aimed to calibrate the MOSFET

This PDF is available for free download from a site hosted by Medknow Publications (www.medknow.com).

[Downloaded free from http://www.jmp.org.in on Friday, July 29, 2011, IP: 130.14.254.25] || Click here to download free Android application for t for *in vivo* entrance dose measurements with two different build up cap materials.

**Materials and Methods:** The MOSFET dosimeter used is the commercially available dosimetric system of model TN502RD standard isotropic product (Thomson and Neilson, Ottawa, Canada). It consists of a MOSFET detector with 20 cm-long × 2.5 mm-wide × 0.4 mm-thick flexible cable, a 9V Dual bias supply box (TN-RD-22), a reader with a liquid crystal display (TN-RD-10) and Bias reader cable (TN-RD-21). The build up cap used was a wide energy hemispherical Brass buildup cap of 0.635 radius (TN-RD-56-0.63) for both 6MV and 15MV. This build up material can be used in full photon range and 15MeV to 18MeV electron beams as per the manufacturer specification. Bolus material is also used for measurements and the reading is compared with the brass build up cap. For 6 MV beam, 1.5 cm water equivalent bolus material was used and for 15MV beam 3 cm bolus was used. The phantom used was cylindrical PMMA phantom of 30x30 $cm^2$ with a density of 1.19 g $cm^{-3}$.

**Results and Discussion:** Detectors used for *in vivo* Dosimetry are calibrated by measuring their response when placed on the surface of the phantom at the beam axis. Entrance dose calibration is done usually by calculating the calibration factor at reference conditions i.e., $F_{cal.}$ the reference conditions were set as a 10x10 $cm^2$ field size at the isocentre and SSD equal to 100 cm for each correction factor one parameter is changed.

Entrance *in vivo* dose were computed by using the relation

$D_{ent} = R_{ent}\, F_{cal}\, P_I\, CF_i,$

where, $D_{ent}$ is the entrance dose, $R_{ent}$ is the reading from the *in vivo* detector, $F_{cal}$ is the entrance dose calibration factor in reference conditions and $CF_i$ is the various correction factors. In the current study, correction factor was done for various Field size, SSD and Physical wedge, using brass buildup cap and bolus material for 6MV and 15MV photon beams. The intrinsic precision for MOSFET with brass build up cap in 6MV and 15MV photon beam shows a standard deviation of ± 0.83% and ± 2.5%. The intrinsic precision of MOSFET covered with water equivalent bolus material in 6MV beam was ±1.7% and for 15MV beam it was ± 2.6%. The linearity of the detector for both 6MV and 15MV with both brass build up cap and bolus has a $r^2$ value of 0.9999. The standard deviation for field size correction factor between MOSFET covered with brass build up cap and bolus for 6MV and 15MV photon beam was less than ±1%. However, the standard deviation for MOSFET covered by brass build up cap was higher than the standard deviation for MOSFET covered by bolus for both 6MV and 15MV beam. The standard deviation for SSD correction factor between 6MV and 15MV for MOSFET covered with brass build up and bolus was less than ±1%. The standard deviation of wedge correction factor for MOSFET covered with brass build up cap and bolus for the wedge angle 15°, 30°, 45° and 60° for both 6MV and 15MV was less than 0.1%, because the electron contamination did not play an important role in this case.

### References

1. Jornet N, Carrasco P, Jurado D, Ruiz A, Eudaldo T, Ribas M, Comparison study of MOSFET detectors and diodes for entrance in vivo dosimetry in 18MV X-ray beams. Med Phys 2004;31: 2534-42.
2. Bharanidharan G, Manigandan D, Devan K, Subramani V, Gopishankar N, Ganesh T, *et al.* Characterization of responses and comparison of calibration factor for commercial MOSFET detectors. Med Dosim 2005;30:213-8.
3. Zhu XR. Entrance dose measurements for *in vivo* dosimetry: Comparison of correction factors for two types of commercial silicon diode detectors. J Appl Clin Med Phys 2000;1:100-7.
4. Kai Huang, William S. Bice, Oscar Hidalgo-Salvatierra Characterization of an *invivo* diode dosimetry system for clinical use. J Appl Clin Med Phys 2003;4:132-42.
5. Chuang CF, Verhey LJ, Ping Xia, Investigation of the use of MOSFET for clinical IMRT dosimetric verification. Med Phys 2002;29: 1109-15.

### O-08-gopiraj

### *Invivo* dosimetry using TLD during pelvic treatment

A. Gopiraj, AK Mahant*, S Vinatha*, V Ramasubramanian#, M Rekha.
*M. S. Ramaiah Medical College and Hosptial, Bangalore, *Radiation Safety Systems Division, BARC, Mumbai, #Vellore Institute of Technology, Dept. of Physics, Vellore, India*

**Introduction:** The chain of steps in specifying the dose delivered to a patient undergoing radiotherapy includes several steps that may introduce an uncertainty in the actual dose value. In clinical practice however this desired level of accuracy cannot always be achieved, due to several sources of uncertainty. An overall check of the whole dosimetry procedure by *in vivo* dosimetry is therefore useful and sometimes even necessary if a high accuracy is required. Accurate *in vivo* dosimetry can be performed with entrance and exit dose measurements. Any discrepancy between the measured and expected entrance dose can be caused by errors in patient set up in the number of monitor units or in the irradiation time, beam out put variation etc. The measured exit doses also provide interesting information about tissue thickness and tissue inhomogeneities through which the beam passes. Using combined entrance and exit dose measurements, one can measure the modification of the overall transmission caused by the presence of bone, air or soft tissue, as compared to the values calculated assuming the patient to be water equivalent. The purpose of this study is to derive and compare the mid plane dose from the measured entrance and exit doses for the patients undergoing pelvic radiotherapy treatment.

**Materials and Methods:** The external irradiation technique for the tumors in the pelvic region consists of a combination of an anterior-posterior (AP) and posterior-anterior (PA) open beam (two field SSD/SAD technique). For the two-field technique the dose is prescribed at the point positioned on the central axis of the beams in the midplane of the patient. Patient treatments have been performed with Cobalt 60 gamma rays generated by an ATC - C9 telecobalt unit. For each photon beam TLD using appropriate build up material were positioned on the entrance and exit side of the patient during the treatment. In this study the entrance and exit dose of each field for five patients was determined. The irradiated TLD's were sent to BARC for dose measurements. The reading of the TLD has been converted to dose values at the entrance and exit points. The measured entrance dose is usually compared to the expected entrance and exit dose as predicted by the treatment planning system. From the measured entrance and exit dose the midplane dose is calculated using arithmetic mean of entrance and exit dose, both corrected for inverse square law. The mid plane dose $D_c$ is given by

$2\,D_c = [D_0 * (f+d-m)^{\wedge}2 * 1/(f+d/2)^{\wedge}2] + [D_{ex} * (f+m)^{\wedge}2 * 1/(f+d/2)^{\wedge}2]$

Where

$D_c$ is the mid plane dose.

| *Patient ID* | *Technique* | *Measured entrance dose (cGy)* | *Measured exit dose (cGy)* | *Calculated midpoint dose (cGy)* | *Delivered midpoint dose from one field (cGy)* | *% variation* |
|---|---|---|---|---|---|---|
| 1. | SSD | 161.86 | 49.19 | 94.367 | 90 | 4.84 |
| 2. | SSD | 148.68 | 49.63 | 91.011 | 90 | 1.12 |
| 3. | SSD | 153.77 | 45.39 | 89.68 | 90 | -0.346 |
| 4. | SSD | 171.28 | 53.71 | 102.295 | 100 | 2.29 |
| 5. | SAD | 218.40 | 45.67 | 89.2087 | 90 | -0.87 |

This PDF is available for free download from a site hosted by Medknow Publications (www.medknow.com)

[Downloaded free from http://www.jmp.org.in on Friday, July 29, 2011, IP: 130.14.254.25] || Click here to download free Android application for t

$D_0$ is the measured entrance dose
$D_{ex}$ is the measured exit dose
F is the source to surface distance
d is the AP - PA separation of the patient.

The Table shows the measured entrance dose, measured exit dose, the calculated midpoint dose and the % variation of the dose for the 5 patients.

**Results and Conclusion:** In this study the accuracy and precision of in vivo dose determinations using TLD measuring system have been explored for patients treated in the pelvic region. The dose comparison performed in this study shows that for the simple irradiation geometry of the pelvic region, a deviations of +4.84% to -0.87% have been observed between dose valued determined at identical points in patients and calculated by the computer planning system. The check of the dose delivered to a given patient can be performed only by Invivo dosimetry. Large errors can be detected and the sources of error can be identified and corrected decreasing the uncertainty on the total dose delivered to each individual patient. Invivo dosimetry has been proved to be very useful as a part of departmental quality assurance program.

### O-09

### A study on ionization chamber volume influence on IMRT absolute dosimetry

K. Ramalingam, M Muthamizhselvan, M Dinesh Kumar, G Arun, M Babaiah. *Yashoda Cancer Institute, Secunderabad, A.P, India*

**Introduction:** IMRT is an advanced form of 3D conformal radiotherapy combining several intensity modulated beams to provide tumor dose homogeneity and critical organ tolerance. The complex nature of IMRT delivery imposes the need of a precise plan specific QA. In plan specific QA basically we do point dose measurements and 2D fluence verification. In general, for point dose measurements we ensure that the chamber dimension is less than field size. But in the case of non uniform IMRT fields, each pixel has different fluencies. The objective of present study is to assess the best suited chamber and ionization chamber volume effect for IMRT absolute dosimetry.

**Materials and Methods:** All measurements were carried out with our Varian CLINAC DMX C/D dual energy [6MV and 15MV] LINAC equipped with millennium 26 pair DMLC. In this study, three cylindrical ionization chambers FC65G (0.6cc), CC13 (0.13cc) and A14 (0.01cc) are used. Prior to non-uniform field dosimetry, the output factors for field sizes from 1x1 $cm^2$ to 10x10 $cm^2$ were measured in SP100 water phantom with these three chambers and compared. For this study, three pre-defined fluences like x-wedges with each strip width of 2 cm, 1 cm and 0.5 cm and also two fluences from clinical IMRT plans were imported to the ECLIPSE (3D/IMRT) treatment planning system as separate plans into a water phantom created. All the plans were normalized to deliver a dose of 100cGy at the isocenter at a depth of 5 cm in the water phantom. The planned IMRT fields were exported to the treatment unit through VARIS network and the plans were implemented on to the SP100 30x30x30 $cm^3$ water phantom and absolute doses at isocenter and ±2 cm lateral points were measured using the three ionization chambers and compared with ECLIPSE TPS calculated doses.

**Results and Discussion:** In the output measurements, larger variations are found at smaller field sizes for FC65G 0.6cc farmer type chamber compared to the other two small volume chambers. The results indicate that the chamber dimension must be smaller that the field size. In case of IMRT field absolute dosimetry, dosimetric errors are found in all the three chamber measurements. For 2 cm x-wedge fluence, CC13 showed less dose deviation compared to that of FC65G and A14 chamber. For 1 cm x-wedge and 0.5 cm X-wedge fluences FC65G chamber showed less deviation compared to that of CC13 and A14 chambers. Chamber A14 showed less variation only in few measurements. Our results infer that the influence of the chamber volume effect in IMRT dosimetry was not appreciable. In small volume chambers the dose variations found may be due to positional errors and chamber sensitivity. In case of large volume chambers variations are due to dose gradient within the chamber volume. So, in IMRT absolute dosimetry dosimetric errors can be managed by assuring that the dose measurement points are in regions of homogeneous integrated dose larger that the ionization chamber dimension.

**Conclusion:** Dosimetric errors in IMRT point dose measurements can be minimized if the cylindrical ionization chambers are placed in regions of homogeneous dose which is larger than the chamber dimension.

### O-10

### Use of modulation index for comparison of dynamic and step and shoot IMRT treatment techniques: A comparitive analysis

Anup Bhardwaj, TS Kehwar, SK Chakravarti*, AS Oinam, SC Sharma. *Department of Radiotherapy, Post Graduate Institute of Medical Education and Research, Chandigarh, *Department of Applied Physics, National Institute of Technology, Kurukshetra, India*

**Introduction:** Comparative analysis of two techniques, Dynamic and Step and Shoot for Intensity Modulated Radiotherapy Treatments using Modulation Index for head and neck cancer patients. Intensity Modulated Radiotherapy (IMRT) has become the treatment of choice for the cancer patients with tumor dose escalation and simultaneously sparing the surrounding normal critical structures which is not possible by 3 Dimensional Conformal Radiotherapy (3D-CRT). Now a days mainly two types of IMRT techniques are in clinical use; dynamic (sliding window) and step and shoot (segmental) with the help of multileaf collimator. One important question arising is, which is optimal technique between two taking into account the conformity to Planning target volume (PTV) and degree of complexity involved in delivery? Sliding window seems to be superior to segmental IMRT, but it requires more quality assurance checks because of complex nature of treatment delivery. Lesser complexity is involved with step and shoot delivery technique but PTV coverage seems to be inferior. Practically it is important to keep delivery technique as simple as possible but the increase in complexity should be justified with improvement in dose distribution. Modulation Index (MI) criterion given by S Webb can be used to compare the complexity involved in beam delivery in treatment plan.

**Material and Methods:** Patients having carcinoma of head and neck treated with Simultaneous Integrated Boost (SIB) IMRT with radiation dose 60 Gy and 54 Gy in 30 fractions to PTV1 and PTV2 respectively was selected for this study. Beam data of Varian high energy linear accelerator CLINAC DHX (2300 CD) having 6 MV and 15 MV photons, equipped with Millennium 80 multileaf collimator (MLC) was used. Millennium 80 MLC system has 40 leaf pairs having leaf width 1 cm at isocenter. Eclipse treatment planning system with Helios (Varian Medical System) inverse planning IMRT software was use to create IMRT plans for dynamic and segmental techniques. The optimal fluencies were converted to actual fluencies for sliding widow and by taking 5, 10 and

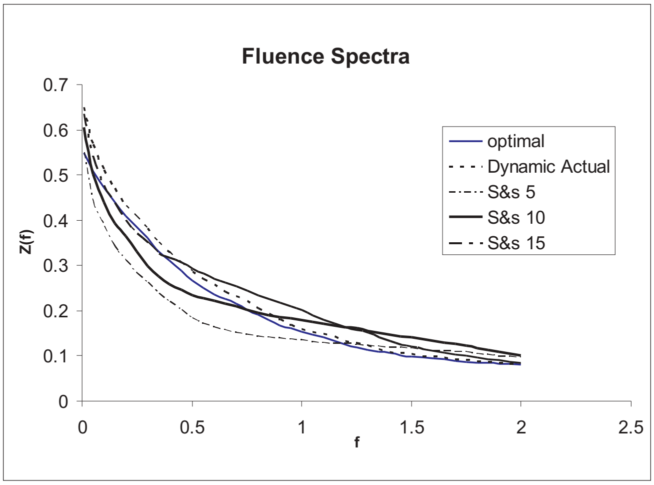

**Mean beam spectra for optimal and actual (dynamic, S & S 5, S & S 10, S & S 15) fluences**

This PDF is available for free download from a site hosted by Medknow Publications (www.medknow.com)

[Downloaded free from http://www.jmp.org.in on Friday, July 29, 2011, IP: 130.14.254.25] || Click here to download free Android application for t

Table: Average modulation indices of different IMRT techniques for different F values

| Technique | Upper integration limit F | | | | |
|---|---|---|---|---|---|
| | 0.25 | 0.50 | 1.00 | 1.50 | 2.00 |
| Optimal | 2.76356 | 4.319582 | 6.30303 | 7.498472 | 8.375477 |
| Dynamic | 3.03616 | 4.687548 | 6.819837 | 8.087981 | 8.989941 |
| S&S 15 | 2.860071 | 4.453272 | 6.851795 | 8.383626 | 9.371912 |
| S&S 10 | 2.636364 | 3.950216 | 5.957347 | 7.534632 | 8.730965 |
| S&S 5 | 2.312834 | 3.415712 | 4.901579 | 6.138019 | 7.197097 |

15 intensity levels for segmental technique. Modulation Index was computed for both techniques by using optimal and actual fluences taken from Eclipse planning system by making a program in Microsoft Excel. Degree of modulation was computed (Webb S.) by considering one directional intensity modulated beam. The spectrum was given by $Z(f) = N(f: \Delta p > f\sigma)/(n-1)$

where $\Delta p = abs(I_p - I_{p-I})$, $I_p$ is intensity of pth bixel, n is number of bixels in one dimensional beam, $\sigma$ is SD of intensity values, N is the number of changes between two adjacent bixels along leaf motion for which $\Delta p > f\sigma$ and f = 0.01, 0.02, 0.03,………, 2. Z(f) is fraction of changes among adjacent bixels that exceed a certain fraction (f) of the SD. MI is the area under the spectrum between zero and F.

$MI(F) = \int_0^F Z(f)df$

**Results and Discussions:** The global mean spectra for optimal and four actual fluences are shown in graph. From the graph it is seen that dynamic spectra is higher than optimal and S and S 15 spectra overlaps optimal spectra upto f < 0.4. Spectra of S and S 10 and S and S 5 are lower than optimal for f < 0.75 and 1.25 respectively. This shows the inadequacy of S and S 10 and S and S 5 to describe low gradient regions i.e. presence of high steps with S and S and not required by optimal fluence, leading to poor optimal actual coherence. However the results of S and S 15 and dynamic technique are almost similar.

**Conclusion:** For highly modulated fluence maps the dynamic technique should be preferred when emphasis is on target coverage and dose uniformity.

### References

1. Webb S. Use of a quantitative index of beam modulation to characterize dose conformality: Illustration by a comparison of full beamlet IMRT, few-segment IMRT and conformal unmodulated radiotherapy. Phys Med Biol 2003;48:2051-62.
2. Chui C, Chan M, Yorke E, Spirou S, Ling C. Delivery of intensity-modulated radiation therapy with a conventional multileaf collimator: Comparison of dynamic and segmental methods. Med Phys 2001;28:2441-9.

### O-11

### Monte carlo study of mosfet dosimeter for microbeam radiation therapy

NK Painuly, Anatoly Rozenfeld, George Takacs, Iwan M Cornellius, E Brauer-Krish*, A Bravin*. *University of Wollongong, New South Wales, Australia and Hemalata Hospitals and Research Centre, Bhubaneswar, India, *Eurepeon Syncrotron Radiation Facility, Grenoble, France*

**Introduction:** Microbeam Radiation Therapy (MRT) is one of the frontier areas of research and an emerging radiation oncology modality, which utilizes highly collimated, polarized, extremely intense and tuneable synchrotron radiation beams over a wide range of energies for the treatment of brain cancer especially in pediatric cases. The aim of the MRT is to develop a method for radiosurgery of inoperable brain tumours like high-grade gliomas and paediatric tumours of central nervous system, where existing modalities fail to deliver the adequate results. Experiments with normal rat-brain have displayed unusually high resistance to necrosis when irradiated with such beams having dimensions of few tens of micrometers in width and delivered at skin-entrance absorbed doses of 312 Gy to 5000 Gy, which appears quite fascinating since the current broad beams employed for the treatment purpose have upper limit of absorbed doses in tens of Gy (i.e., 50-60 Gy). It is important to establish reliable calculational tools to plan radiotherapy with Microbeams and it is with this aim that current study has been undertaken at Centre for Medical Radiation Physics (CMRP), University of Wollongong, to evaluate the potential of Microbeam Radiotherapy and analyse the suitability of Monte Carlo code GEANT4 to adequately model photon/electron transport for such microbeams from synchrotron and compare with experimental data where available to understand the radiobiological effects of microbeams. This will help in designing the therapeutic parameters for this emerging modality.

**Materials and Methods:** MOSFET, for real time dosimetry at ESRF (European Synchrotron Radiation Facility) utilize the REM quadruple MOSFET detector. Two of the FETs have an oxide thickness of about 1 micron. The Si body of the MOSFET is about 500 micron x 1000 micron x 1000 micron. A filled epoxy globule of known composition covers the FETs. Monte Carlo simulations of deposited doses in the gate oxide were performed using Edge-On mode of MOSFET detector. Beam strike the detector parallel to the surface. Detector is placed in a PMMA phantom and the beam cross section is only 25 micron x 500 micron. Simulations were performed using Monte Carlo code GEANT4. The GEANT4 Monte Carlo toolkit simulates the interaction of fundamental particles with matter. It is based on Object Oriented Programming and is composed of a collection of C++ classes, each representing a particular aspect of the simulations process (geometry, tracking, visualization, etc.). Simulations were performed for lateral dose profile of a 100 keV single microplanar beam in the phantom.

**Results and Discussion:** The dose profile shows asymmetry, a shift in the maximum dose point of the dose profile obtained for the Edge On MOSFET. By contrast, the dose profile at depth 7 cm in a PMMA phantom is symmetrical. The shift to the right of about 5 micron can be explained as related to dose enhancement effect arising from laterally scattered electrons from the Si body entering the $SiO_2$. There is a lack of laterally scattered electrons from the epoxy arising from poor electronic equilibrium.

### Reference

1. Brauer-Krisch E, Bravin A, Lerch M, Rosenfeld AB, Stepanek J, DiMichiel M, *et al*. MOSFET dosimetry for microbeam radiation therapy at the European synchrotron radiation facility. Med Phys 2003;30:583-9.

### O-12

### Study on uncertainties in dose calculation in heterogeneous region by different calculation algorithms and correction methods

A Sankar*#, PGG Kurup*, V Murali*, B Arun*, S Mahalakshmi*, J Velmurugan#. **Apollo Speciality Hospital, Chennai, #Department of Physics, Anna University, Chennai, India*

**Introduction:** Three-dimensional (3-D) radiotherapy treatment planning system derives the patient heterogeneity details from CT data. This study investigates the limitations of the dose calculation in heterogeneous medium by different correction methods employed in four commercially available treatment planning systems for 6 MV photon beams.

**Materials and Methods:** Two phantoms were considered for this study. One is the in-house made layered Perspex phantom of size 24x24x12 $cm^3$. An air cavity of dimension 5.5x5x4.5 $cm^3$ was created in the middle of the phantom to simulate the air-soft tissue geometry. Small holes of diameter 2 mm are made along the depth and cross profile of the phantom to accommodate micro Thermoluminicent Dosimeter (TLD) cubes for point dose measurement. The second one is the MED-TEC IMRT phantom, which is having a bone equivalent cylindrical shaped material of diameter 3 cm and length 5 cm to simulate the bone-soft tissue geometry. Facilities are provided to do the ionchamber measurement at the center and TLD measurements at different points of the high dense medium. Four commercially available treatment planning systems were used for the dose calculations in the

This PDF is available for free download from a site hosted by Medknow Publications (www.medknow.com).

[Downloaded free from http://www.jmp.org.in on Friday, July 29, 2011, IP: 130.14.254.25] || Click here to download free Android application for t above specified geometry. Three of the planning systems are using Pencil beam algorithm for dose calculation but handles the tissue heterogeneity in different ways, one is using Equivalent Tissue-Air Ratio (ETAR) and other two are using equivalent pathlength method correction methods. The fourth planning system uses the Collapsed Cone Convolution algorithm for dose calculations. A single beam configuration of field size 6x10 $cm^2$ was used so that, part of the beam passing through homogeneous soft tissue and part of the beam through soft tissue-air-soft tissue medium. For bone-soft tissue geometry field size of 6x6 $cm^2$ was used. Dose measurements were made using radiochromic film and TLDs in air-soft tissue geometry. Ion chamber and TLDs were used for dose measurements in bone-soft tissue medium. The Monitor Units (MU) calculated for a prescribed dose by different planning systems were compared. Calculated dose profiles along homogeneous and heterogeneous regions were compared with the profiles measured using TLDs and radiochromic film.

**Results:** All the planning systems show good agreement with the measured dose profile along depth and across the field in the homogeneous soft tissue region. But large deviations were observed between calculated and measured dose profiles in the cavity. All the correction methods failed to predict the penumbral broadening of the photon beam when it traverse from high dense to low dense medium. It was observed that the deviation increases, for given energy and cavity dimension, as the depth in the cavity increases. A difference of 20-25% and 15-20% was observed for equivalent pathlength and ETAR correction methods respectively with measured profiles using TLDs and Radiochromic film. The collapsed cone convolution algorithm predicts the penumbral broadening with reasonable accuracy and it shows a difference of 4-6% with measured dose profile. There is a difference of -1% between the calculated and measured dose using ion chamber in the center of the high dense medium. The TLD measurements are also within ±3% of the calculated dose at different points.

**Conclusions:** Potential under dosage is possible because of the penumbral broadening of the beam in low dense medium. Collapsed cone convolution algorithm predicts the dose distribution in air-soft tissue geometry with good accuracy when compared to other studied correction methods. In the case of bone-soft tissue geometry all the correction methods show good agreement among them and with measured point doses.

### O-13
### Commissioning and quality assurance of a commercial intensity modulated radiotherapy (IMRT) treatment planning system preciseplan

SM Pelagade, KK Thakur, TT Bopche, DC Bhavsar*, RP Shah, RK Vyas*. *Department of Medical Physics, *Radiation Oncology, The Gujarat Cancer and Research Institute, NCH Campus, Asarwa, Ahmedabad - 380 016, India*
Corresponding author: Dr. S.M. Pelagade,
E-mail: pelagade_satish@yahoo.com

**Introduction:** The commissioning of the dose calculation algorithms of a treatment planning system is generally performed by entering the basic beam data into the system according to the methods and requirements described in the system user manual and by comparing the results of dose calculations with the entered data and with data that were measured specifically for this purpose. The purpose of this paper is to present our experience with the commissioning and QA of preciseplan treatment planning system with multileaf collimator (MLC) using step and shoot IMRT technique.

**Materials and Methods:** A new dedicated intensity modulated radiotherapy (IMRT) unit Elekta's (precise) linear accelerator was used in this study. The blue phantom™ radiation field analyser was accurately positioned in the treatment room and left there for at least 2 hours in order to reach temperature stability. A 3D treatment planning system (TPS) for intensity modulated radiotherapy (IMRT) using a multileaf collimator (MLC) has been made available by elekta. Two techniques of computerized treatment planning for step and shoot IMRT are generally applied: The first method is an extension of conventional treatment planning and is referred to as forward planning.[1] The second strategy, which we denote as inverse planning, usually starts with the optimization of fluence profiles from each beam direction by minimization of an objective function. Afterwards, sequencing transforms each optimized profile into a series of segments, which can be delivered with a multileaf collimator (MLC). The various dosimeric parameters like depth dose data, profiles, output factors, scatter factors, bulk leaf transmission and back up jaw transmission were measured for the purpose of modeling the 6mv photon beam for the treatment planning system used in this study.

**Results and Discussion:** A. Dosimetric Analysis: The larger field sizes have higher PDD values for our dedicated system, the variation in PDD is limited to 3% between the 3 cm x 3 cm and 40 cm x 40 cm open field sizes at depth of 10 cm. The percentage depth dose for wedged field sizes was measured and the variation found to be 4.2% between the 3 cm x 3 cm and 30 cm x 40 cm wedged field sizes at depth of 10 cm. The OAR were computed from the beam profiles for 10 cm x 10 cm, 20 cm x 20 cm and 30 cm x 30 cm field sizes at 10 and 20 cm depths in the x (crossline) and y (inline) directions and were found within ±0.2 mm. The relative output factors (OF) for different field sizes and the scatter factor $S_c(r)$ for open field sizes were measured.

B. Quality -Assurance of dose calculation by measurements

B.1. Point dose calculations: The absolute point dose of the treatment plan was measured by using Head and Neck Cube supplied by Scanditronix Wellhofer. The same plan executed on machine with same phantom by inserting 0.65cc active volume Farmer type ion chamber (FC65-G). The dose at isocenter was measured. The calculated and the measured doses were compared.

B.2. Isodose distribution comparison: The torso phantom (Scanditronix Wellhofer's) was used to compare the calculated and the measured dose distributions. the film (Kodak EDR) was stacked in between the two slabs. Isodose distributions were measured using Kodak EDR film. The exposed film was scanned with vidar scanner and the distributions were seen through Omnipro IMRT software. The comparisons between the calculated and the measured dose distributions were made within Omnipro IMRT software system.[2] It is seen that the agreement between the calculated and the measured dose is within 2% in the high-dose region. for clinical patient specific QA we specify 3% dose difference and 3 mm distance acceptance scaling criteria.[3] The bulk leaf transmission and back up jaw transmission were 0.02% and 0.105%.

**Conclusion:** Treatment-planning system configuration parameters must be measured precisely. The TPS calculated dose and the measured absolute dose should not deviate more than 3% to ensure safe treatment. It is necessary to maintain strict criteria to compare measured and calculated values. The introduction of new analyzing tools, such as DTA (distance to agreement) or G (gamma factor), correlation coefficient can be useful to better quantify the comparison between measured versus calculated dose distributions.[4,5] The implementation of IMRT must not be underestimated. Every institution should adopt a QA protocol.

### References

1. Bar W, Alber M, Nusslin F. A variable fluence step clustering and segmentation algorithm for step and shoot IMRT. Phys Med Biol 2001;46:1997-2007.
2. Harms WB, Low DA, Purdy JA, Wong JW. A quantitative software tool for verifying 3D dose calculation programs. Int J Radiat Oncol Biol Phys 1994;30:187.
3. Van Dyk J, Barnett RB, Cygler JE, Shragge PC. Commissioning and quality assurance of treatment planning computers. Int J Radiat Oncol Biol Phys 1993;26:261-73.
4. Cheung KY. Intensity modulated radiotherapy: Advantages, limitations and future developments. Biomed Imaging Interv J 2006;2:e19.
5. Agazaryan N, Solberg TD, DeMarco JJ. Patient specific quality assurance for the delivery of intensity modulated radiotherapy. J Appl Clin Med Phys 2003;4:40-50.

This PDF is available for free download from a site hosted by Medknow Publications (www.medknow.com).

[Downloaded free from http://www.jmp.org.in on Friday, July 29, 2011, IP: 130.14.254.25] || Click here to download free Android application for t

**O-14**

**Quality assurance of IMRT patient specific plans**

Suresh Chaudhari, Sudesh Deshpande, V Anand, Sandeep De and V Kannan. *Department of Radiation Oncology, P.D. Hinduja National Hospital and Medical Research Center, Mahim, Mumbai, India*

**Introduction:** Quality assurance (QA) of Intensity modulated radiotherapy (IMRT) using dynamic multileaf collimator (dMLC) is critical to ensure accurate delivery of optimized treatment plans. IMRT QA is divided into machine specific and patient specific tests. In this study we report results of patient specific tests for IMRT. Our patient QA procedure consists of an absolute dose measurement for all treatment fields in the treatment setup and film relative dose determination for the treatment plan. The tests performed revealed acceptable results. **Materials and Methods:** The equipment used are, Brainscan 5.3 (BrainLAB AG, Germany) inverse treatment planning system (TPS), configured for 1-cm width Multileaf collimator (80 leaves), dual energy Linear accelerator 2300CD, which are interfaced with Varis Vision network (Varian, Palo Alto, USA). To start with IMRT plan an appropriate arrangement of static beams is required for inverse planning. After defining the calculation parameters and constraints with respect to target and organs at risk (OAR), the inverse planning optimization generates 4 plans simultaneously with different Target and OAR priorities. It uses "Dynamically Penalized Likelihood algorithm" for inverse planning optimization and pencil beam convolution for forward planning calculation. Among the four plans best-suited plan is selected. For QA, the solid water Phantom (Scanditronix-Wellhofer, Germany) was scanned with 3-mm slice thickness. The dimension of Phantom was 18x18x18 $cm^3$, which simulates head of the patient. For Pelvic simulation two lateral scatters were used. The ion camber insert was placed at 5 cm depth. Once a plan is approved all dMLC files were transferred on IMRT QA phantom with the planned gantry angles and monitor units. Dose distributions were calculated on phantom and point dose at isocentre is obtained. 0.65cc and 0.01cc farmer type ionization chambers (Scanditronix-Wellhofer) were used for absolute dosimetry. The dose calculated by TPS was compared with the measured dose. The absolute dose measurement at central axis was considered adequate if the difference between the calculated and the measured dose was <5%. If the difference between measured and calculated dose exceeds 5% additional point dose measurement for individual field was also performed at 5-cm depth at gantry angle zero. For individual field dosimetry ±3cGy was passing criterion. Relative dosimetry was done by irradiating a Kodak EDR2 film placed perpendicular to the incident beam in a solid water phantom at 5-cm depth. Film dose distributions were analyzed using Omnipro IMRT software (Scanditronix-Wellhofer) and film scanner VIDAR DosimetryPro. For accurate alignment of the film on the densitometer four standard points were marked on the film along X and Y coordinates. As a department protocol, we verify only composite plans on phantom and not individual fluence. Films were analyzed with Gamma index evaluation, DTA and isodose line matching. An acceptance criterion for the planar dose was developed: 3% delta dose, 3 mm DTA, Spatial isodose lines > 50% must match within 2 mm.

**Results and Conclusion:** Of the 20 evaluated plans 12 plans were within 3% dose variation, 3 plans were within 3-5% variation and 5 plans were above 5%. Individual field dosimetry at gantry angle zero for these 5 plans showed maximum variation of 2.75 cGy. Since QA was performed in treatment condition, posterior oblique fields were attenuated by treatment couch, which may be reason for variation more than 5%. Positioning the patient on extended carbon fiber couch for daily treatment eliminated this beam attenuation problem. Gamma index evaluation method reveals on an average 95.4% of the data passes the criterion with standard deviation of 3.58. The isodose line matching was within acceptable limits.

**O-15**

**Entrance surface doses in majorly performed diagnostic X-ray examinations**

Reena Bohra, V Jayalakshmi, SD Sharma. *Radiological Physics and Advisory Division, BARC, Mumbai, India*

**Introduction:** Entrance surface dose (ESD) is one of the basic dosimeteric quantities for estimation of the patient dose. Optimization of patient dose and comparison with the international reference values

This PDF is available for free download from a site hosted by Medknow Publications (www.medknow.com).

Table 1: ESDs for different types of X-ray examinations with reference value and range factor

| *Type of exam.* | *Range of age of patient (Yrs.)* | *Range of thickness of patient (cm)* | *Range of kVp* | *Maximum. ESD (mGy)* | *Minimum ESD (mGy)* | *Mean ESD (mGy)* | *Reference value[2] (mGy)* | *ESD range factor (Max/Min)* |
|---|---|---|---|---|---|---|---|---|
| Chest | 15-65 | 8-20 | 48-60 | 0.21 ± 0.11 | 0.05 ± 0.01 | 0.16 ± 0.1 | 0.4 | 10.4 |
| PA | 18-60 | 10-22 | 45-72 | 0.52 ± 0.18 | 0.09 ± 0.03 | 0.22 ± 0.09 | | |
| | 20-65 | 12-24 | 55-78 | 0.18 ± 0.20 | 0.07 ± 0.01 | 0.14 ± 0.10 | | |
| | 18-58 | 10-26 | 58-80 | 0.23 ± 0.30 | 0.13 ± 0.10 | 0.21 ± 0.18 | | |
| | 22-70 | 9-24 | 62-78 | 0.21 ± 0.22 | 0.15 ± 0.09 | 0.18 ± 0.15 | | |
| Lumbar | 15-60 | 10-25 | 70-80 | 8.69 ± 0.65 | 7.03 ± 0.20 | 7.76 ± 0.80 | 10 | 4.2 |
| Spine | 18-62 | 12-20 | 60-75 | 12.68 ± 0.8 | 9.0 ± 0.80 | 10.89 ± 0.60 | | |
| AP | 22-60 | 15-20 | 60-80 | 7.68 ± 0.50 | 6.0 ± 0.08 | 6.56 ± 0.20 | | |
| | 24-55 | 12-20 | 60-90 | 8.03 ± 1.0 | 3.0 ± 0.09 | 6.18 ± 0.50 | | |
| | 20-70 | 15-18 | 68-88 | 8.5 ± 0.40 | 6.0 ± 0.10 | 7.4 ± 0.30 | | |
| Lumbar | 18-60 | 15-30 | 60-78 | 8.20 ± 0.4 | 5.28 ± 0.30 | 7.19 ± 0.50 | 30 | 5.68 |
| Spine | 15-50 | 20-30 | 62-88 | 30.0 ± 1.2 | 8.68 ± 0.90 | 20.92 ± 2.0 | | |
| Lateral | 18-62 | 22-32 | 58-90 | 12.0 ± 0.8 | 8.05±0.50 | 10.26 ± 1.0 | | |
| | 22-65 | 20-34 | 72-95 | 7.4 ± 0.6 | 7.1 ± 0.20 | 7.23 ± 0.80 | | |
| | 20-70 | 15-30 | 58-80 | 8.5 ± 0.21 | 7.8 ± 0.50 | 8.0 ± 0.35 | | |
| Thoracic | 18-60 | 10-20 | 62-78 | 7.64 ± 0.2 | 4.52 ± 0.30 | 7.39 ± 0.30 | 7 | 2.21 |
| Spine | 15-50 | 15-20 | 68-80 | 9.86 ± 0.30 | 6.9 ± 0.40 | 8.88 ± 1.0 | | |
| AP | 18-62 | 12-20 | 62-80 | 7.23 ± 0.40 | 7.1 ± 0.30 | 6.9 ± 1.1 | | |
| | 22-65 | 12-22 | 60-88 | 8.34 ± 0.60 | 7.89 ± 0.50 | 7.5 ± 0.30 | | |
| | 20-60 | 10-25 | 55-86 | 5.00 ± 0.20 | 4.25 ± 0.30 | 4.8 ± 0.25 | | |
| Thoracic | 15-65 | 15-30 | 60-90 | 10.23 ± 0.5 | 6.44 ± 0.40 | 8.44 ± 1.0 | 20 | 2.86 |
| Spine | 22-60 | 16-32 | 58-88 | 18.44 ± 0.8 | 8.4 ± 0.60 | 12.69 ± 0.90 | | |
| Lateral | 24-62 | 15-28 | 60-78 | 7.44 ± 0.40 | 5.68 ± 0.40 | 6.44 ± 0.50 | | |
| | 18-60 | 15-31 | 68-92 | 8.66 ± 0.80 | 7.69 ± 1.0 | 7.5 ± 0.30 | | |
| | 18-58 | 16-32 | 60-94 | 9.50 ± 0.40 | 7.89 ± 0.80 | 8.05 ± 0.55 | | |

AP - Anterior-posterior, PA - Posterior-anterior

[Downloaded free from http://www.jmp.org.in on Friday, July 29, 2011, IP: 130.14.254.25] || Click here to download free Android application for t is possible only by measuring the ESD of patients.[1] ESD measurement in different radiology departments and comparison between them may be used to assess the overall condition existing in the radiology departments and implement corrective measures if needed. Chest radiography is the most common examination performed in radiology followed the lumber spine radiography. The present study is aimed at evaluation of the ESD in five major hospitals of Mumbai.

**Materials and Methods:** ESD measurements were carried out by using TLDs CaSO4: Dy discs (6mm dia and 0.8 mm thickness) and LiF: Mg, Cu, P chips (3.2x3.2x0.48 mm). The TLDs were cleaned, annealed and packed in polythene pouches of 5 TLDs each. The pouches were placed on the patient at the central axis of the primary X-ray beam. The exposed TLDs were then read out using a calibrated TLD reader. Calibration of the TLD reader was done using the calibrated ionization chamber Nero mAx 8000 in diagnostic energy region. A total of 50 patients were considered in each type of exam [Table 1]. Set exposure time and focus to film distance (FFD) in chest radiography were found ranging from 0.1 to 0.4 sec, 180 cm and for skeletal examinations, 0.18 to 0.66 secs, 90-100 cm respectively.

**Results and Conclusion:** Large variations in ESD values have been observed for same type of X-ray examination in the different hospitals even though many times, the thicknesses of the patients were same. ESD in each examination were found below the reference values specified by IAEA. The ratios of maximum to minimum ESD values are representative of the overall picture of the conditions existing in the various radiology departments. It was also observed that large variations in ESDs were due to different procedures adopted by the hospitals e.g., X-ray equipment variability, operating conditions, imaging devices (type of screen-film combinations), processing conditions etc.

We can conclude from this study that by proper selection of above procedures, dose to the patient as well as collective dose can be considerably reduced without compromising the image quality.

### References

1. Zoetelief J. Recommendations for patient dosimetery in diagnostic radiology using TLD. Eur Comm Nucl Sci Technol 2000.
2. International Basic Safety Standards for Protection against Ionizing radiation and for the Safety of Radiation Sources, Safety Series No. 115, IAEA Vienna 1996: When film is used in combination with 200 speed class screen.

### O-16

### Segmentation of brain tumors in perfusion and diffusion weighted magnetic resonance images using artificial neural networks

C Vijayakumar, Gharpure Damayanti Chandrashekhar*, CM Sreedhar, Sunil Bhargava. *Department of Radiodiagnosis and Imaging, Armed Forces Medical College, Pune, *Department of Electronic Science, University of Pune, Pune, India*
Corresponding and Presenting Author: C. Vijayakumar
Department of Radiodiagnosis and Imaging Armed Forces Medical College, Pune, India
E-mail: vijayafmc@gmail.com / vijay_kumar_c2000@yahoo.com

A novel algorithm is proposed for performing the identification and segmentation of brain tumors in magnetic resonance images based on their vascular and cellular information provided by perfusion and diffusion weighted images. Different architectures of artificial neural network have been implemented in performing the segmentation of brain tumors and their performance has been compared. The architectures used are Multi Layer perceptron, Radial Basis function and Self organizing maps. These algorithms are trained to recognize and segment the brain tumors based on the vascular and cellular information provided by the reconstructed images of perfusion and diffusion weighted magnetic resonance images. The reconstructed images of perfusion weighted images such as relative cerebral blood volume (rCBV) image, time to peak (TTP) image and percentage of base at peak (PBP) image along with apparent diffusion coefficient (ADC) maps of diffusion weighted images has been utilized. Multi Layer perceptron with one hidden layer has been utilized. The back propagation - gradient desent method with momentum has been utilized to train the multi - layer perceptron. For Self organizing maps, a novel segmentation algorithm based on the number of hits of the Best Matching Units (BMU) is proposed. The radial basis function has been trained with similar patterns used for both self organizing maps and multi layer perceptron. The results shows that the artificial neural network performs well in differentiating the tumor, edema, necrosis, CSF and normal tissues in reconstructed images of perfusion and diffusion weighted magnetic resonance images.

### O-17

### Effect on the breast entrance dose due to change in KVP and the breast thickness

Kanta Chhokra, V Jayalakshmi*, Reena Sharma*. RSD, Atomic Energy Regulatory Board,*RP and AD, Bhabha Atomic Research Centre, Mumbai, India

**Introduction:** The mean absorbed dose to the glandular tissue which is most radiation sensitive is directly proportional to the breast entrance dose. Therefore the aim of the study was to find the effect on breast entrance doses for varying breast thickness and at different kVp.

**Materials and Methods:** For the study a locally fabricated PMMA phantom representing a breast of composition 50/50 was used. The breast phantom consists of 12 semi circular discs of diameter varying from 9 cm to 21 cm and increasing in thickness by 5 mm at each step. A dedicated indigenous mammography unit was used for exposures. A quality control check performed on the unit indicated the system confirmed to the acceptable limits. A calibrated external mammography

This PDF is available for free download from a site hosted by Medknow Publications (www.medknow.com).

Table 1: Relative entrance doses for various phantom thickness at 26kVp

| *Phantom thickness (mm)* | *Relative dose* |
|---|---|
| 10 | 0.448 |
| 20 | 0.524 |
| 30 | 0.672 |
| 40 | 1.00 |
| 45 | 1.298 |
| 50 | 1.582 |
| 55 | 1.925 |
| 60 | 2.224 |

Table 2: Relative entrance doses for various phantom thickness at 28kVp

| *Phantom thickness (mm)* | *Relative dose* |
|---|---|
| 10 | 0.621 |
| 20 | 0.682 |
| 30 | 0.758 |
| 40 | 1.00 |
| 45 | 1.258 |
| 50 | 1.546 |
| 55 | 1.969 |
| 60 | 2.364 |

Table 3: Relative entrance doses for various phantom thickness at 32 kVp

| *Phantom thickness (mm)* | *Relative dose* |
|---|---|
| 10 | 0.750 |
| 20 | 0. 775 |
| 30 | 0.831 |
| 40 | 1.00 |
| 45 | 1.175 |
| 50 | 1.338 |
| 55 | 1.650 |
| 60 | 1.912 |

[Downloaded free from http://www.jmp.org.in on Friday, July 29, 2011, IP: 130.14.254.25] || Click here to download free Android application for t ionization chamber (Victoreen Model 6000-529) having volume 3.3 cc, diameter 4 cm and thickness 2.5 cm and energy response with in ±5% for half value layer in Al of 0.2 to 0.5 mm (16 to 90kVp) was used with calibrated Victoreen dose calibrator model 8000 Nero ™ mAx for the purpose. The calibration of the measuring system is traceable to National Institute of Standards and Testing (NIST). A comparison and checking of the calibration of the system was done at Radiation Safety and System Division, BARC for energy range from 22kV to 40 kVp and the system performance was found to be with in ± 2%. The measurements were carried for phantom thickness ranging from 10 mm to 60 mm representing an equivalent of 1.1 cm to 7.5 cm compressed breast thickness of composition 50% adipose and 50% glandular tissues and for three different KVp normally used for clinical purposes i.e., 26kVp, 28kVp, 32kVp. The unit was set in the semi automatic mode where the kVp was manually selected and the mAs was automatically selected by the unit. All the entrance doses were normalised to 40 mm phantom thickness, which represents the standard compressed breast of 45 mm thickness and composition 50/50.

**Results and Conclusion:** It is estimated that with increase in thickness of the breast, the relative entrance doses increases for the same kV. With increase in kV, for same breast thickness, the entrance dose increases up to 50 mm thickness. However for thickness greater than 50 mm, the relative entrance dose is maximum at 28kVp. These results are indicated in the Tables below. Since, the dose to glandular tissues which are most sensitive to radiation, is directly proportional to entrance surface dose, it is very essential, the radiologist selects proper kVp depending on the compressed breast thickness of each female patient undergoing mammography. As it has been observed that some mammography centers use only two kVps for all female patients in semi automatic mode where mAs is selected by the unit. This has resulted in higher mAs values and thereby higher breast entrance doses of 13mSv, where as 4mSv is recommended as the entrance dose per view film by ICRP.

### References

1. National Commission on Radiological Protection Report 149. A Guide to Mammography and other breast Imaging Procedures. NCRP December 31,2004.
2. Department of Health and Human Services, Food and Drug Administration, 21CFR part 900,section900.12(e)5(i),1997.

### O-18

### An overview of computer aided image segmentation analysis for breast tumors in mammogram

Gurpreet Singh, Pratik Kumar, Sanjay Thulkar*, Rajinder Parshad#.
*Medical Physics Unit (IRCH), *Dept. of Radio Diagnosis (IRCH), #Dept. of Surgery, All India Institute of Medical Sciences, New Delhi - 110 029, India*

**Introduction:** Digital computers are now an integral part of medical imaging process. Images must be in a digital form to be processed by a computer. A digital image consists of a matrix in which each element is represented by a numerical value. The revolution in digital computer technology has made possible new and sophisticated imaging techniques. In mammography computer vision techniques have been used successfully to detect tumors on digital images. This technique therefore decreases errors in mammography interpretations. Image processing is the first step in most detection algorithms based on computer vision. Such processing allows signal to noise characteristics of certain findings in the image to be enhanced, while unwanted detail is suppressed. If images are taken with the digital camera then medical image analysis faces tougher challenge. Since medical images have a poorer noise to signal ratio. The spatial resolution and contrast between anatomical structures is often too low to be computed reliably using a standard image processing technique. Mammography X-ray is only diagnostically valuable if the resolution and spatial accuracy is sufficient to capture attenuation due to micro calcifications. In the current study our main aim is to study the computer aided detection system for breast cancer tumors.

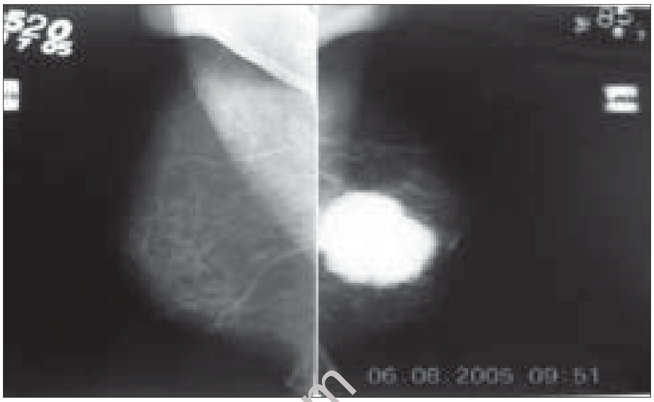

Figure 1: Image to be processed

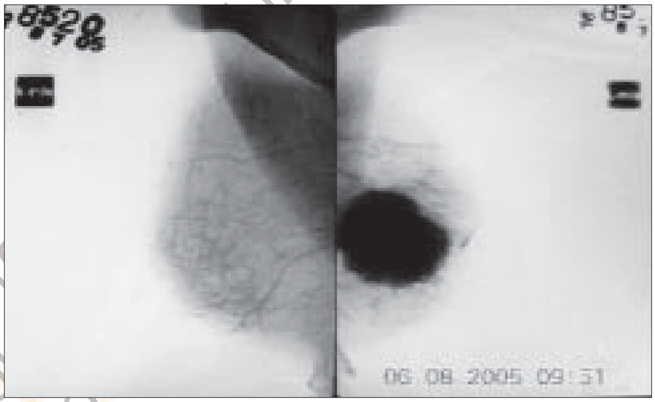

Figure 2: Image after processing

**Materials and Methods:** For this Matrix Laboratory language has been used. MATLAB codes have been developed. The computer algorithm operates on digitized mammography images. In the algorithm image has been read using MATLAB function. Complement of the image has been taken. The object to be segmented differs greatly in contrast from background images. Changes in contrast can be detected by operators that calculate the gradient of an image using the sobel operator which creates a binary mask using a user specified threshold value. Thresholding of the image to show small structures has been done. Compared to the original image there are the gaps in the lines surrounding the object in gradient mask. The linear gaps are made to disappear with the sobel operator. In order to make the segmented object look natural, we smooth the object by eroding the image with a diamond structuring element and close the threshold image.

**Discussions and Conclusions:** The study demonstrated that computer analysis can render substantial help to the radiologist's screening efficiency. Computer vision techniques have the distinct advantages of being as reproducible due to the underlying computer code on which they are based. Most methods require that a number of empirical decisions be made regarding parameters that occur during the execution of the programs, such as filters characteristics of threshold level. Moreover, the computer aided diagnosis is based upon algorithm and hence impervious to day to day variations in human's interpretations. Such consistency in performance can be of great value to the radiologist, who operates in a very different environment. Further work has to be done to improve the accuracy and reproducibility of radiological images. Computer aided diagnosis has to come up in practical terms so that it may be used routinely by the radiologists in a clinical set up. This paper is a first step towards this direction.

### O-19

### Indigenous software for computer aided analysis of medical images using artificial neural network

Neeraj Sharma, Amit K Ray, Satyajit Pradhan*, Shiru Sharma,

[Downloaded free from http://www.jmp.org.in on Friday, July 29, 2011, IP: 130.14.254.25] || Click here to download free Android application for t

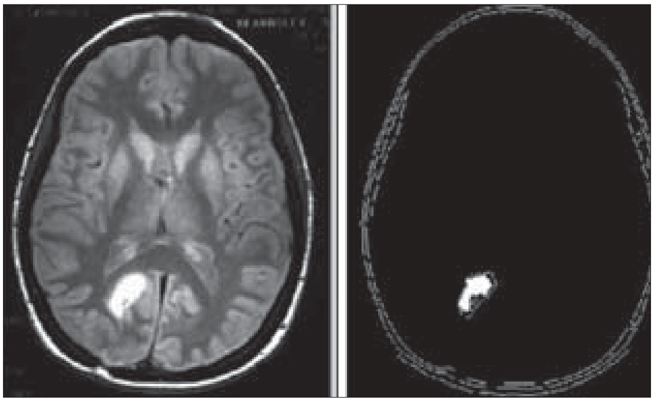

**Figure 1: System configuration**

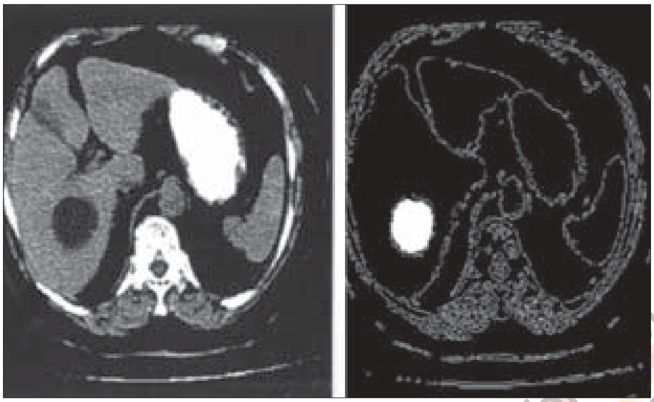

**Figure: 2 (a) Segmentation and characterization of brain (b) Segmentation and characterization of cyst in liver**

Lalit M Aggarwal*. *School of Biomedical Engineering, Institute of Technology, *Department of Radiotherapy and Radiation Medicine, Institute of Medical Sciences, Banaras Hindu University, Varanasi (UP) - 221 005, India*

**Introduction:** In recent years, considerable efforts have been made in computer aided diagnosis (CAD) of medical images to improve clinician's confidence in the analysis of medical images. Evaluation of medical images by clinician is qualitative in nature and may vary from person to person. Several techniques using multiple computer extracted features, pixel value based mutual information using Statistical approach, Syntactic or Structural approach and Spectral approach have been tried. As a comparison between the above mentioned three approaches spectral frequency based method are less efficient, while statistical methods are particularly useful for random patterns/textures. For complex patterns, syntactic or structural methods give better results.
**Aims and Objective:** The objective of developing the software is to achieve auto segmentation and tissue characterization. Therefore, we have designed and developed an algorithm for analysis of medical images based on hybridization of syntactic and statistical approaches, using Artificial Neural Network (ANN). This algorithm performs segmentation and classification in a similar way as is done in human vision system which recognizes objects, perceives depth, identifies different textures, curved surfaces or a surface inclination by texture information and brightness.
**Materials and Methods:** Figure 1 shows the flow chart of the software. Bidirectional Associative Memories (BAM) network was used for feature extraction and classification of medical images. Associative network performs function similar to the human brain property of association and can recall Region of Interest (ROI) even in the presence of artifacts. In development of this algorithm ANN-1 was used for correlation feature extraction which is a specifically modified version of sBAM network and is capable of processing real coded, grey level images. The ANN2 was used for feature vector classification of images. The sBAM based algorithm has been modified for processing grey scale images having real coded values as follows where P is tissue template (matrix of primitive texture cell) defined in supervised manner and pixels are real valued on the scale of 0 to 1. Mean and Standard Deviation features were computed for primitive texture cell. Normalization of P (Primitive texture cell) was done. Extraction of correlation feature by ANN1 was obtained by the matrix I of the image Inner product (Correlation feature) = b [r and s are indices of pixel] Gray Level Cocurance Matrix (GLCM) feature (Angular Second Moment, Entropy, Homogeneity and Cluster Tendency) were extracted and averaging was done for 3x3 Matrix window for the distance 1 and 2 in the direction 00, 450, 900 and 1350. Finally, the image was resynthesized based on the classification data obtained by ANN-2. The threshold value corresponding to 50% of confidence level was applied for segmentation. The programming of the algorithm has been done in MATLAB.
**Results and Conclusions:** The software developed was first tested on different Markov textures to test the performance and the success rate achieved was 100%. The software was also able to detect distorted Markov texture cells impregnated with noise and the level of distortion. The software was used in analysis of CT images of liver and brain to detect known abnormalities [Figure 2]. The results were clinically evaluated and found in agreement with the radiological diagnosis. Finally it can be concluded that this software would be of great help in diagnosis and treatment. It would be an effective tool in auto contouring and evaluation of radiotherapy outcome by evaluating CT/MRI images.

**O-20**
**Dosimetry of large field photon irradiations at extended treatment distances with linear accelerator**

R Ravichandran, K Krishnamurthy, SS Sivakumar, CA Davis, BK Mohanti, Kamal El Ghamrawy. *Department of Radiotherapy, National Oncology Center, Ministry of Health, Muscat, Sultanate of Oman*

**Introduction:** The hemi-body or total body irradiations (TBI) with 6MV X-rays are commonly used in the management of systemic malignant spread and bone pain due to metastases. To get magna fields to cover the entire body length, Focus Skin Distances (FSD) of 3.5 m are used. Dosimetry with large field irradiations are very well discussed.[1,2] In our new oncology center, radiotherapy was started in December 2004. In order to make TBI treatments available, we had undertaken dosimetric measurements.
**Materials and Methods:** The 6 MV X-ray beam from Clinac 600 CD (M/s Varian, USA) has quality factor 0.6718 (TPR20/10). The maximum field size of 40 cm x 40 cm at (1 m), provide size of 198 cm and 226 cm at 3.5 m, 4.0 m respectively. A phantom size of 200 cm x 40 cm was obtained by placing 10 gallons water cans on one side and RFA Blue phantom on other side of a 30 cm x 30 cm x 30 cm solid water phantom (Scanditronix Wellhofer, Germany) supported back side by phantom of plastic water (Computerized Imaging Referenced Systems, USA). DDs and TMRs were measured using 0.6 cc Farmer type ion chamber along with Dose 1 reference class electrometer (M/s Scanditronix Wellhofer, Germany). Inverse square law and conformity of depth dose characteristics of the beam was carried out for Focus

Table 1: Percentage depth doses for magna fields used for total body irradiations

| *No.* | *Depth cm* | *Royal hospital % DD for FSD 4.0 M* | *4.3 M* | *Podgorsak et al 1985 % DD for FSD 4.0 M* | *PDD values used at Gliwice center %DD for FSD 3.75 M* |
|---|---|---|---|---|---|
| 1 | 1.5 | 100 | 100 | 100 | 100 |
| 2 | 5.0 | 92.96 | 92.96 | 93.0 | 92.1 |
| 3 | 10.0 | 80.50 | 80.85 | 80.8 | 79.5 |
| 4 | 16.5 | 65.50 | 65.70 | 65.7 | 65.0 |
| 5 | 20.0 | 57.23 | 59.15 | 58.3 | 57.6 |

This PDF is available for free download from a site hosted by Medknow Publications (www.medknow.com).

[Downloaded free from http://www.jmp.org.in on Friday, July 29, 2011, IP: 130.14.254.25] || Click here to download free Android application for t

Table 2: Measured TMR values for extended distance compared to focus axis distances 1.0 M

| No. | Depth cm | $TMR_{3.5M}$ 1.4 M x1.4 M | $TMR_{4.5M}$ 1.8 M x 1.8 M | $TMR_{1.0M}$ 40 cm x 40 cm | $TMR_{1.0M}$ 40 cm x 40 cm BJR Sup.25 |
|---|---|---|---|---|---|
| 1 | 1.5 | 1.000 | 1.000 | 1.000 | 1.000 |
| 2 | 3.0 | 0.975 | 0.970 | 0.983 | 0.983 |
| 3 | 5.0 | 0.934 | 0.929 | 0.948 | 0.949 |
| 4 | 10.0 | 0.819 | 0.812 | 0.844 | 0.848 |
| 5 | 15.0 | 0.703 | 0.702 | 0.740 | 0.741 |
| 6 | 20.0 | 0.595 | 0.599 | 0.633 | 0.638 |

Axis Distances (FAD) from 1.0 m to 4.5 m. With phantom dimensions 90 cm x 34 cm x 30 cm and plane parallel chamber PPC 40 (Scanditronix Wellhofer) entrance dose was estimated. Measured TMRs were verified using exponential relation, accounting for scatter.

TMR= exp {μ (d-dm)}. PSFR (1)

Measured DDs were compared with the earlier reports (Pogorsak 1985) and values used at another center in Poland for TBI (Instytut Onkologii, Marie Curie Center for Onkologie Gliwice). The dose outputs of the 6 MV beam for the maximum field opening were calibrated at 3.5 M and 4.5 M FAD using TRS 398 protocol of IAEA (2000) with 0.6 cc chamber at 10 cm depth in solid water phantom and Kq factor of 0.9919 and a factor 1.01 for using solid water phantom.

**Results:** Inverse square law was found valid for the large field, for FAD varying from 1.0 M to 4.5 M. The ratios of ionizations 20/15 and 25/15 depths are found constant within 0.1%. Table 1 shows our measured values of DDs for the magna field at 4.0 M and 4.3 M focus skin distances (FSD) and they agreed well with reported values. The measured values of TMR at extended distances are shown in Table 2. These values agree with calculated values using equation (1) within 3%. The calculated monitor units/ 100cGy using %DD and TMRs at depths 5 cm to 20 cm were in agreement within 3%. The measured absorbed dose values at dose maximum plane are Dose/MU = 0.0929 cGy/MU and 0.056 cGy/MU for FAD 3.5 M and 4.5 M respectively, which agree well with calculated values of 0.0925 cGy/MU and 0.056 cGy/MU at these distances. The entrance skin dose for magna field is 79.3% at 400 cm FSD compared to 50% for 10x10 cm field at 100 FSD.

**Conclusions:** We have measured PDDs and TMRs for extended SSDs. As TMRs are independent of FSD, estimating dose to various planes during TBI become easier. These physical parameters will be useful for treatment planning in other institutions, if their 6 MV beam quality is comparable.

**References**

1. Podgorask EB, Pla C, Evans MD, Pla M. The influence of phantom size on output, peak scatter factor and percentage depth dose in large-field photon irradiation. Med Phys 1985;12:639-45.
2. Van Dyk J, *et al.* AAPM Rep 17, 1986.

**O-21**

**Tissue inhomogenity correction dosimetry for indigenously developed homogeneous and inhomogenous thorax human phantom using TLD and 3D TPS**

S Senthilkumar, S Tamilselvan*, S Vasanthamalai, J Jebasingh. *Dept of Radiotherapy, Govt Rajaji Hospital and Madurai Medical College, Madurai - 20, *Dept of Physics, Bharathiar University, Coimbatore, Tamil Nadu, India*

**Introduction:** The human body consists of a variety of tissues and cavities with different physical and radiological properties. Most important among these, from a radiation dosimetry perspective, are tissues and cavities that are radiologically different from water, including lungs and bones. Especially thorax region has more inhomogenity than the other regions of the human body and it consists of different density material such as bone, soft tissue and lungs. Absorption of photons in lung tissue is different from water or soft tissues. The reason is different density, atomic number and electron density of these tissues. To deliver the accurate treatment to the tumor volume, verification of the dose is a must. The radiation dose deposition pattern in radiotherapy is altered by inhomogeneities present in body. The main aim of radiotherapy treatment is to deliver maximum dose to the tumor volume and minimize the dose to the surrounding normal tissues and organs at risk. Therefore to maximize the therapeutic benefit of radiotherapy, it is essential that the absorbed dose delivered to all irradiated tissues in the presence of such inhomogeneities be predicted accurately. The best method for measuring the dose inside the patient is *in vivo* dosimeter. In this study we developed indigenous inhomogenous and homogeneous thorax Indian average adult phantom fabricated with water equivalent bees wax material for tissue and cork used for lung cavity. Finally the dosimetry data's were compared with TLD and 3D TPS.

**Materials and Methods:** The phantom should resemble the human anatomy. So we prepared phantom with the guidelines of CT images of human thorax region. First each slice of the CT images is contoured. The contour consists of body margin, lungs and spine. Applying magnification factor so as to get the exact contour of human thorax region magnifies the contour. The mould is prepared with thermocole. The thickness of thermocole should be of contour separation. The contour is taken for 2 cm separation. Hence themocole with thickness of 2 cm is taken. The exact contour is drawn on thermocole and then cut. The thermocole mould is placed on nonstick sheet and the body margin and lung position are marked on it. The cork shaped as lung in the contour is placed over the marked position in nonstick sheet. Now melted bees wax is poured slowly in the mould without forming air bubbles. Care is taken so that wax is not poured over the cork. The time is allowed to cool the bees wax then the mould is removed so that the beeswax with cork resemble the slice of the phantom. Like this the mould is prepared for all contours and slice of the whole phantom is prepared. Then all the slices of the phantom are placed together in position, which resemble the exact human thorax phantom. Same procedure was repeated for making homogenous phantom except the lung cavity. To measure the point dose inside the phantom TLD capsules were used. In the central slice of the phantom several holes were made at critical locations of the chest wall and lungs and also in the spine and heart position of the both phantoms. Fabricated inhomogenous phantom was placed on the couch of Theratron Phoenix Co-60 machine. TLD capsules were placed in the central slice of the phantom such as Entrance, exit and midpoint, spine, media sternum, 12 points in lungs with various areas, 1 point in heart, 8 points in ribs. After placing the TLD capsule in the inhomogeneous phantom, it was irradiated for different treatment techniques in various conditions. Same procedure was repeated for the homogeneous phantom with the same treatment technique. We have three dimensional (3D) Plato version 3.31. The external contour of the thorax phantom was entered manually through digitizer in to the TPS in order to get an accurate reconstruction of the external contour of the phantom and also digitized lung cavities, spinal cord and various points to get the point dose. Similarly all the slices of inhomogeneous phantom were entered. The mass densities of the wax phantom and lung cavities filled by cork densities were also entered in the TPS. Similar methods adopted to enter the homogeneous phantom external contour, digitized in order to achieve an accurate reconstruction of the experimental geometry of the homogeneous phantom for the entire cavity of the thorax. The measurement points were marked with uncertainty of less than 1 mm for dose calculations. One of the point ® was fixed as a reference in the field to calculate the relative dose for each point. Reference point was fixed at the beam

This PDF is available for free download from a site hosted by Medknow Publications (www.medknow.com).

[Downloaded free from http://www.jmp.org.in on Friday, July 29, 2011, IP: 130.14.254.25] || Click here to download free Android application for t entrance inside the phantom. So the inhomogeneity contribution to this point will be negligible. With this the TPS calculates the tissue inhomogeneity correction based on the EPL (Effective path length).

**Conclusion:** Indigenously fabricated inhomogenous and homogeneous phantoms are irradiated with TLD capsules and the same dose points are calculated from the 3D TPS. Both the TLD and TPS values are compared for homogeneous and inhomogeneous phantoms. The TLD values and TPS calculated algorithm values are within the limit for both the homogeneous as well as heterogeneous phantoms. The variations observed in the TPS values for homogeneous and heterogeneous phantoms were almost same as for TLD values of the homogeneous and heterogeneous phantoms. From this we conclude that the dose calculation by EPL based 3D TPS for thorax cancer radiotherapy treatments are acceptable for dose points and unit density materials. But the inhomogeneity inside the treatment volume will produce changes in the dose distribution. It depends on the density and the type of material and the traveling path of the beam. The thorax region is the main inhomogeneous region. Our study also reveals the same. Whenever planning to deliver radiotherapy treatment to the cancer patient under inhomogeneous conditions inhomogeneity correction factor must be calculated to deliver accurate dose to the tumor volume.

### O-22

### Measurment of back scattered radiation from micro multileaf collimator and secondary collimator jaws into the beam monitor chamber from a dual energy linear accelerator

KR Muralidhar, Tirumalaiswamy, Shiyama, Madhusudhan Sresty Rajanesh, Pramod, Akraju. *Indoamerican Cancer Institute and Research Center, Road No 14, Banjara Hills, Hyderabad - 500 034, India*
E-mail: krmuralidhar@rediffmail.com

**Introduction:** The Photons and electrons backscattered from the micro Multileaf collimator, upper and lower secondary collimator jaws give rise to a significant increase in the ion charge measured by monitoring chamber. This increase varies between different accelerators. We have studied the effect of backscatter into the monitor chamber at 6 MV photon energy for the Siemens primus plus linear accelerator.

**Materials and Methods:** Variation of the output factor was first studied for variable field sizes of mMLC together with fixed collimator jaws of size 10x10 cm$^2$ and also for fixed mMLC field size (4x4 cm$^2$) with variable field sizes of secondary collimator. The output measurements were carried out at Dmax in a water phantom at central axis. Backscatter radiation was analyzed. Results: In the first experiment it was noticed that the output factor is almost constant from 4x4 cm$^2$ to 10x10 cm$^2$ of mMLC field sizes by keeping secondary collimator field size 10x10 cm$^2$ constant. The decrease in output factor observed

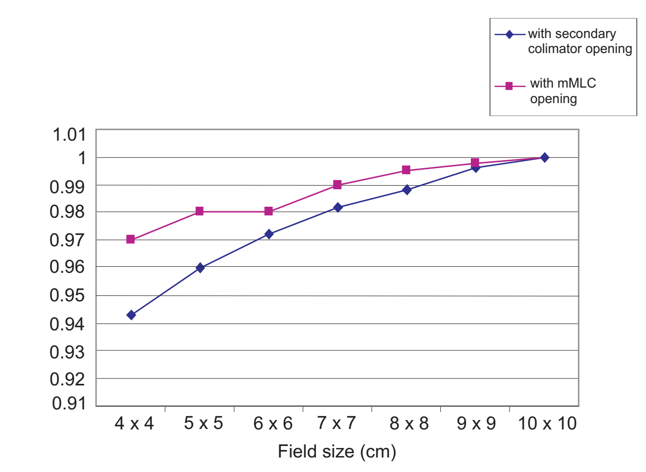

**Figure 1: Variation of output factor with field size**

is 3% for 4x4 cm$^2$ field size. This data is for 6MV photons with acculeaf, mMLC in siemens primus plus linear accelerator. In case of fixed collimator field size and different field sizes of mMLC i.e., from 4x4 cm to 10 x 10 cm, the output factor decreases to 6%.

**Conclusions:** It can be concluded that the maximum reduction of 6% in dose delivery for 6MV photons with constant mMLC (4x4 cm) opening and varying secondary collimator field sizes from 4x4 cm to 10x10 cm is 6%. In case of fixed secondary collimator field size with variable field sizes of mMLC the maximum reduction in dose is 3%. The above two factors are due to backscattered radiation originating mainly from the secondary collimator with mMLC reaching the beam monitoring chamber. Also a maximum of 3% difference in backscattering is observed between mMLC and collimator. Hence we should take beam measurements very carefully while using detachable micro multileaf collimator because of the contribution of the radiation backscatter effect into beam monitor chambers.

### O-23

### Megavoltage cone beam CT imaging in IGRT: Verification of target and normal tissue location

Vikram Maiya, Kumara Swamy, Sumit Basu, Sathiyanarayanan, Shrikant Deshpande, Janhavi Bhangle, Nirmal Kumar Babu. *Department of Radiation Oncology, Ruby Hall Clinic, Pune, India*

**Introduction:** Verification of target and normal tissue location is crucial for 3D-conformal radiation therapy and IMRT because higher positioning uncertainty leads to larger planning target volume margin for tumor coverage, which in turn increases normal tissue complications and hinders dose escalation efforts. To improve treatment accuracy, megavoltage cone-beam computed tomography (MVCBCT) using an electronic portal-imaging device is used. MVCBCT scans acquired on the treatment machine provide three-dimensional (3D) anatomic information of normal tissues and tumors, overcoming a major limitation of the conventional setup approach that employs two-dimensional (2D) matching of the bony anatomy identified from portal images. In this study, we have used MVCBCT, for verification of target and normal tissue location.

**Materials and Methods:** 5 patients receiving IMRT for various malignancies were selected for this study. 2 of the patients had cancers of the head and neck, 1 had malignancy of the mediastinum, 1 had malignancy of the abdomen and 1 had malignancy of the pelvis. All the patients underwent MVCBCT using ONCOR Impression LINAC before treatment execution. All these patients underwent MVCBCT, wherein the gantry rotated from 270° to 110° using 8MU protocol. After image registration, the offset/shift was calculated in the ant-posterior, cranio-caudal and lateral directions. The shift was secured after it was compared with the planning CT scan. The necessary offset correction was applied.

**Results:** In all the patients, after co-registration of the planning CT images having structures with MVCBCT images, the offsets were obtained. All the offsets within 3 mm were not corrected and those situations with offsets greater than 3 mm, the necessary corrections were done using the table movements.

**Conclusion:** The present study shows that MVCBCT is a useful tool that is used in verification of target and normal tissue location. This paves the way for highly conformal radiotherapy at greater doses delivered with increased confidence and safety.

### O-24

### Normoxic pagat gels evaluated with optical CT scanner for verification of IMRT plans

Brindha S, Ebenezer Suman Babu S, BS Timothy Peace, Subhashini John, Paul B Ravindran. *Department of Radiation Oncology, Christian Medical College, Vellore - 632 004, India*

**Introduction:** Laser optical CT scanner has been established as a suitable evaluation tool for evaluating the PAGAT gel dosimeter. In this study the application of this normoxic gel for verification of three IMRT treatment plans has been performed.

This PDF is available for free download from a site hosted by Medknow Publications (www.medknow.com).

[Downloaded free from http://www.jmp.org.in on Friday, July 29, 2011, IP: 130.14.254.25] || Click here to download free Android application for t

**Materials and Methods:** The components used for the preparation of this gel was 5% gelatin, 3.5% BIS, 3.5% AA, 88% distilled water and 10 mM THP. The inverse planning for the selected cases was performed with the PLATO sunrise system and transferred to the CT scans of the gel phantom. The gel phantoms were irradiated with their long axis perpendicular to the central axis of the beam. The plane at which the laser beam of the optical scanner scans the gel phantom was measured as 4.7 cm from the base. The midpoint of this plane was positioned at isocentre for irradiation. The gel dosimeter was calibrated by irradiating the gels to 20 x 20 $mm^2$ fields up to a dose of 8Gy using 6 MV photons from the PRIMUS linear accelerator.
**Evaluation of the gel:** Pre and post irradiation scans of the phantom were performed to obtain the attenuation measurements during reconstruction. The reconstruction was carried out using the 'iradon' reconstruction algorithm of the MatLab with 'Hann and spline' filters. The dose distributions obtained were compared with those from the treatment planning system (TPS) using the Verisoft software from PTW, Netherlands. A quantitative comparison of measured and calculated was obtained using the gamma technique developed by Low *et al*, 1998. Since the aim of the present study was to investigate the feasibility of using the gel for verification of complex radiotherapy plans, the plan obtained from the treatment planning system was taken as the reference and the gamma indices were calculated. Each measured point was evaluated to determine if both the dose difference and DTA (distance to agreement) exceeded the selected tolerances 3% and 3 mm, respectively.
**Results and Discussion:** All the three cases showed a good comparison between the TPS and gel. The gamma distribution for the comparison of gel-TPS dose distributions passed the gamma criteria (3 mm spatial/3% dose deviation) in all regions except the low dose region and region near the container walls. The difference between the TPS and gel distribution has been found to be less than 2 mm for small tumors. The size of the gel container is the limiting factor since a deviation from the gamma criteria has been observed in the region near the container walls. This could be overcome by using larger gel phantoms. To obtain dose distribution, an absolute measurement of radiation dose calibration of each batch of gel is required. The time required to verify the dose distribution for an IMRT plan is 20 minutes including the time taken for post-irradiation scan.
**Conclusion:** Feasibility of using the PAGAT gel evaluated with the laser CT scanner for verification of IMRT treatment plans was investigated. Both the gamma comparison and distribution comparison suggests that an accurately calibrated PAGAT gel evaluated with laser CT can be used as a quality assurance tool for complex treatment plans with high dose gradients.

This PDF is available for free download from a site hosted by Medknow Publications (www.medknow.com).

### O-25
### Evaluation of image quality characteristics of integrated spect-CT

Rameshwar Prasad, B Shetye, V Rangarajan, AM Samuel. *Bio-Imaging Unit, TMH, Mumbai, India*
E-mail: rjhaji@yahoo.co.in

**Introduction:** Integrated SPECT-CT has emerged as a new facility in the recent past due to its unique ability of providing both functional and anatomical images in a single imaging session. In this work image quality characteristics of SPECT and CT of integrated SPECT-CT scanner were studied. The effect of different collimators and different radius of rotation on spatial resolution, uniformity, noise and contrast levels were measured for each SPECT image. For CT, uniformity, density, slice thickness, high contrast and low contrast were calculated. The SPECT and CT image registration was also quantified for fusion imaging.
**Materials and Methods:** For SPECT an acrylic cylindrical Jaszczak phantom, with insert of spheres measuring 9.5, 12.7, 15.9, 19.1, 25.4 and 31.0 mm diameter, insert of rods measuring 4.8, 6.4, 7.9, 9.5, 11.1 and 12.7 mm diameter and uniformity area was used. The phantom was filled with water and 15 mCi activity of Tc99m ($TcO_4^-$) was uniformly distributed. The images were acquired with a dual-head GE INFINIA HAWKEYE gamma camera having 1.523% and 1.536% integral uniformity, 1.072% and 0.85% differential uniformity in the CFOV for two detectors, 9.8% energy resolution and center of rotation deviation of 0.412 mm and 0.1 mm. The photo peak was set at 140 keV with a 20% centered energy window. For CT, 20 cm QA Phantom containing 3 sections, namely first section as slice alignment, spatial resolution, CT scale and slice thickness section second section as low contrast resolution section and third section as water section each corresponds to single scan plane. SPECT/CT registration phantom was used for quantification of SPECT-CT registration.
**Results:** The reconstructed spatial resolution for LEHR with 157 mm and 210 mm radius of rotation was 7.3 mm and 9.2 mm respectively whereas for LEGP it was 9.2 mm and 11.4 mm. All SPECT images were of uniform intensity. There was no significant variation among two collimators at smaller radius of rotation but significant variation of uniformity among LEHR and LEGP was obtained at larger ROR. Signal to noise ratio for 157 mm ROR were 0.206 and 0.201 for LEGPand LEHR but for 210 mm ROR these were 0.197 and 0.191 respectively. Contrast recovery for cold regions were 0.177 and 0.370 for LEGPand LEHR (for 157 mm ROR) but were 0.363 and 0.63 (for 210 mm ROR) respectively. Contrast recovery for Hot regions were 0.177 and 0.370 for LEGPand LEHR (for 157 mm ROR) but were 0.363 and 0.63 (for 210 mm ROR) respectively. CT Uniformity (mean) was 0.3. CT Density (mean) for air, plastic and water were 1.18, 1132, 996.89.CT slice thickness was between 9 to 10 mm. In CT high contrast line separations were 3.0 line pairs per cm. Number of visible bar sets were 3. For low contrast, number of visible holes were 7 and smallest visible hole size was 3 mm. The SPECT-CT image alignment was 1.87, 1.99 and 0.64 mm in X, Y and Z direction respectively. The image quality of this scanner is suitable for clinical imaging and quantifications.
**Conclusion:** The image quality characteristics of integrated SPECT and CT scanner should be evaluated before it is used for clinical imaging. The LEHR collimator can contribute effectively to a better resolution than the LEGP collimator. The image quality of SPECT-CT becomes inferior as detector goes away from source. The image registration must be calibrated for fusion imaging.

### O-26
### Software development for tumor volume measurement using SPECT system

NS Baghel, B Rajashekharrao*, S Dey**, RK Kher***, N Nair. *Radiation Medicine Centre (BARC), *Radiation Safety Systems Division (BARC), **Computer Division (BARC), ***Radiological Physics and Advisory Division (BARC), Tata Memorial Centre Annexe, Parel, Mumbai - 400 012, India*

**Introduction:** For estimation of tumor or tissue absorbed dose from radionuclide therapy procedures, determination of tumor or tissue volume is one of the most important radiation dosimetric parameter. Though, a variety of image segmentation techniques using SPECT have been proposed and described for tumor volume measurement, no software however is available for this purpose. The objective of the present work is development of windows based software for volume measurement and its evaluation and validation using known volume spherical glass phantoms.
**Materials and Methods:** A windows based software has been developed in visual C++ using Insight Tool Kit (ITK) library for 3D image segmentation and the Visualization Tool Kit (VTK) library for segmented image display. We have implemented three segmentation methods *viz.* manual fixed threshold, Adaptive threshold and optimal threshold. These three segmentation methods were inter-compared to determine the most appropriate technique for volume measurement. The basic approach of this software is to detect boundary of tumor/object using a segmentation method and count all the voxels inside the boundary and multiply with the volume of voxels for calculation of total volume. In fixed threshold method, lower threshold was applied and all the voxels having gray level above the threshold were counted as part of object. In adaptive threshold method, a grey level threshold was found out from gray level histogram of the object which was a boundary of background and object voxels. In optimal threshold method, segmentation threshold was selected through an iterative procedure to separate the voxels into object and non-object

[Downloaded free from http://www.jmp.org.in on Friday, July 29, 2011, IP: 130.14.254.25] || Click here to download free Android application for t

(background) voxels. Spherical glass phantoms of different volumes ranging from 10 to 1000 ml were used to collect data on Siemens e.cam SPECT system using various radioactivity concentrations of $^{99m}$Tc and $^{131}$I radioisotopes. To establish a relation between radioactivity concentration and exposure rate for different volume phantoms, surface exposure rate measurement on each phantom was carried out using a portable ionization chamber type of radiation survey meter. The data was acquired in 128x128 matrix and 64 steps in 360$^{o}$ with minimum radius of rotation (ROR) using STEP and SHOOT method with Low-energy high resolution and High-energy collimators respectively. The reconstruction was carried out using Fourier Back Projection (FBP) method using Butterworth filter. Scatter and attenuation correction methods were not applied to the data in the present work. Reconstructed transaxial data was transferred in DICOM format to a networked PC and analysed using the developed software for volume estimation.

**Results:** The Fixed threshold method is volume dependent and therefore requires prior knowledge of volume of object. To minimize the error in volume measurement, fixed threshold is higher in case of smaller volume than larger volume. Adaptive threshold method works well for larger volume and it over estimates for smaller volume. Optimal threshold method determines smaller and bigger volumes within acceptable limits of error. However, $^{99m}$Tc studies require attenuation correction on SPECT data. The percentage error in volume measurement was more for smaller volume phantoms than the larger ones for both the $^{99m}$Tc and $^{131}$I phantom studies.

**Conclusion:** The software developed is semi-automatic with minimum user intervention and requiring reconstructed data in DICOM format. The technique of volume measurement standardized would be used for radiation dosimetry studies in patients of thyroid cancer given $^{131}$I treatment.

### O-27
### Routine quality control of PET/CT scanner

Amit K Singh, GS Pant, A Malhotra. *Dept. of Nuclear Medicine, AIIMS, New Delhi, India*

**Introduction:** PET/CT is an emerging diagnostic imaging modality in nuclear medicine with its definitive role in oncology, neurology and cardiology. To have good quality images with reproducibility, routine quality control of the PET/CT scanner is recommended. The aim of this study was to perform routine quality control of the PET/CT scanner at our institution.

**Materials and Methods:** Daily quality control of our PET/CT scanner (Siemens, biograph LSO crystal) were performed with the CT phantom and PET 3D phantom. The chi square values and the values of percentage of detectors outside 3 standard deviation (SD) were obtained each day. Whenever the Chi square value exceeded 2.5, normalization of the PET detectors was done. The Chi square value was then checked after normalization. The image co-registration was also checked with the help of a locally fabricated phantom. The CT numbers were checked weekly after imaging the CT phantom.

**Results:** The Chi square value ranged between 0.8 and 2.5 for about a month after normalization. The normalization is done when the value of Chi square exceeded 2.5 or after a month whichever is earlier. The normalization is also done after any repair of the hardware or re-loading of the software. The co-registration was also found to be acceptable. The CT values for water were within 0 ±3.

**Conclusion:** To get the optimum image quality of PET/CT, it is necessary to daily check the chi square value for PET and weekly check of CT phantom images. The image quality was reproducible when chi square value were within the tolerance limit of <2.5. The co-registration of PET and CT images was also acceptable all the time.

### O-28
### 4π Gamma ion chamber: A secondary standard for the calibration of radio-nuclides

Nathuram R. *Radiation Safety Systems Division, Bhabha Atomic Research Centre, Mumbai - 400 085, India*

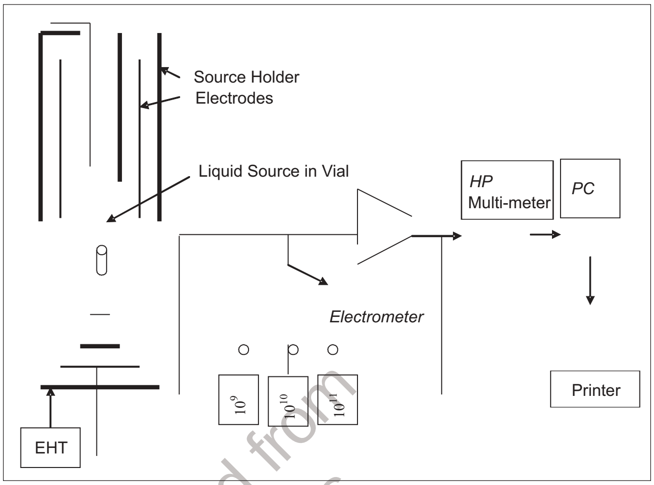

**Figure 1:** Block diagram of the gamma ion chamber set up

Table 1: Response factors of the 4π gamma ion chamber

| Radionuclide | Sensitivity factor (pA/mCi) | Overall uncertainty (%) |
|---|---|---|
| Cobalt-58 | 0.726 | 1.5 |
| Cobalt-60 | 1.7 | 1.0 |
| Cesium-134 | 1.205 | 1.5 |
| Cesium-137 | 0.441 | 1.6 |
| Iodine-131 | 0.324 | 1.6 |
| Mercury-203 | 0.22 | 1.2 |
| Iron-59 | 0.803 | 1.5 |
| Yittrium-88 | 1.758 | 1.5 |
| Europium -152 | 0.912 | 1.3 |

**Introduction:** During last three decades, 4π gamma ion chamber has remained the pre eminent method for standardization of radionuclides. Though other methods such as defined solid angle, coincidence and anticoincidence counting have been used for specific radionuclides, the 4π gamma ion chamber measurements cover sources decaying by electron capture and positron emission. For routine calibration of radionuclides, radiation standards section maintains a high-pressure 4π Gamma ion chamber as secondary standards. The Centronic 20$^{th}$ century IG 12 gamma ion chamber filled with argon at 2MPa has been recently calibrated with sources procured from Isotope Products Laboratories, Valecia having traceability to NIST, USA. Using the calibration factors of 4π gamma ion chamber thus obtained, radioactivity measurements are traceable to the national primary standards, which is also maintained at BARC. The calibration factors obtained as a function of average energy of several isotopes are presented in this paper.

**Materials and Methods:** Radioactive sources are widely used in several applications of nuclear sciences, medicine and engineering. In some of the applications, it is required to know radioactive strength accurately through measurements with suitable detector. However in measurements involving high accuracy, it is necessary to calibrate the detector with reference sources whose activity has been determined accurately. The RSS Division maintains secondry standards of radioactivity with the intention of providing and standardizing the radiation sources to the users of DAE and non DAE institution in the country. The secondary standard consists of Centronic IG12/A20, 4π or re-entrant high pressure ion chamber filled with argon gas at 20 atmospheric pressure connected to current measuring system. Circular lead rings about 5 cm thick shield the ion chamber. The current produced in the ion chamber is measured using an electrometer and the electrometer output is connected to a six and half digit Hewlett Packard

This PDF is available for free download from a site hosted by Medknow Publications (www.medknow.com).

[Downloaded free from http://www.jmp.org.in on Friday, July 29, 2011, IP: 130.14.254.25] || Click here to download free Android application for t multimeter. The multimeter is coupled to a computer using RS232 interface. Interactive program has been developed to read data at fixed time intervals, compute the average, correct it for background and store it on the hard disk for later processing and calculations. The block diagram of the secondary standard system is given in Figure 1. This system is calibrated against the primary standard, the $4\pi\beta$-$\gamma$ coincidence setup for beta, gamma emitting radionuclides. Two $^{226}$Ra sources of 10 $\mu$Ci, 100 $\mu$Ci encapsulated in stainless steel are used as reference sources, to check for the long term stability of the system. In order to establish the traceability of $4\pi$ gamma ion chamber to BIPM and other international laboratories, a set of liquid sources of $^{58}$Co, $^{60}$Co, $^{59}$Fe, $^{137}$Cs, $^{134}$Cs, $^{88}$y, $^{131}$I, $^{152}$Eu and $^{203}$Hg in glass vials having traceability to NIST, USA, was procured from Isotope Products Laboratories, Valecia (Schrader 2000). Each source in turn was placed in the chamber and current produced was observed for a set of twenty readings. Large number of measurements were taken to improve the statistical uncertainty. Each current measurement, which is corrected for background, is an average of 20 observations taken within a time period of 100 seconds. This current is denoted as the source current *Is*. Activity of the source is calculated using the following formula:

$$A = \frac{Is}{S} \times \frac{Iraref}{Iraobs}$$

*A* = Activity in Mbq
*Is* = Average current of the source in ampere
*S* = Sensitivity factor for the particular radionuclide in PA/mCi
*Iraref* = $^{226}$ra current at the time *S* was determined
*Iraobs* = $^{226}$ra current at source measurement time

The ionization chamber has initially been calibrated after absolute standardization of several radionuclides like $^{60}$Co, $^{59}$Fe, $^{54}$Mn, $^{137}$Cs, $^{134}$Cs, $^{22}$Na, $^{57}$Co and $^{133}$Ba on national primary standards. These calibrations have been compared by measurements of an ampoule sent by BARC to the International Reference System (SIR) of the BIPM (Rytz, 1983). Results and Discussion: The results in the form of sensitivity factors of $4\pi$ gamma ion chamber measured for a set of sources of average energy in the range of 124.6 keV to 1.25 MeV are given in Table 1. Also shown against each isotopes, are the sensitivity factors of 4 gamma ion chamber obtained with sources standardised on primary standards at BARC, Mumbai. The relative deviations in the response factors measured for sources standardized at BARC and NIST are within ±5% and is attributed to relative uncertainties in the standardistion. and is well within range of experiental measurements. Accuracy: Most of radioactive solutions having activity in the range of 10 kBq/g to 100 MBq/g, can be standardized by above technique with uncertainty of measurement in the range of ± 0.2% to ± 1% depending on the radionuclide.

### References

1. Rytz A. The international reference system for activity measurements of $\gamma$-ray emitting nuclides. Int J Appl Radiat Isotopes 1983;34:1047.
2. Schrader H. Calibration and consistency of results of an ionization chamber secondary measuring system for activity. Int J. Appl Radiat Isotopes 2000;52:325.

### O-29
### Dose distribution calculation of $^{125}$I source in water phantom using MCNP4C code

Ali Asghar Mowlavi, Edalat Mokhtari Njad*, Alireza Binesh**, Reza Izadi-Najafabadi***. *Physics Department, School of Sciences, Tarbiat Moallem University of Sabzevar, P.O. Box 397, Sabzevar, *Physics Department, Payam Nour University of Fariman, Fariman, **Physics Department, Payam Nour University of Mashhad, Mashhad, ***Physics Department, School of Sciences, Ferdowsi University of Mashhad, Mashhad, Iran*
Corresponding Author: Dr. A. A. Mowlavi,
E-mail: amowlavi@sttu.ac.ir

**Introduction:** Monte Carlo calculation of dose distribution in water phantom due to a $^{125}$I source.
**Materials and Methods:** The dose distribution has been calculated around the model 6711 $^{125}$I source located in the center of 30 cm × 30 cm × 30 cm water phantom cube by using MCNP4C code.
**Results:** The percentage depth dose (PDD) variation along the different axis parallel and perpendicular to the source are calculated. Then, the isodose points were found by interpolation from the relative dose curves. Finally, the isodose curves for 125%, 100%, 75%, 50% and 25% PDD are presented.
**Conclusion:** Dose deposition in high gradient region, near the source, can only be calculated accurately by Monte Carlo method. The results can be used in treatment planning systems and also for computation of model dependent parameters.

### O-30
### Changes in applicator position with fractionated high dose rate gynecological brachytherapy

Rahul B Umbarkar, SS Supe, KK Singh, RS Shinde*, Mukund Sarje, Vandana Jain. *Pravara Rural Hospital, Kidwai memorial Hospital, Bangalore, *PVP College, Loni, India*

**Introduction:** Pravara Hospital has installed a high dose rate after loading (HDR) for gynecological brachytherapy in 1997. The number of fractions delivered varies from center to center, but at Pravara three fractions of 7Gy each at weeks interval are delivered. One of the major advantages claimed for high dose rate insertions is the opportunity to gain a better physical dose distribution by careful positioning and better packing for the short duration of treatment is required. Since only small changes in distance between applicator source and important anatomical structures such as bladder and rectum may result in large changes in dose delivered to these sites. Critics of HDR brachytherapy cite that patients should never be moved with an applicator *in situ* because of possible applicator movement and the potential for uterine perforation. So the purpose of the study was to evaluate changes in applicator position in-patients receiving high dose rate gynaecological brachytherapy as part of combined external beam and brachytherapy treatment for cervical cancer.
**Materials and Methods:** 30 patients have been studied. All were receiving radical radiotherapy for their disease. All the patients had combined external beam and intracavity treatment. All intracavity treatments were given using a Gammamed (Isotopen- technik Dr. Sauer-wein Gmbh, Germany) high dose rate after loading machine with an iridium ($^{192}$ Ir.) source of nominal activity of 10 curies. After completion of external beam radiation of 50Gy, patient was called after one week's time. For all insertions each patient had general anesthesia at which examination under anesthesia was performed followed by dilation of

Table 1: Details of measurements taken from AP and Lat. X-rays.

| | |
|---|---|
| Anteroposterior film | |
| Angle between the intrauterine tube and a line drawn across the inferior edge of the sacro-iliac joints. | |
| Lateral Films | |
| a. | *Pelvic position* |
| | Angle between lines drawn along the anterior body L5 and the anterior body of S1. Distance from sacral promontory to distal tip of intrauterine tube, minimum distance from ICRU bladder point to sacral hollow. |
| b. | *Applicator position relative to bladder and rectum* |
| | Minimum distance from ICRU bladder point to ovoid source, Distance from ICRU bladder point to ICRU rectal point on anterior rectal wall, Angle between a line joining ICRU bladder point to ovoid source and the straight axis of the applicators. |

This PDF is available for free download from a site hosted by Medknow Publications (www.medknow.com)

[Downloaded free from http://www.jmp.org.in on Friday, July 29, 2011, IP: 130.14.254.25] || Click here to download free Android application for t

**Table 2: Details of individual parameters for bladder, rectum and ovoid source positions; Difference for each parameter between all three insertions**

| Patient | Ovoid/bladder angle (degrees) | Bladder/ovoid distance. (mm) | Bladder/rectum distance. (mm) |
|---|---|---|---|
| 1. | 15 | 6 | 3 |
| 2. | 25 | 1 | 8 |
| 3. | 23 | 3 | 13 |
| 4. | 20 | 3 | 12 |
| 5. | 7 | 1 | 9 |
| 6. | 20 | 2 | 5 |
| 7. | 80 | 3 | 8 |
| 8. | 70 | 2 | 7 |
| 9. | 30 | 5 | 5 |
| 10. | 5 | 2 | 12 |
| 11. | 15 | 2 | 19 |
| 12. | 25 | 5 | 5 |
| 13. | 23 | 6 | 1 |
| 14. | 20 | 2 | 0 |
| 15. | 60 | 3 | 14 |
| 16. | 10 | 1 | 12 |
| 17. | 40 | 5 | 22 |
| 18. | 45 | 2 | 6 |
| 19. | 30 | 3 | 8 |
| 20. | 25 | 4 | 9 |
| 21. | 15 | 2 | 2 |
| 22. | 20 | 6 | 5 |
| 23. | 20 | 5 | 8 |
| 24. | 15 | 2 | 3 |
| 25. | 12 | 0.5 | 8 |
| 26. | 0.5 | 6 | 2 |
| 27. | 15 | 1.2 | 1 |
| 28. | 13 | 5 | 0.7 |
| 29. | 9 | 7 | 5 |
| 30. | 10 | 1 | 2 |
| Median | 35.0 | 2.0 | 13.0 |

the cervix. This was then used to locate a standard fixed geometry intrauterine tube (tandem) and ovoid, three-channel applicator system based on Fletcher style dosimetry. For each insertion standard antero-posterior and lateral radiographs are taken with magnification marker wires in the applicators. As per the ICRU 38 recommendations the bladder was localized using Hypaque within a catheter balloon and the rectum using rectal wire. The AP and lateral films have been analyzed by taking measurements on the films to indicate reproducibility of pelvic position, applicator position and relation to the ICRU bladder dosimetry point and the anterior rectal wall. The measurements are shown in Table 1. Results: For all 30 patients it was possible to compare the measurements in Table 2 for all three insertions. The pelvic positions were measured by the angle between L5 and the sacrum reflecting pelvic tilt and the distance between the ICRU bladder point and the sacral hollow. Between all the three insertions the L5 sacral angle varied by a mean 5° and at present it is minimal of 1° (range, 0-10) and the ICRU bladder distance to sacral hollow varied by a mean of 8mm (range, 2-29 mm). There was a change in distance from sacral promontory to tube tip of 8.5 mm (range, 0-18 mm). The tube position on the AP projection varied by a mean of 9° (range, 0-17°) with an overall of 93° (range, 83-112°). One important result emerges in direct relation to the ovoid position as measured by the angle between a line joining the ICRU bladder dose point to the ovoid source and the straight axis of the applicators. This is shown in Table 2 where a median change of 35° (range, 5-80°) is seen reflecting considerable craniocaudal movement between all three-insertion in-patients. The relation between the ICRU bladder dose point and the applicators is reflected in the distance between ICRU bladder point and the ovoid source. As mentioned above there is marked movement in the craniocaudal position of the ovoid source between insertions and the distance in the antero-posterior plane varied by a median of only 2-mm (range, 0-10 mm) The dose to the anterior rectal wall is reflected in the measurement between the ICRU bladder point and ICRU rectal point on the anterior rectal perpendicular to the bladder. The variation in this measurement is shown in Table 2 where median of 13 mm between all the insertions is seen (range, 0-22).

**Conclusion:** The results of this study have shown that there was variation in applicator position on repeated insertions in the same patients using high dose after loading techniques. In particular there is marked craniocaudal movement of the applicators. This is possibly reflects different fixation of the applicators between all the insertions. The importance should be given to individual planning for each high dose rate insertion where a fractionated treatment is required in order to accurately define the dose to the tumor area and more critically to limiting normal tissue structure within treatment area.

### References

1. Hoskin PJ, Cook M, Bouscale D, Cansdale J. Changes in applicator position with fractionated high dose rate gynaecological brachytherapy. Radiother Oncol 1996;40:59-62.
2. ICRU report no.38. Dose and Volume specification for reporting intracavitary therapy in gynecology, International commission on radiation units and measurements. Bethesda, MD; 1985.
3. Orton CG. Remote after loading for cervix cancer: The physicists point of view, in Brachytherapy HDR and LDR. Martinez AA orton CG, Mould RF (editors). Nuclerton: Coulombia USA; 1990.

**O-31**

**Evaluation of Air-Kerma strength of HDR $^{192}Ir$ brachytherapy source using ion chambers and monte carlo simulation**

K Devan, D Manigandan, G Bharanidharan, Hema Vaidyanathan*, KV Subbaiah#, P Aruna, S Ganesan. *Division of Medical Physics and Lasers, Department of Physics, Anna University, Chennai - 600 025, Kidwai Memorial Institute of Oncology, Bangalore - 560029, #Safety Research Institute, Indira Gandhi Center for Atomic Research, Department of Atomic Energy, Kalpakkam - 603 102, India*
E-mail: kdevn@yahoo.co.in

**Introduction:** A high dose rate $^{192}Ir$ source is commonly used in brachytherapy. The recommended quantity for specifying the strength of brachytherapy sources is air kerma strength. Air-kerma strength is specified in terms of air-kerma rate at the point along the transverse axis of the source in free space. Primarily in-air calibration of the source is performed by the standardization laboratories such as National Institute of Standards and Technology (NIST), Accredited Dosimetry Calibration Laboratories (ADCL) in USA and the National Research Council of Canada (NRCC). During purchase and installation the manufacturer issues a calibration certificate of source strength with an overall uncertainty of ± 5% along with other specifications of the source. The user has to cross check practically to verify the accuracy of source strength provided by the vendor. Typically, the user has a well-type ionization chamber that has a calibration traceable to the national standards for each type of brachytherapy source. The objective of this study is to evaluate and compare the air-kerma strength of the HDR source using therapy ion chambers and Monte Carlo method. It is also aimed to study the effect of applicator on the value of air-kerma strength during measurements.

**Materials and Methods:** The GammaMed Plus HDR $^{192}Ir$ source from MDS-Nordion Haan GmbH, Germany (Now Varian Medical System) was calibrated. The activity of the source was 5.917 Ci. The in-air calibrations of the HDR $^{192}Ir$ source was performed using, i) 0.6 CC Farmer ion therapy ionization chamber (UNIDOS dosimeter from PTW-Freiburg, Germany) and ii) the well-type ionization chamber HDR-1000 (Standard Imaging Inc, Middleton, Wisconsin). Computation of dose rate was done using the Monte Carlo N-Particle transport code System (MCNP) version 4B computer program. MCNP4B is a general-purpose code for calculating the time-dependant, continuous-energy transport

This PDF is available for free download from a site hosted by Medknow Publications (www.medknow.com).

[Downloaded free from http://www.jmp.org.in on Friday, July 29, 2011, IP: 130.14.254.25] || Click here to download free Android application for t of neutrons, photons and/or electrons in three-dimensional geometry. Point detectors (F5 tally) and ring detectors (F5a tally) were used to compute flux at various points of interest, where 'a' can be either X or Y or Z-axis, the coordinates of the detector location. The decay photon energy spectrum of $^{192}$Ir consists of 31 lines with energy ranging from 8.91 keV to 1.061 MeV. Total photon yield is 2.363 photons per decay. Each photon history originates at a random position and direction inside the source core. The simulations were done for $10^6$ photon histories to obtain statistically acceptable results. Each photon history was traced down to 1keV, a default cutoff energy set by MCNP.

**Results and Discussion:** Initially the responses of the chambers were studied. The measurements were carried out placing the source inside a stainless needle as a source applicator. The inner and outer diameter of applicator was 1.35 mm and 1.65 mm respectively, with a total length of 200 mm. The evaluated air-kerma strength of the GammaMed plus HDR $^{192}$Ir source was found to be 23.31 ± 5% mGym$^2$h$^{-1}$ and 24.27 ± 5% mGym$^2$h$^{-1}$ with Farmer ion chamber and Well Type chamber respectively, against the manufacturer stated value of 24.2 ± 5% mGym$^2$h$^{-1}$. For Monte Carlo simulation, the source was designed with applicator and without applicator. The applicator was designed in order to study effect of the applicator on the value of air-kerma strength of the source. The Monte Carlo (MCNP4B) computed air-kerma strength of the source was 24.5 ± 2% mGym$^2$h$^{-1}$ and 24.6 ± 2% mGym$^2$h$^{-1}$ with the applicator and without the applicator, respectively. The percentage differences are 3.7%, -0.29%, -1.24% and -1.65% for Farmer ion chamber, Well Type chamber, Monte Carlo simulated (with applicator) and Monte Carlo simulation (without applicator) respectively, when compared against the quoted air-kerma strength value of the manufacturer. The percentage difference between the value of air-kerma strength of the source with and without applicator is 0.4%. This shows that, the effect of applicator material on the air-kerma strength is negligible. Hence, it can thus be ignored during practical measurements.

### References

1. Nath R anderson LL, Luxton G, Weaver KA, Williamson JF, Meigooni AS. Dosimetry of interstitial brachytherapy sources: Recommendations of the AAPM Radiation Therapy Committee Task Group No.43. Med Phys 1995;22:209-34.
2. MCNP - A General Monte Carlo N-Particle Transport Code, Version 4B, Transport Methods Group. Los Alamos National Laboratory report; 1997.
3. Hubbell JH, Seltzer SM. Tables of X-ray mass attenuation coefficients and mass energy-absorption coefficients 1 keV to 20 MeV for elements z=1 to 92 and 48 additional substances of dosimetric interest. NISTIR: 1995. p. 5632.

This PDF is available for free download from a site hosted by Medknow Publications (www.medknow.com).

### O-32

### Dosimetric comparison of different optimization techniques on CT based brachytherapy treatment planning of soft tissue sarcoma using dose volume indices

Pramod K Sharma, Dayananda S Shamurailatpam, S Laskar*, SV Jamema, RA Kinhikar, DD Deshpande. *Department of Medical Physics, *Radiation Oncology, Tata Memorial Hospital, Mumbai, India*

**Introduction:** Interstitial brachytherapy (BT) is one of the preferred modalities for the management of soft tissue sarcoma (STS). Traditional method of implant dosimetry using a set of radiographs fails to correlate the dosimetric outcome on patient anatomy and lack of qualitative information. CT based dosimetry provides the dose distribution on the patient images and enable to quantify the dosimetric outcome using dose volume indices. The aim of this study is to compare the impact of different optimization algorithm on the dosimetric outcome using different dose volume indices derived from the dose volume histogram.

**Materials and Methods:** Intraoperative placement of flexible nylon tubes were performed on ten patients of STS in various sites of extremities and chest wall. All patients underwent CT scan of 5 mm slice thickness with 0.2 mm copper dummies inserted in the tubes. Target volume delineation and reconstruction of the implanted tubes were carried out from the CT datasheet using Plato-Sunrise treatment planning system. Dwell position in each catheter was loaded by giving an extra margin of 5 to 8 mm across the target volume. Four plans were generated for each patient. Basel dose points were defined i) at a single transverse plane and middle of the implanted volume and ii) throughout the implanted volume. Dose were normalized and optimized geometrically in both the cases and yielded plan 1 (P1) and plan 2 (P2) respectively. Another two plans were generated with dose prescription points defined along the catheter and in a plane 5 mm above or below the implanted plane. Dose was normalized and optimized on these points and this yielded Plan 3 (P3). Plan 4 (P4) was generated by employing graphical optimization on P3. Dosimetric outcome from these 4 different plans were compared qualitatively and quantitatively using the dose volume indices which include coverage index (CI), relative dose homogeneity index (DHI), external volume index (EI) and overdose volume index (OI). CI is the fraction of the target receiving a dose equal to or greater than the reference dose, DHI is the fraction of target receiving a dose between 100 and 150% of the reference dose, EI is the ratio of the normal tissue volume outside the target receiving a dose equal to or greater than the reference dose to target and OI is the fraction of the target receiving a dose equal or greater than two times the reference dose. A total dose of 36 Gy in 9 fractions were delivered twice a day.

**Results:** Single plane implant with catheter ranging from 8 to 14 was used in all patients. The volume of the target ranges from 50.8 to 219 cc (Mean 126.55 cc, SD 50.69). Qualitative evaluation of the dosimetric outcome on the CT slices from P1, P3 and P4 shows reasonably good coverage of target in all patients. Suboptimal target coverage was observed in P2. Graphical optimization yields the best target coverage at the cost of high dose volume. The median (SD) CI of target estimated from P1, P2, P3 and P4 was 0.76 (0.19), 0.64 (0.11), 0.83 (0.13) and 0.93 (0.03) respectively. The percentage increase in the median CI of CTV, 17.8% (w.r.t P1), 30.8% (w.r.t.P2) and 10.3% (w.r.t.P3) observed in graphically optimized plan (P3) lead to decrease in DHI from 0.73 in P2 to 0.64. In all patients V150 and V200 was lesser than 45 cc and 18 cc with mean of 39.36 cc (max) and 16.24 cc (max) respectively; highest being observed in P4. Highest median OI of 0.14 (SD 0.41) was observed in P4 as compared to 0.07 of P2, 0.11of P3 and 0.12 of P1. EI was highest in P1 with median 0.27 as compared to 0.06 of P2, 0.13 of P1 and 0.16 of P3.

**Conclusion:** Dose prescription and optimization on points define along the catheter and in a plane 5 mm above or below the implanted plane resulted optimal dosimetric outcome. Interactive graphical optimization still improves target coverage at the cost of high dose volume and dose inhomogeneity. Use of dose volume indices allow quantitative evaluation of different plans and can be use as a tool to correlate dosimetry with clinical outcome.

### O-33

### Dosimetric studies of a newly developed P-32 patch for the treatment of superficial tumours

Pankaj Tandon, SK Saxena*. *Radiological Physics and Advisory Division, CT and CRS Building, Anushaktinagar, Mumbai - 400 094, *Radiopharmaceuticals Division, BARC, Trombay, Mumbai - 400 085, India*

**Introduction:** Dosimetry plays a very important role while planning the treatment of tumours using radionuclides. Presently, there are many radionuclides being used in brachytherapy for the treatment of interstitial, intraluminal and intracavitary tumours. These superficial tumours are generally treated by chemotherapy, immunotherapy, surgical excision, laser therapy and radiation therapy using X-rays, by gamma radiation or electron sources. Each mode of treatment has its own advantages and limitations. A localized external source of beta radiation would deliver uniform dose to the affected area in a short time span with minimal damage to the normal tissues and hence would obviate several sources of inconvenience. Recently there have been reports on the use of $^{166}$Ho and $^{90}$Y incorporated skin patches for skin

[Downloaded free from http://www.jmp.org.in on Friday, July 29, 2011, IP: 130.14.254.25] || Click here to download free Android application for t tumour treatment.[1-5] In this paper, the dosimetric measurements were carried out on a newly developed skin patch using $^{32}P$ radionuclide for its potential use in superficial tumours. This patch contains about 37 - 185 MBq of activity, uniformly distributed on an area of 1.5-2.5 cm diameter sheets and can be easily applied on the affected lesion.

**Methods and Results:** $^{32}P$ patch was prepared in Radiopharmaceuticals Division of Bhabha Atomic Research Centre, by adsorption of liquid $^{32}P$ on 1.5-2.5 cm diameter sheets of cellulose based absorber paper strips. Known quantity of $^{32}P$ activity in dilute phosphoric acid medium with a concentration ranging from 1.66-2.75 GBq/ml was dispensed at the centre of strip and was subsequently allowed to dry in air at room temperature. The dried strip was encapsulated within 40 microns thick plastic sheet. Sources of various strengths varying from 37 MBq to a maximum of 185 MBq each were prepared and activity level of ~ 37 MBq/cm$^2$ of the paper strip could be achieved in final source form. The source was tested for non-leakage of radioactivity prior to dosimetric studies. The dosimetric measurements for this patch was carried out in a specially designed phantom made of poly methyl methacrylate (PMMA) sheet used as a substitute of tissue having a density of 1.19 gm/cc with an effective atomic number 6.48 and an aluminum sheets as a substitute of bone having a density of 2.70 and atomic number 13. In this phantom model, the planar source of radionuclide $^{32}P$ was sandwiched between the central portion of the slabs made up PMMA and aluminum sheets of thickness 1 mm each as shown in Figure 1. The dosimtery media chosen for performing this dose measurement was GAFChromic film having a 7 micron radiation sensitive layer on a 0.1 mm polyester base. The films were placed on either side of the source at different depths and exposed for pre-calculated time. After the stipulated time, the films were removed. There is a variation in the colour of the exposed film at different depths because of the penetrating power of the radiation and the density of the medium. The dose received by the exposed films was recorded using a film densitometer, 24 hours after irradiation. The Optical Density (OD) of unexposed films, used as control, was also recorded. The Net Mean Optical Density (NMOD) of each of the exposed film was calculated. The method of estimation of doses from the NMOD values is carried out using an empirical relation "A". This empirical relation was obtained by the calibration of the films independently using a $^{60}Co$ radioactive source.

D (Gy) = (NMOD - 0.035) x 10$^2$ ....... (A)
4.72

The results obtained are given in Table 1. From the results, it is noted that the dose fall is very rapid towards the tissue as well as towards bone. The dose rate at 4 mm in tissue is nearly the same as at 2 mm in bone. There is a 79% fall in the dose rate at 1 mm and 93% at 2 mm with respect to the contact dose.

**Conclusion:** The dosimtery of newly developed $^{32}P$ patch source could be done with the help of GAFChromic films. The dose rate obtained from the above designed experiment will be helpful to the clinician in deciding the time of treatment and the dose to be delivered, if the activity in the patch supplied is known. From the dosimetry results obtained, it can be concluded that these patches can be used for the treatment effectively. The sources can thus be evaluated further for their efficacy in animal models and could be developed as a method of choice for treatment of superficial tumours.

Table 1: Dose rate in mGyMBq$^{-1}$hr$^{-1}$

| Distance of film from source (mm) | Dose in (mGy/MBq/hr) in tissue | Dose in (mGy/MBq/hr) in bone |
|---|---|---|
| In contact | 272.8 | 272.8 |
| 1 | 56.9 | 20.4 |
| 2 | 20.4 | 1.4 |
| 3 | 7.2 | 0.1 |
| 4 | 1.5 | 0.1 |

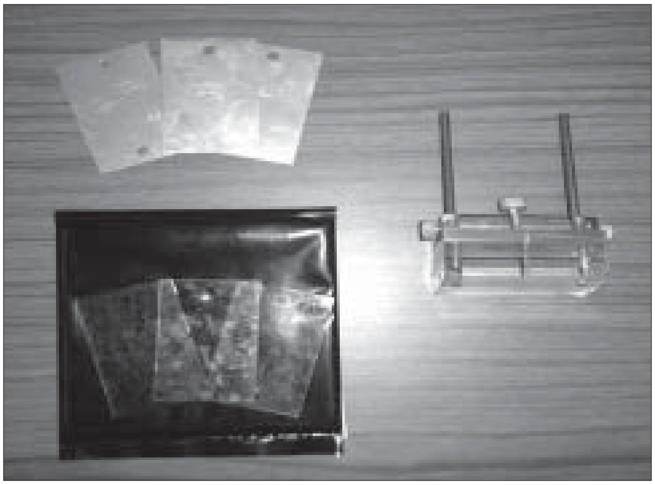

**Figure 1: Experimental set up used for measurement**

### References

1. Lee JD, Park KK, Lee MG, Kim EH, Rhim KJ, Lee JT, *et al.* Radionuclide therapy of skin cancers and Bowens disease using a specially designed skin patch. J Nucl Med 1997;38:697-702.
2. Park KB, Young MK, Ryu JM. Study on the preparation of holmium-166 patch for skin cancer treatment. International conference on future nuclear system. Wyoming: USA; 28 August to 3 September, 1997.
3. Chung YL, Lee JD, Bang D, Lee JB, Park KB, Lee MG. Treatment of bowens disease with a specially designed radioactive skin patch. Eur J Nucl Med 2000;27:842-6.
4. Jeong JM, Lee YJ, Kim EH. Simple preparation of beta ray emitting paper for treatment of skin cancer. J Nucl Med 1998;39:234P.
5. Mukherjee A, Pandey U, Sharma HD, Pillai MR, Venkatesh M. Preparation and evaluation of $^{90}Y$ patches for therapy of superficial tumours in mice. Nucl Med Comm 2002;23:243-7.

This PDF is available for free download from a site hosted by Medknow Publications (www.medknow.com).

### O-34

### Experience with brachytherapy ring applicator at CNCI Kolkata

Pradip Kumar, DK Ray, R Das, D Lahiri, P Bhagat, P Chaudhuri, T Maji. *Department of Radiotherapy, Chittaranjan National Cancer Institute, 37, S P Mukherjee Road, Kolkata - 700 026, India*
E-mail: prapin2003@yahoo.co.in

**Introduction:** Intracavitary brachytherapy plays an important role in the management of cervical cancer, which is one of the common cancers among Indian female population. We have used intracavitary ring applicator in 37 patients. Dosimetry of ring applicator was carried out

Table 1: Estimation of bladder and rectal dose in ICRT. Ring size: - 60 degree 26 mm, tandem size 60 degree 60 mm

| Sr. No. | % bladder dose | % rectal dose | | |
|---|---|---|---|---|
| | | I point | II point | III point |
| 1 | 74 | 43 | 56 | 51 |
| 2 | 72 | 24 | 55 | 33 |
| 3 | 68 | 62 | 69 | 29 |
| 4 | 58 | 45 | 68 | 27 |
| 5 | 56 | 56 | 67 | 51 |
| 6 | 32 | 48 | 57 | 45 |
| 7 | 33 | 41 | 49 | 34 |
| 8 | 64 | 37 | 40 | 35 |
| 9 | 75 | 35 | 52 | 21 |
| 10 | 69 | 35 | 46 | 40 |
| 11 | 51 | 33 | 44 | 43 |
| 12 | 77 | 29 | 37 | 33 |

Mean bladder dose (%): 60.75, Mean rectal dose (%): I point- 40.67, II point-53.33 and III point-36.83

[Downloaded free from http://www.jmp.org.in on Friday, July 29, 2011, IP: 130.14.254.25] || Click here to download free Android application for t

Table 2: Estimation of bladder and rectal dose in ICRT. Ring size: - 60 degree 30 mm, Tandem size 60 degree 60 mm

| Sr. No. | % bladder dose | % rectal dose | | |
|---|---|---|---|---|
| | | I point | II point | III point |
| 1 | 74 | 36 | 42 | 33 |
| 2 | 53 | 47 | 53 | 31 |
| 3 | 70 | 50 | 78 | 49 |
| 4 | 74 | 38 | 51 | 39 |
| 5 | 68 | 55 | 78 | 66 |
| 6 | 71 | 62 | 67 | 62 |
| 7 | 79 | 31 | 55 | 44 |
| 8 | 78 | 28 | 56 | 35 |
| 9 | 67 | 47 | 68 | 59 |
| 10 | 74 | 32 | 71 | 47 |
| 11 | 60 | 33 | 44 | 33 |
| 12 | 42 | 44 | 45 | 33 |
| 13 | 71 | 50 | 60 | 39 |
| 14 | 54 | 35 | 67 | 67 |
| 15 | 58 | 37 | 47 | 37 |
| 16 | 63 | 71 | 62 | 55 |
| 17 | 52 | 63 | 78 | 46 |
| 18 | 57 | 51 | 61 | 28 |
| 19 | 69 | 46 | 52 | 28 |
| 20 | 73 | 46 | 55 | 41 |
| 21 | 49 | 32 | 40 | 39 |
| 22 | 61 | 32 | 39 | 35 |
| 23 | 61 | 28 | 36 | 29 |
| 24 | 50 | 33 | 44 | 42 |
| 25 | 56 | 29 | 49 | 28 |

Mean bladder dose (%): 63.36, Mean rectal dose (%): I point- 42.24, II point-55.92 and III point-41.8

following the ICRU 38 specification. In this study 37 intracavitary ring applications were analyzed and the dose received by the critical structures like the bladder and rectum have been reported.

**Materials and Methods:** Patients with stage IIB, IIIA and IIIB cervical cancers who had been treated with external beam radiotherapy [50Gy/ 25 # /5Wks] followed by HDR Brachytherapy [21Gy/3#/3Wks] were studied. After insertions of ring applicator, patients were simulated and orthogonal radiographs were obtained. Rectal retractor was used for every patient. Manchester system of dosimetry was used for the computation of dose to point A and point B. The bladder was located using a Foley's catheter with 7cc contrast material and the bladder reference point was taken as per ICRU-38 specification. The rectum was located by using rectal marker considering several rectal reference points. Treatment planning was carried out on 3D PLATO Brachytherapy software. The dose prescribed for each fraction was 7Gy to point A and treatment delivered using MicroSelectron HDR (Nucletron) machine. Three ring diameters are available 26 mm, 30 mm and 34 mm, each has a 3 mm thick cap which is used wherever possible to increase the distance from the source train to the mucosa. Three tandem lengths are available 20 mm, 40 mm and 60 mm.The above rings and tandem are available with angulations of 30 degree, 45 degree and 60 degree. The ring system was chosen for two reasons; firstly by turning it on edge one can insert it more easily through the introitus than in the case for colpostats and secondly since it clamps to the tandem, the geometry of the applicator is fixed.

**Results:** The maximum and mean dose to the rectum and bladder reference points for two sets of applicator are shown in Tables 1 and 2. The maximum dose to rectum at point II was 78% and mean dose was 55.08%. Similarly for bladder maximum and mean dose were 79% and 62.51% respectively. The source loading was carried out diametrically opposite pair in ring, which is having more flexibility of source loading as per spread of disease and provides an added advantage. We generally do not do any optimization but by adjusting dwell position and dwell time weightage manually or graphically, isodose distribution is modified. Among all the ring applicator most frequently used applicator size was Ring 60 degree 30 mm Tandem 60 degree 60 mm followed by Ring 60 degree 26 mm Tandem 60 degree 60 mm, ring 60 degree 30 mm tandem 60 degree 40 mm. Also it was observed that proper gauze packing played very important role in further reduction of dose to bladder and rectum.

### References

1. ICRU Report-38: Dose and volume specification for reporting intracavitary therapy in gynecology.
2. High Dose Rate Brachytherapy: A Text Book, By Dr Subir Nag.

### O-35
### Treatment planning based on dose line optimization of a ultrasound directed breast implant

Preeti Deka, Raghavendra Holla, TR Vivek, Sanjiv Sharma. *Manipal Hospital, Bangalore, Karnataka, India*

**Introduction:** Breast conservative therapy is currently considered as a viable alternative to mastectomy in early breast cancer. Radiotherapy by virtue of its ability to reduce local recurrence is an integral of breast conservative therapy. Apart from irradiating the whole breast, the tumour bed is usually delivered a boost dose in breast conservative therapy to increase the local control rate. One of the methods which have been adopted to selectively boost the tumour bed to high doses is the use of HDR interstitial implants. This particular paper deals with our department's experience with breast conservative therapy of ultrasound directed HDR interstitial implant in early breast cancer using Brachyvision 6.5 Treatment Planning System.

**Materials and Methods:** Ultrasonography is done to locate the cavity left by lumpectomy. The cavity is marked on the skin of the patient's breast, depth and dimension of the cavity from skin surface is noted. Numbers of planes can be used according to requirement. Later on needles are replaced by nylon tubes. Interneedle distance is kept as 1.5 cm and interplane needle is 1.2 cm. The whole implant procedure is done under general anaesthesia. Patient is shifted to CT scan for imaging; 3 mm CT slices are taken. The images are transferred to TPS and body is contoured after 3D reconstruction. Tubes are digitized by using 3-Dimensional saggital and coronal images with respect to each other. After digitization, tubes are loaded with HDR Ir- 192 source by leaving about 1 cm margin from each end to avoid high skin dose. The source positions in the tube can be changed according to the clinical requirement. During planning, Geometrical Optimization allows conformal dose to the target and reduces dose to the surroundings and helps produce a homogeneous dose distribution in a volume implant. Dose line Optimization implemented in Brachyvision 6.5 is designed to account for critical structures. One of the features helps the planner to alter dose values by pushing an Isodose line towards or away from a structure boundary. This real time process may be accomplished in any view, reducing the dose to critical structures or covering the entire lumpectomy volume with a margin about 5 mm all around with a particular dose line. At the same time high dose inside the target volume can be prevented and significantly reduced the dose near skin to avoid skin reactions. Plan is evaluated using natural DVH. The total dwell time is cross-checked with manual calculation as a part of pre-treatment QA.

**Results:** Ultrasound directed HDR breast implant is an extremely precise way to give radiation to the breast by applying proper optimization techniques to truly take advantage of the exquisite conformal dose capabilities of the after loading techniques.

### O-36
### *In vivo* dosimetry of electron beam radiotherapy using MOSFETS

D Manigandan, G Bharanidharan, K Devan, D Elangovan*, R Tamilarasan*, A Vasanthan*, P Aruna, S Ganesan. *Division of Medical Physics and Lasers, Department of Physics, Anna University, Chennai-600 025, Tamil Nadu, *Department of Radiation Oncology, Kailash Cancer Hospital and Research Center, Muniseva Ashram, Goraj - 391*

This PDF is available for free download from a site hosted by Medknow Publications (www.medknow.com).

[Downloaded free from http://www.jmp.org.in on Friday, July 29, 2011, IP: 130.14.254.25] || Click here to download free Android application for t

*760, Ta. Vagodhia, Dist. Vadodara, Gujarat, India*
E-mail: mpmani79@yahoo.com

**Introduction:** *In vivo* dosimetry is the most direct and independent method for monitoring the dose delivered to the patient. Generally, *in vivo* dosimetry was well established and characterized for its use in external photon beam radiotherapy for the measurement of entrance and exit doses.[1] Although the calibration and commissioning procedures of an *in vivo* detector for the photon beam is similar to that of electron beams, only limited number of publications have been studied for their use in electron beams using TLDs[2] and semiconductor diodes.[3] This may be due to the large perturbation caused by the detector. Compared to TLDs and semiconductor diodes, MOSFET *in vivo* dosimeters are being considered as alternative *in vivo* dosimeters. This is because MOSFET has several advantages that include small detector size, active area (0.04 mm$^2$), immediate reuse, immediate retrieval of information about the measured dose and its ability to conduct multiple dose measurements.[4,5] Further, MOSFET was not characterized for entrance dose measurement of electron beam radiotherapy. In this regard, MOSFET was calibrated for the clinical electron beams of 4-12 MeV energies without using any build up cap and Wide Energy Hemi-Spherical Build-up Cap (WEHSBC) was used for 15-18 MeV energies.

**Materials and Methods:** The medical linear accelerator used in this study was the Varian clinac 2300 CD (Varian Oncology Systems, Paolo Alto, CA). Five identical TN-502RD standard MOSFET detectors (Thomson Neilson electronics limited, Ottawa, Canada) were used. Absolute measurements were performed using PPC05 Markus parallel plate chamber (Scanditronix, Uppsala, Sweden). Calibration of the MOSFET was performed in a 30 x 30 x 10 cm$^3$ PMMA phantom. To determine the angular dependence of a MOSFET dosimeter, a cylindrical PMMA phantom of density 1.19 g cm$^{-3}$ was used. As the study is aimed on the measurement of dose at the depth of maximum dose, the dose at this point was obtained using the following *in vivo* dosimetry procedure. where M is the MOSFET reading, N is the Entrance Calibration Factor (ECF) in standard conditions (i.e., with field size 10 x 10 cm$^2$ cone, 100 cm Source to Surface Distance (SSD) at a dose rate of 300 MU/min etc,) and $C_i$ represents the various correction factors (viz, Field size, SSD, Angular etc.) taking in to account geometric conditions differing from standard conditions. In addition to the calibration and correction factors of the MOSFET dosimeter, MOSFET was characterized for linearity, reproducibility, fade test, sensitivity variation with accumulated dose and temperature dependence.

**Results and Discussion:** MOSFET showed an excellent linearity against an ion chamber measured dose in the dose range of 20-630 cGy. At 4-12 MeV, MOSFET showed large angular dependence in tilt directions and lesser at axial directions. At 15 and 18 MeV, MOSFET with WEHSBC showed large angular dependence in axial directions and lesser at tilt directions. This is due to the fact that increased path length of the beam through WEHSBC causes the change in response of the MOSFET at axial directions. In the case of 4-12 MeV, MOSFET was used without any build-up and it has showed significant SSD dependence and the same was lesser for 15 and 18 MeV energies when MOSFET was used with WEHSBC. As the SSD decreases, the number of contaminating X-rays and the electrons scattered by the collimating systems and blocks able to reach the MOSFET detector at the central axis. Hence the MOSFET has shown over response and the ratio of ion chamber to MOSFET decreases. In 15 and 18 MeV electron beams, these contaminating X-rays and the scattered electrons were absorbed by the WEHSBC. Since it is a dual bias dual MOSFET, no measurable effect in response was observed in the temperature range of 23-40$^{\circ}$ C. The energy dependence of MOSFET dosimeter was within 2.9% for 6-18 MeV electron beams and it was 5.44% for 4 MeV. In the study of variation of sensitivity as a function of accumulated doses, MOSFET showed increased sensitivity up to 80 Gy and then decreases linearly. Pilot clinical study was also performed and suitable calibration and correction factors were applied. All the patient measured doses were within 5% with the calculated doses. The study showed that, the MOSFET detectors are suitable for the *in vivo* dosimetry of electron beam radiotherapy in the energy range between 4-18 MeV.

### References

1. Essers M, Mijnheer BJ. *In vivo* dosimetry during external photon beam radiotherapy. Int J Radiat Oncol Biol Phys 1999;43:245-9.
2. Bartolotta A, Brai M, Caputo V, Di Liberto R, Di Mariano D, Ferrara G, *et al.* Sansone santamaria. The response behavior of LiF: Mg, Cu, P thermoluminescence dosimetry to high energy electron beams used in radiotherapy. Phys Med Biol 1995;40:211-20.
3. Alecu R, Loomis T, Alecu J, Ochran T. Guidelines on the implementation of diode *in vivo* dosimetry programs for photon and electron beam radiotherapy. Med Dosim 1999;24:5-12.
4. Chuang CF, Verhey LJ, Ping Xia. Investigation of the use of MOSFET for clinical IMRT dosimetric verification. Med Phys 2002;29:1109-15.
5. Bharanidharan G, Manigandan D, Devan K, Subramani V, Gopishankar N, Ganesh T, *et al.* Characterization of responses and comparison of calibration factor for commercial MOSFET detectors. Med Dosim 2005;30:213-8.

**O-37**

**A TLD based pilot run for non-reference conditions**

AK Mahant, SP Vinatha, Dipali Sansare, VV Shaha. *Radiation Standards Section, Radiation Safety Systems Division, Bhabha Atomic Research Centre, Mumbai - 400 085, India*

**Introduction:** The TLD based postal dose quality audits conducted by Secondary Standard Dosimetry Laboratory (SSDL), BARC is a programme to help the hospitals to improve their dosimetry status. Although currently the quality audits are conducted to check the beam output measured in the reference conditions but at the same time certain other parameters relevant to the delivered dose also require checking and for this purpose IAEA/WHO has initiated work on a programme for quality audits in non-reference conditions. This laboratory had invited twenty hospitals for a pilot run in the non-reference conditions; fifteen hospitals successfully participated in this programme. The paper gives the methodology and the results of these studies.

**Materials and Methods:** The TLD material used was the LiF: Mg, Ti (TLD-100) powder filled in capsules similar to the ones used for the

This PDF is available for free download from a site hosted by Medknow Publications (www.medknow.com)

Table 1

| Sr. No. | Irradiation geometry | Field size at SSD | Depth in water cm | Axis | Irradiation positionand dose |
|---|---|---|---|---|---|
| 1 | SSD/open | 10 cm x 10 cm | 10 | X | Centre (2Gy) |
| 2 | SSD/open | 20 cm x 20 cm | 10 | X | Centre (2Gy)<br>+5 cm (to be estimated)<br>-5 cm (to be estimated) |
| 3 | SSD/open | 20 cm x 20 cm | 10 | Y | Centre (2 Gy)<br>+5 cm (to be estimated)<br>-5 cm (to be estimated) |
| 4 | SSD/wedge | 10 cm x 10 cm | 10 | X | Centre (2 Gy)<br>+5 cm (to be estimated)<br>-5 cm (to be estimated |

[Downloaded free from http://www.jmp.org.in on Friday, July 29, 2011, IP: 130.14.254.25] || Click here to download free Android application for t

Table 2: shows the results of the audit.

| Sr. No. | Irradiation position | Axis | Number of hospitals showing deviation | | | | |
|---|---|---|---|---|---|---|---|
| | | | <2% | <3% | <5% | <10% | <15% |
| 1 | Centre 10 cm x 10 cm | X | 7 | 5 | 1 | 2 | 0 |
| 2 | Centre 20 cm x 20 cm | X | 7 | 3 | 4 | 1 | 0 |
| 3 | +510 cm x 10 cm | X | 7 | 2 | 5 | 1 | 0 |
| 4 | -510 cm x 10 cm | X | 10 | 2 | 3 | 0 | 0 |
| 5 | Centre 20 cm x 20 cm | Y | 6 | 2 | 7 | 0 | 0 |
| 6 | +5 20 cm x 20 cm | Y | 9 | 2 | 3 | 0 | 0 |
| 7 | -520 cm x 20 cm | Y | 9 | 4 | 1 | 1 | 0 |
| 8 | Centre 20 cm x 20 cm | X/W | 2 | 1 | 6 | 4 | 1 |
| 9 | +520 cm x 20 cm | X/W | 3 | 2 | 2 | 6 | 1 |
| 10 | -520 cm x 20 cm | X/W | 5 | 3 | 2 | 2 | 2 |

quality audits in reference conditions. The parameters to be checked are given below in the Table 1. A TLD irradiation stand was supplied to all the participants, which can be used to irradiate single TLD in the centre as given in the s. no. (1) in the Table 1 to deliver a dose of 2 Gy to the TLD. The stand had a separate arm which could be fixed to the stand for the simultaneous irradiation of three TLDs viz. at centre and off-axis at +5 cm and -5 cm as given in the S.No. (2) and (3). The TLD in the centre in this case is to be irradiated to a dose of 2Gy and the dose delivered to the other two TLDs located off-axis has to be estimated from the beam profile data which is generally used in the clinical practice by the hospital. Similarly three TLDs have to be irradiated with a 45° wedge. The irradiations were required to be carried out preferably in a 30 cm x 30 cm x 30 cm water phantom. The TLDs were returned to SSDL, BARC after irradiation and were evaluated using a Harshaw - 2000 TLD reader.

**Conclusion:** The results have shown that though the majority of the hospitals are able to give good results in the reference condition but there is a need for improvement as regards the dosimetry in the non-reference conditions with a special emphasis on the measurements of the wedged fields.

This PDF is available for free download from a site hosted by Medknow Publications (www.medknow.com).

### O-38

### A review on the functioning of directorate of radiation safety in Kerala

B Radhakrishnan, G Ramakrishnan. *Directorate of Radiation Safety, Government of Kerala, Calicut - 8, India*

**Introduction:** This paper attempts to highlight the functioning of Directorate of Radiation Safety (DRS) in the state of Kerala and emphasizes the significance of formation of such Directorates in other parts of the country in implementing the RPR in radio-diagnostic installations.

**Formation of DRS:** The Directorate of Radiation Safety (DRS) was formed in the year 1998 by the Government of Kerala after proper discussions with the AERB and started functioning in the same year. A set of equipments needed for radiation safety and quality control programmes were also procured. The AERB has also authorized the DRS to exercise the powers conferred by Rules 29, 30, 31(a), (b), (c), (e) and (f) of RPR 1971 promulgated under section 30 of the Atomic Energy Act, 1962. Functioning of DRS: The DRS has identified 2560 X-ray units and 120 CT scanners used in the state of Kerala. The number of IITV X-ray units identified was about 250 and dental units were around 100. Besides 20 DSA and CATH labs were also identified. Units satisfying the basic requirements of radiation safety were less than 10% before the functioning of the DRS. However, much improvement in the situation has taken place after the functioning of the DRS. The radiation safety requirements could be successfully enforced after the Government of Kerala amended the Electricity Act. As per the amendment, electrical connection to any new radiological equipment will be given only after the user producing a radiation safety clearance certificate from the DRS after incorporating the basic requirements of radiation safety in and around the installation. The power connection to the existing installation which does not have proper radiation safety status will also be disconnected if a letter from the Directorate of Radiation Safety to that effect is sent to the Kerala State Electricity Board and power will be resumed only after getting a radiation safety clearance certificate from the DRS. Thus defaulting institutions are taken care of. So far, 780 installations that satisfy the basic requirements have been authorized by the DRS. This authorization is given for a period of one year. Recently the Government has also provided a vehicle to DRS.

**Organisation:** The DRS has the following technical personnel to manage the activities:

| Position | No. of posts |
|---|---|
| Director | 1 |
| Radiation Safety Inspector | 4 |
| Technical Assistants | 4 |
| Administrative staff | 2 |
| Driver | 1 |
| Peon | 1 |

**Other Activities:** Since the formation, the Drs have been requested to inspect and report on the instances of 9 overexposures in the state of Kerala by AERB. Academic activities are also conducted by the DRS. recently in April 2006, a workshop was conducted for the radiographers in which around 200 participants had attended. Limelights: Consequent to a writ petition, the Honourable Supreme Court of India has served notices to all the States and sooner or later DRS will be formed in all the states. The Medical Council of India now insists from the Medical Colleges in Kerala to produce a radiation safety clearance certificate issued by the DRS. The MCI insisted for this after getting convinced of its importance and relevance, especially after the Supreme Court has started hearing the writ petition. Approval certificates of X-ray units are issued by the AERB after getting report from the DRS.

**Conclusion:** DRS in Kerala have made considerable progress in implementing the RPR in radiological installations. However, lot has to be done in completing quality assurance tests, random checking of the installations, etc. The pace is slow due to non-availability of staff in full strength and shortage of funds, which may be overcome in the coming years.

### O-39

### Monitoring of workers at plant site with shielded chair whole body counter of a mobile radiological laboratory

P Vijayagopal, Suma Nair, SP Garg, KA Pendharkar. *Internal Dosimetry Division, Bhabha Atomic Research Centre, BARC Hospital, Mumbai-400 094, India*

**Introduction:** A Mobile Radiological Laboratory (MRL) has been developed at BARC for quick deployment to assess the radiological impact in the environment in the event of an accident involving radioactive material. Real-time measurements and the fast analysis of the results are of crucial importance in a radiological emergency. For a timely response, MRL is equipped with necessary radiation measuring devices to carry out the required environmental and radiological monitoring. For measurements of internal exposure, a shielded chair Whole Body Counter (WBC) has been installed in MRL. WBC was calibrated with the aid of BOMAB (Bottle Mannequin Absorber)

[Downloaded free from http://www.jmp.org.in on Friday, July 29, 2011, IP: 130.14.254.25] || Click here to download free Android application for t

Table 1: Details of the workers monitored and the MDA values (for 5 minutes counting) for three important radionuclides

| *No. of persons monitored* | *Radionuclide* | *MDA (kBq)* | *Remarks* |
|---|---|---|---|
| 275 | I-131<br>Cs-137<br>Co-60 | 0.77<br>0.69<br>0.24 | In most of the cases activity detected was Below detection limit |

phantoms. The calibrated WBC was field tested by taking MRL to Kakrapar Atomic Power Station, where 25 persons from general population residing near the site were monitored for internal contamination. In order to keep the vehicle in ready condition for conducting internal contamination monitoring in case of radiation emergency, MRL was utilized for conducting whole body counting of employees working at different plants in BARC, Trombay. MRL was parked at a convenient place near the plant facility for carrying out the monitoring. In this paper we describe about the monitoring of workers at plant site with shielded chair whole body counter.

**Materials and Methods:** A specially designed shielded chair type whole body counter installed in MRL was used for this study. The chair shield is designed for equivalent lead thickness of 90 mm for back and 70 mm for sides. The chair is tilted backward by 15° to provide ease in getting in and out of the chair and comfort to the subject. The 300 kg detector shield, housing a 102 mm dia x 76 mm thick NaI(Tl) detector, is fixed to a horizontal arm which can be revolved to allow the subject in and out of the counting position and rotated to facilitate monitoring of specific body section. The WBC was calibrated with Ba-133 (simulating I-131), Cs-137 and Co-60 with the aid of five age dependent BOMAB phantoms covering age range 1-2, 2-7, 7-12, 12-17 year old children and adult. The calibration factors varied from 38.12 to 47.00 cpm/kBq for $^{131}$I, from 21.28 to 30.35 cpm/kBq for $^{137}$Cs and from 49.01 to 55.84 cpm/kBq for $^{60}$Co, from higher age group to lower age group. The minimum detectable activity varied from 637 to 773 Bq for $^{131}$I, from 491 to 699 Bq for $^{137}$Cs and from 222 to 244 Bq for $^{60}$Co, from lower age group to higher age group for a counting time of 5 minutes.

**Results and Discussion:** Employees working in various facilities in BARC, Trombay were monitored for internal radioactive contamination by shielded chair WBC in MRL. MRL was parked at a convenient location near the plant facility for carrying out the monitoring. Three criteria were considered for location of MRL viz. detector background, shower and change room facilities and power availability. All persons before counting are required to change the clothes from normal plant work apparel. A total of 275 subjects were monitored from various facilities in BARC, Trombay. Monitoring of workers in MRL at plant site has benefited in keeping the system in ready condition for deployment in public domain in case of any radiation emergency.

### O-40

### Biological dosimetry in cancer patients undergoing partial body irradiation using micronucleus assay

S Senthamizhchelvan, GS Pant, Omana Nair*, PK Julka*, GK Rath*, M Pandey*, R Prabhakar*, KP Haresh*. *Departments of Nuclear Medicine and *Radiotherapy, AIIMS, New Delhi, India*

**Introduction:** Biological dosimetry is the measure of a given physiological response to a known or estimated exposure dose of ionizing radiation. Reliable biological dosimeters of radiation exposure are needed (1) to handle casualty triage, (2) to establish probable exposures when physical dosimeters are absent, (3) to confirm physical dosimetry and (4) to monitor the progress of radiotherapy. The micronucleus (MN) yield in peripheral blood lymphocytes (PBL) serves as one of the quantitative biodosimeters for monitoring *in vivo* ionizing radiation damage.

**Objectives:** To study the applicability of the micronucleus (MN) assay as biodosimetry in cancer patients undergoing radiotherapy and also to study the relationship between the MN yield in PBL and the estimated effective whole body dose (EWBD).

**Materials and Methods:** This study was performed in 56 cancer patients (50 male, 6 female and Age 56 ±11 y) who had no prior history of chemotherapy or radiotherapy. Blood samples were obtained before therapy and once randomly during radiotherapy. The culture setup for MN assay consisted of 0.5 ml of whole blood in RPMI 1640 medium containing 10% fetal bovine serum, 1% PHA-M and the cultures were incubated at 37°C. Cytochalasin-B (6 mg/ml) was added at 44h after initiation of cultures to block the cells at cytokinesis. The cells were harvested at 72h by centrifugation and treated with 0.125M KCl solution at room temperature for 6min. The cells were fixed first with fixative solution containing a mixture of methanol, acetic acid and 0.9% NaCl solution (12:6:13) at ice-cold temperature and allowed to stand for 6 min and centrifuged. The cell button was fixed again with a chilled mixture of acetic acid and methanol (1:4) for 5 min. The final cell button was suspended in 0.5 ml of fixative and mixed well by gentle flushing and spread on a cold slides. At least 1000 CB cells were scored per sample as per standard procedures. Results and conclusion: The incidence of MN in the baseline samples of cancer patients was significantly high as compared to normal incidence. A linear relationship has been obtained for radiotherapy induced micronuclei and equivalent whole body dose. Least squares regression analysis and Pearson's correlation coefficient were estimated for the linear model. To compare the aberration yield distributions relative to Poisson distribution, the variance to mean ratio ($\sigma^2/y$) and dispersion index ($\mu$) values were determined. The relation between MN yield (Y) and EWBD (D) is Y = 72.6D+36.3, ($R^2$ = 0.92, $P$ = 0.01). This dose response relationship can be used to estimate the equivalent whole body dose for a known incidence of MN in fractionated radiotherapy.

**Acknowledgement:** This work has been supported in part by Atomic Energy Regulatory Board (AERB) through its research contract No. AERB/SRP/26/03/2002.

### O-41

### Evaluation of clinical significance of cumulative biological effective dose and overall treatment time in the treatment of carcinoma cervix

A Mandal, AK Asthana, S Pradhan, UP Shahi, LM Aggarwal. *Departments of Radiotherapy and Radiation Medicine, Institute of Medical Sciences, Banaras Hindu University, Varanasi - 221 005, India*

**Introduction:** Radiation therapy is standard treatment in all stages of carcinoma of cervix, which includes External radiotherapy (EBRT) and Intracavitary brachytherapy (ICBT). Each component play important role and balancing of dose from EBRT and ICBT depends on stage of disease. The purpose of this retrospective study is to report the response and complications on the basis of cumulative biologic effective dose (BED) and overall treatment time (OTT).

**Materials and Methods:** 64 (Stage II-35/64, Stage III-29/64) previously untreated patients with cervical cancer were treated with a combination of EBRT and low dose rate ICBT. The cumulative BEDs were calculated at Point A ($BED_{10}$) and bladder, rectal reference points ($BED_{2.5}$) using the linear-quadratic BED equations.[1] The equations of BEDs in fractionated and continuous treatments are as following [A] and [B]:

$$BED_{fr} = ND\left[1 + \frac{D}{\alpha/\beta}\right] \quad [A]$$

N = Number of fractions
d = Dose per fraction (Gy)
D = Total dose (Gy)
$\alpha/\beta$ = Tissue specific parameter = 2.5 Gy (Late reacting normal tissue) and 10 Gy (Tumors)

$$BED_{cont} = RT\left[1 + \frac{2R\,(1 - 1/\mu T)}{\mu\,(\alpha/\beta)}\right] \quad [B]$$

R = Dose rate (Gy/hr)

This PDF is available for free download from a site hosted by Medknow Publications (www.medknow.com).

[Downloaded free from http://www.jmp.org.in on Friday, July 29, 2011, IP: 130.14.254.25] || Click here to download free Android application for t

T = Application time (hr)
$\mu$ = Tissue specific parameter = 0.46 $hr^{-1}$ (Late reacting normal tissue) and 1.40 $hr^{-1}$ (Tumors)
The median of calculated cumulative $BED_{10}$ and $BED_{2.5}$ was 84.53 (range 72.67-89.71) and 108.44 (range 73.19-126.19) Results: The follow-up ranges between 19-90 months with a median of 37 months for all patients. Survival and local control: Out of 35 patients of carcinoma of cervix stage II, 17 received $BED_{10}$ < 84.5 and 18 received the $BED_{10}$ > 84.5. The local control (LC) rate and 5 year disease free survival (DFS) rate was 76.47%, 76.02% for $BED_{10}$< 84.5 and 77.77%, 73.85% for $BED_{10}$ > 84.5. In stage II patients, 16 received the full course of radiotherapy in <50 days and 19 in > 50 days. The LC rate and 5 year DFS rate was 75.00% and 73.33% for < 50 days and 78.95%, 76.26% for > 50 days. Out of 29 patients with carcinoma of cervix stage III, 14 received $BED_{10}$ < 84.5 and 15 received the $BED_{10}$ > 84.5. The LC rate and 5 year DFS rate was 78.57%, 64.29% for $BED_{10}$< 84.5 and 93.33%, 93.99% for $BED_{10}$ > 84.5. This distribution is statistically significant (*P* < 0.01). In stage III patients, 12 received the full course of treatment in <50 days and 17 in > 50 days. The LC rate and 5 year DFS rate was 100.00%, 100.00% for < 50 days and 76.47%, 68.63% for>50 days. Normal tissue complications: Out of 64 patients 18 received the $BED_{2.5}$ <105 at rectal and bladder points and 46 patients received $BED_{2.5}$ >105. Of these 18 patients, 4 (22.22%) had rectal complications (2 patients Gr-II, 1 patient Gr-III, 1 patients Gr-IV and 3(16.67%) had bladder complications (1 patient Gr-II, 2 patients Gr-III) bladder complication. 77.78%, 83.33% and 66.67% was the rectal complication free survival ($CFS_R$) rate, bladder complication free survival ($CFS_B$) rate and all type late complication free survival (CFS) rate respectively at 90 months. Out of 46 patients, 24 (52.14%) had rectal complications (7 patients Gr-I, 9 patients Gr-II, 6 patients Gr-III and 2 patients Gr-IV) and 8 (17.39%) had bladder complications (4 patients Gr-II, 2 patients Gr-III and 2 patients Gr-IV). The CFS(R) rate, CFS (B) rate and CFS rate at 90 months was 44.44%, 65.31% and 23.93% respectively.
**Conclusions:** In this study, it has been observed that patients with higher $BED_{10}$ (>84.5) show higher LC and DFS rate than the lower $BED_{10}$ (<84.5) in stage II and stage III (*P*<0.01). The LC and DFS are also higher in patients with lower OTT (< 50 days) than higher OTT (> 50 days) in stage II and stage III (*P*<0.001). Patients with lower $BED_{2.5}$ (<105) had less rectal (*P*<0.001), bladder complication rate and higher CFS rate than higher $BED_{2.5}$ (>105). Therefore, it can be concluded that to achieve higher tumor control with less normal tissue complications $BED_{10}$ should be more than 84.5 and $BED_{2.5}$ should be less than 105 delivered in less than 50 days.

### References

1. Dale RG. The application of the linear-quadratic dose-effect equation to fractionated and protracted radiotherapy. Br J Radiol 1985; 58:515-28.

### O-42

### Bioeffect dose in cancer of cervix to assess radiation response

Challapalli Srinivas, K Kamalaksh Shenoy, Jayarama Shetty, K Dinesh Pai, SS Supe*, JGR Solomon***. *Department of Radiotherapy, Kasturba Medical College Hospital, Attavar, Mangalore - 575 001,*Department of Radiation Physics, KMIO, Bangalore - 560 029, **Department of Radiotherapy, KMC, Manipal - 576 119, India*

Introduction: Cancer of cervix is the second most common cancer worldwide among women (IARC, 1992). Several treatments related protocols of radiotherapy have been followed over last few decades in its treatment for evaluating the response. These physical doses varying on the basis of fractionation size, dose rate and total dose need to be indicated as bio-effect dose (BED) to rationalize these treatments. There is a need for investigation in these areas and this study will address these aspects. Aims and Objectives: To correlate Bio Effect Dose with local tumor response in patients of carcinoma of cervix undergoing External Radiotherapy (EBRT) followed by Brachytherapy (ICBT). Materials and Methods: Between June 1998 and July 2004, 154 patients with carcinoma of the cervix stage IIB (n = 42) and IIIB (n = 112) were included in this study. All patients were treated with EBRT (using various fractionated schedules) followed by BRIT manual after loading LDR $^{137}$Cesium ICBT (25 to 30 Gy to point A). Patients were followed-up every 3 months. Statistical analysis was performed with a software program SPSS (Ver 11.0) to find the correlation between the overall BED and response.
BED calculation
BED to the point A in EBRT and ICBT applications is calculated according to Orton and Dale's formula and (1991) as given below:

$$\text{BED (EBRT - A)} = \text{nd.}\left[1 + \frac{d}{\alpha/\beta}\right] \text{Gy} \qquad (1)$$

$$\text{BED (ICBT - A)} = \left[1+ \frac{2R}{\mu}\left(\frac{\beta}{\alpha}\right)\left\{1 - \frac{1}{\mu T}\left[1 - e^{(-\mu T)}\right]\right\}\right].\text{R.T Gy} \qquad (2)$$

Where
n = number of # in EXRT; d = dose per # (Gy); ($\alpha/\beta$) = 10 for Point 'A'
R = Dose rate to point A in ICBT (Gy/hr); $\mu$ = Time const. = $0.693/t_{1/2}$; $t_{1/2}$ = 1 hr for Point 'A'; T = Total treatment time of ICBT (in hrs)

Over all Bioeffect dose to point A ($BEDA_{10}$) was calculated as
$BEDA_{10}$ (Gy) = $BED_{(EBRT-A)}$ + $BED_{(ICBT-A)}$ - k ($T_{tot}$ - Td) ........(3)

Where,
k is the repair half time = 0.5 Gy/day
$T_{tot}$ is the Overall treatment time of EBRT in days
Td is the potential doubling time in days = 28 days (Orton 1991).

**Results and Discussion:** It is been observed that mean $BEDA_{10}$ in stage IIB and IIIB is 81.3 and 81.0 respectively. Mean $BEDA_{10}$ for stage IIB patients with complete response (n=33: 78.5%) was 83.1 (sd= 4.88) and those with local recurrence (n=9: 21.5%) was 75.0 $Gy_{10}$ (sd=5.11) (*P*=<0.001). Similarly $BEDA_{10}$ for stage IIIB with CR (n=91: 81.3%) was 81.8 (sd=4.59) and those with LR (n=21: 18.7%) was 77.3$Gy_{10}$ (sd=5.96) (*P*=<0.003). In stage IIB, there are 9 patients (53%) with BEDA of less than 81 $Gy_{10}$, with CR as against 8 patients (47%) who had LR. With BEDA of ≥81 $Gy_{10}$, there are 24 patients (96%) with CR as against one patient (4%) with LR ($\chi^2$ = 8.732, p<0.003). In stage IIIB, BEDA of <82 $Gy_{10}$ was seen in 46 patients (71.8%) with CR as against 18 patients (28.2%) who had LR. For BEDA of ≥82$Gy_{10}$, there are 45 patients (93.7%) with CR as against 3 patients (6.3%) who had LR ($\chi^2$ = 8.615, *P*<0.002).
**Conclusion:** From this study we conclude that the mean $BEDA_{10}$, with 81 and 82 $Gy_{10}$ serves as an effective BED in bringing significant response under stage IIB and IIIB respectively.

### O-43

### A programmable OSL reader system for medical and research applications

MS Kulkarni, DR Mishra, NS Rawat, SS Sutar, DN Sharma. *Radiation Safety Systems Division, Bhabha Atomic Research Centre Mumbai - 400 085, India*

**Introduction:** The optically stimulated luminescence (OSL) technique of dose measurement is becoming very popular for radiation dosimetry using OSL phosphors. The OSL measurement systems are not commonly available for the medical and research applications unlike commercial TL reader systems. The automated OSL measurement systems commercially available for the OSL measurements are highly biased for their use in environmental dosimetry for dating of archeological and geological samples. Also, such reader systems are very expensive and not commercially viable for the medical and research institutions for routine radiation dosimetry applications. A major concern in the design of the OSL reader is to ensure that the light detection system should respond only to the light emitted from the sample, but not to the stimulating light source. In the continuous wave (CW) OSL mode this is achieved by incorporating appropriate interference filters

This PDF is available for free download from a site hosted by Medknow Publications (www.medknow.com)

[Downloaded free from http://www.jmp.org.in on Friday, July 29, 2011, IP: 130.14.254.25] || Click here to download free Android application for t

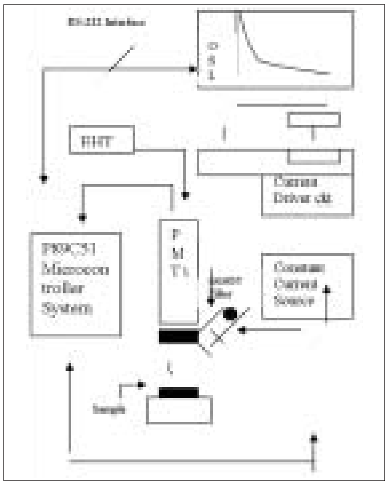

**Figure 1: OSL reader setup**

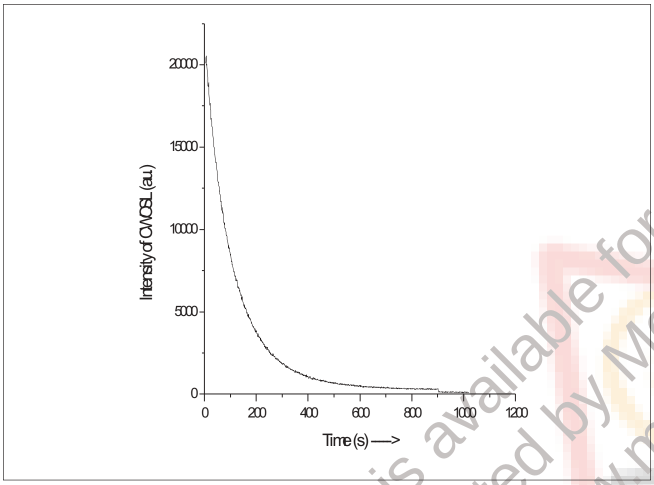

**Figure 2: Typical OSL curve of $Al_2O_3$:C sample**

between the sample drawer and the PMT housing. In another method known as the pulsed OSL (POSL) method, a pulsed laser with approximately 300 ns pulse width is used as a stimulation light source and the luminescence is recorded during the intervals between the pulses. The use of such pulsed laser systems in POSL and interference filters in CWOSL along with expensive optics increases the cost of the reader system. This paper presents the development of a simple cost effective CWOSL reader system incorporating light emitting diode (LED) clusters as a stimulating light source and inexpensive optical color glass filters for measurement of OSL signal from the phosphor material and its application in OSL based radiation dosimetry.

**Description:** The CWOSL reader system consists of two parts. The first part is comprised of lightproof drawer assembly for loading of phosphor material, stimulation light unit, photon counting module (for luminescence detection) and a drawer for housing optical filters. The second part consists of electronic circuitry, high current driver board for LED cluster, power supply units, PC connectivity etc. The block diagram of the OSL reader is shown in Figure 1. The system can be used for analysis of material up to 10 x 10 mm size. A super-bright LED clusters that yields a light output of 5.0 W carries out the optical stimulation of the samples. The light intensity focused on the sample position from LED cluster with peak wavelength $\lambda_p \approx$ 470 nm or 530 nm and $\Delta\lambda \approx$ 20nm. A GG-435 color glass filter is used to narrow $\Delta\lambda$ of the stimulating light beam and UG 1 filter to cut off the stimulating wavelength from reaching the PMT. An optical coupler has been provided on the light stimulation assembly to connect an external laser beam (operating in 470 nm to 550 nm range) through an optic fiber. The user can select the LED cluster (blue or green) or the laser as a stimulating light source depending on the OSL samples under study. The electronic circuitry of the reader system is based on a single chip microcontroller (Phillips 89CC51) and the basic hardware consists of a 12-bit DAC (MAX 539), a 12-bit ADC (MAX 1241), MAX2323 for serial transfer of data to the PC, a high current source capable of supplying 700mA dc current to the LED cluster, etc. The stimulating light power at the sample position is recorded when a photodiode placed in the drawer assembly is brought below the PMT. The OSL output is recorded using photon-counting module. The reader is interfaced to PC through an RS-232 serial interface. The assembly language software for 89C51 microcontroller controls the entire operation of the reader on command from the computer.

**Results and Conclusion:** The introduction of CWOSL reader system will facilitate alternative low cost indigenous OSL readers for dose measurements in radiation dosimetry and research applications. The reader parameters like stimulating light wavelength, stimulating light intensity, readout time, selection of optical filters, data transfer rate can be set by the user depending on the OSL sample under study. The OSL samples can be stimulated using CWOSL and LMOSL modes with a 470 nm stimulation wavelength with the light intensity > 100 mW/$cm^2$. The Figure 2 shows a typical CWOSL curve of an $Al_2O_3$:C sample obtained using the above reader system. A minimum dose of 50 $\mu$Gy could be detected for an indigenously developed $\alpha$-$Al_2O_3$:C samples using the above OSL measurement setup. The dose reproducibility for an absorbed dose of 10.5 mGy from a $^{90}$Sr/$^{90}$Y beta source was within ± 5%. The alternate laser light stimulation source provision in the reader system increases the reader capability for the RandD applications. The OSL data is transferred to a PC through an RS-232 serial interface and stored in a user-defined file for later analysis. As the OSL data is stored in ASCII format, the data can be analysed by applying different algorithms using commercial software packages.

This PDF is available for free download from a site hosted by Medknow Publications (www.medknow.com).

### O-44
### Commissioning and QA issues of KONRAD inverse planning system for IMRT

Shrikant Deshpande, VK Sathiyanarayanan, Janhavi Bhangle, Kumara Swamy, Sumit Basu. *Ruby Hall Clinic, Pune, India*
E-mail: mpshri11@rediffmail.com / vsathiya7@rediffmail.com

**Introduction:** The KONRAD inverse planning system with Siemens Oncor dual energy Linear accelerator has been commissioned in our clinic for IMRT (Step and Shoot) treatment planning and delivery. The beam data required for commissioning were generated and relative and absolute dose measurements were carried out before clinical implementation. This communication reports preliminary results and perspective issues of commissioning this system.

**Materials and Methods:** KONRAD uses the "Weighted quadratic difference of prescribed and calculated dose distribution method" for the inverse planning algorithm to calculate the optimal intensity profiles (fields). The intensity modulated fields are divided into beamlets that can be delivered by means of sequence of leaf setting by the MLC. To ensure that what is delivered is same as calculated, the entire plan has been overlaid on the axial image set of the IMRT verification phantom. The entire treatment is delivered to the phantom and the integrated dose at predetermined points using the CC01 ion chamber is measured in the IMRT phantom (Scanditronix-Welhofer). The measured absolute dose and predicted doses from TPS were compared. Comparison shows excellent agreement (< 3%) at most of the measurement points. The film dosimetric QA of the IMRT is carried out by positioning the ready pack EDR2 film, at a particular axial image plane and delivering the entire treatment. The axial slice is in the Z-axis in the Konrad and at particular Z-level the film is placed. The dose profile in the DICOM format in that particular Z-level, where the film was positioned, is exported to the Omnipro-IMRT software. The measured fluence (or distribution) and the calculated are compared using the Omni Pro IMRT software. Excellent correlation (correlation coefficients > 0.97) was found for different axial plane between measured and predicted dose.

[Downloaded free from http://www.jmp.org.in on Friday, July 29, 2011, IP: 130.14.254.25] || Click here to download free Android application for t

**Results and Discussion:** Out of 20 patients the absolute dose varies from 0.24% (Min.) to 8.6%. The absolute dose variation exceeds 3% in 5 patients out of 20. The correlation coefficient (in case of Film Dosimetry) is ranging from 0.9715 to 0.9931 in all the patients. The gamma value in all the patients was less than 1.5%. The absolute dose variation in case of 5 patients was mainly due to the ionization chambers location was in the high dose gradient region. The Oncor Impression plus Accelerator is equipped with Optifocus 82 leaf MLC. Although maximum field size that can be covered is 40x40, the maximum length of the target organs that can be covered by the intensity modulated fields is 20x20 cm$^2$. This is due to the over travel constraint of the jaws and MLC banks. The sequencer gives the option of profile smoothening, which smoothens the fluence pixels along the path in two ways namely 1D and 2D. Also planner can choose the number of intensity levels which finds deliverable solution which is closer to optimal continuous distribution. Higher number of levels, closer will be the deliverable solution to optimal continuous distribution, which further increases number of segments, introducing few low MU segments. One important issue in Step and Shoot methodology is to avoid low MU segments. The sequencer arrives at shapes in such a way all the shapes more or less have equal MUs. We choose optimal intensity level, either 7 or 10 although highest level available is 15. On the treatment delivery side, it takes around 7 minutes for delivering 70 segments over 6 gantry angles arranged in the auto field sequencing.

### O-45

### Commissioning and clinical implementation of elekta MLC based IMRT with CMS XIO inverse treatment planning system

NS Shine, KV Rakesh, Joby Mathew, Satheesh P Prasad, Bhaskaran K Pillai. *Department of Radiation Oncology, Amrita Institute of Medical Sciences, Kochi, India*

**Introduction:** This presentation details commissioning and clinical implementation of IMRT with Elekta MLC and CMS XiO treatment planning system. The "step-and-shoot" technique is used for this MLC-based IMRT. Particular attention is paid to features unique to this hardware and software that are not addressed in general IMRT guidance literature.

**Materials and Methods:** An Elekta Precise LINAC, equipped with 4,6,15 MV photon beams and 80 leaves MLC was commissioned for clinical usage along with CMS XiO 4.3 RTP system. Commissioning process requires the verification of predefined parameters available on the CMS XiO RTP System and the collection of some machine data. The machine data required are TSCFs down to a 1x1 collimator setting, PDDs down to 2x2 open fields, MLC transmission, Collimator transmission, Profiles at different depths for MLC formed field of size 2x10. IMRT beam was modeled and checked the goodness of the MLC sigma parameter set in the beam modeling by comparing the measured 2x10 MLC profiles with TPS generated profiles particularly ensured excellent penumbra agreement. QA procedure follows the recommendation of the AAPM Task Group No. 40 report. In addition, the leaf position accuracy and reproducibility of the MLC checked at regular intervals. The dose validation is implemented through the hybrid plan where the patient beam parameters are applied to a flat phantom and verified by evaluating gamma using *MapCkeck* ionization chamber array and its software.

**Conclusion:** The Physicist must be aware of leaf and jaw motion constrains that are unique to Elekta accelerator and how they are used in IMRT segment delineation by CMS XiO planning system. When these factors are considered in hardware calibration and beam modeling, dose accuracy of 3% and positional accuracy of 2 mm are achievable over a range of stringent tests and clinical plans.

### O-46

### Surface dose measurements of telecobalt unit by extrapolation technique using Gafchromic EBT film and flatbed documents scanner

Rajesh Kumar, SD Sharma, Rituraj Upreti*, Mahua Basu, Reena Ph**, S Kannan. *Radiological Physics and Advisory Division, Bhabha Atomic Research Centre, CT and CRS Building, Anushaktinagar, Mumbai - 400 094, *Department of Medical Physics, Tata Memorial Hospital, Mumbai-400 012, **Department of Radiation Oncology, ACTREC, Kharghar, Navi Mumbai, India*
E-mail: raj_resh@rediffmail.com

**Introduction:** An accurate measurement of the surface dose of teletherapy units is a difficult task but it is important for quality assurance and clinical dosimetry. In many clinical situations, accurate knowledge of surface dose helps in deciding the optimum treatment parameters (i.e., beam energy, inclusion/omissions of bolus, techniques etc.) reduces the risk of sub-dermal fibrosis. The instrument of choice of these measurements is ideally extrapolation chamber but very few centres have this instrument. As a result many techniques with correction and accuracy limit, such as fixed separation parallel plate chamber, TLD extrapolation technique as well as monolayer of TLD powder have been successfully used for surface dose measurements. All these methods are laborious as well as time consuming. In the present study a simple method using Gafchromic EBT film (ISP Technologies Inc. USA) and flatbed documents scanner is used for the surface dose measurements.

**Materials and Methods:** Surface dose measurements were carried out on indigenously developed Bhabhatron telecobalt unit that have $^{60}$Co source of diameter 2 cm and source to collimator distance of 47.5 cm.[1] The surface dose was measured for field sizes 5x5 cm$^2$, 10x10 cm$^2$ and 15x15 cm$^2$. Extrapolation technique was performed by irradiating a stack of five 1.5x1.5 cm$^2$ films placed in an appropriate groove at the center of 30x30x3 cm$^3$ perspex plate. This perspex plate containing the stack of EBT films was placed over the full scatter phantom. The film stack was then irradiated for 4 Gy at the depth of maximum dose ($d_m$) using field size of 10x10 cm$^2$ at an SSD of 80 cm. Similar procedures was followed for other field sizes. Film of size 1.5x1.5 cm$^2$ by placing at $d_m$ of telecobalt beam was also irradiated for 4 Gy to measure the output of the unit and reading of this film was used to normalize the reading of films in the stack. All the EBT film samples were evaluated 24 hrs after the irradiation. The films were analyzed using EPSON Expression 10000XL flatbed document scanner with ImageJ software. The thickness was measured using calipers and was found to 0.24 mm. Film density was also determined and it was found to 1.34 mg/mm$^3$. Using thickness and density values effective water equivalent thickness of the Gafchromic EBT film was calculated to 0.32 mm. The surface dose for the field sizes 5x5 cm$^2$, 10x10 cm$^2$ and 15x15 cm$^2$ were also measured using parallel plate chamber (model 23343, PTW Freiberg, Germany) by Gerbi *et al* method.[2]

**Results and Discussions:** Figure 1 shows extrapolated curve obtained from the exposed stack of films for three different field sizes namely 5x5 cm$^2$, 10x10 cm$^2$ and 15x15 cm$^2$ as a central axis percentage build up dose measurement within the first few millimeters. The surface dose measured using the parallel plate chamber by Gerbi et al methods

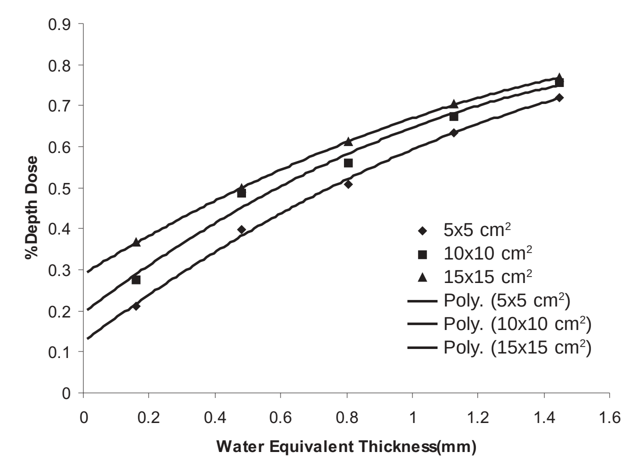

**Figure 1:** Surface dose of Bhabhatron using EBT film extrapolation technique

This PDF is available for free download from a site hosted by Medknow Publications (www.medknow.com).

[Downloaded free from http://www.jmp.org.in on Friday, July 29, 2011, IP: 130.14.254.25] || Click here to download free Android application for t

Table 1: Comparisons of surface dose of Bhabhatron-I using EBT film extrapolation technique and Parallel plate chamber

| Techniques | 5x5 cm$^2$ | 10x10 cm$^2$ | 15x15 cm$^2$ |
|---|---|---|---|
| Parallel Plate Chamber (Gerbi et. al. methods) | 12.44 | 20.6 | 30.4 |
| EBT extrapolation | 12.47 | 19.69 | 29.00 |

is also shown in the Table 1 for comparison. The effective point of measurement was assumed to be at the center of each film and thus the results for each film layer are quoted at half the water equivalent thickness, i.e., 0.161 mm. Due to the nonlinear nature of photon build up characteristics, a second order polynomial curve extrapolation was used as the line of best fit to determine surface dose. The result shows that EBT film extrapolation technique measured surface dose values are comparable with values measured using parallel plate chamber by Gerbi *et al* methods.

**Conclusions:** Surface dose values for different field sizes of telecobalt unit were measured using EBT film extrapolation technique and flatbed document scanner. The values are comparable with surface dose measured using parallel plate chamber by Gerbi *et al* method. EBT film extrapolation technique with flatbed document scanner is simple and suitable technique for the routine measurement of surface dose.

**References**

1. Kumar R, Sharma SD, Phurailatpam R, Deshpande DD, Kannan S. Performance characteristics of indigenously developed Bhabhatron-I telecobalt unit. J Med Phys 2005;30:41-7.
2. Gerbi BJ, Khan FM. Measurement of dose in the build-up region using fixed separation parallel-plate chambers. Med Phys 1990;17:17-26.

### O-47

### Impact of different breathing conditions on the dose to surrounding normal structures in tangential field breast radiotherapy

R Prabhakar, T Ganesh, M Pandey, PK Julka, GK Rath, RC Joshi, AK Bansal, PS Sridhar. *Department of Radiotherapy, Institute Rotary Cancer Hospital, All India Institute of Medical Sciences, New Delhi - 110 029, India*
E-mail: rampraba@rediffmail.com

**Introduction:** Treatment of early breast cancer by radiotherapy after conservative surgery improves the local control, however improvement in treatment outcome must always be balanced with the potential risk of long-term complications such as late cardiac mortality and radiation-induced pneumonitis. The challenging parameters, which interfere in achieving the treatment outcome and complications, are organ motion and setup-errors. Organ motion is one of the serious concerns in radiotherapy and with the introduction of newer treatment approaches like image guided radiotherapy; the importance of organ motion in treatment planning has been highlighted. In this study an effort has been made to study the dose to surrounding normal structures such as heart, lung, contralateral breast (CLB) and liver due to three different voluntary breathing conditions.

**Materials and Methods:** Thirteen patients with early breast cancer who underwent conservative surgery: nine left-sided and four right-sided breast cancer patients were selected in this study. Prior to imaging, the patients were trained to hold their breath in deep inspiration and deep expiration. The area of CT scanning included the superior (cranial) and inferior (caudal) border of the field marked by the radiation oncologist with an additional margin. Spiral CT scans were performed in Siemens Volume Zoom CT for all the three breathing conditions viz. Deep inspiration breath-hold (DIBH), normal breathing hold (NB) and deep expiration breath-hold (DEBH). The average time for which the patients were asked to hold their breath was 15-18 sec. The CT image data sets were pushed to the treatment planning system through network. Structures such as Body (external contour), Planning Target Volume (PTV), Ipsilateral Lung (IL), Contra-lateral Lung (CLL), Heart, Contra-lateral Breast (CLB) and Liver were delineated on NB, DIBH, DEBH reconstructed 3DCT datasets. Conventional tangential fields were placed on the 3D-CT dataset by isocentric technique with matching posterior field borders. The isocenter is placed at the center of mass of the PTV with the help of the tool available in the planning system. For each patient, simple tangential field plans were created for the three different CT data sets and DVH analysis were performed for the following structures: CTV, heart, ipsilateral lung, contralateral lung, liver and contralateral breast. Parameters such as V50 (volume covered by dose >50Gy Isodose) for heart, ipsilateral lung, liver, V30 (volume covered by dose >30Gy Isodose) for heart, V20 (Volume covered by dose >20Gy Isodose) for ipsilateral lung were noted down.

**Results:** The cardiac dose was significantly reduced in DIBH condition as compared to NB by 0-73.5% (median 21.69%). The volume covered by dose >50Gy for liver (right breast) ranged between 4.03 cc - 31.48 cc (median 11.39 cc, mean 14.57 cc), 0 cc - 29.78 cc (median 3.79 cc, mean 9.34 cc), 5.69 cc- 73.24 cc (median 18.14 cc, mean 28.8 cc) for NB, DIBH and DEBH respectively. However, for ipsilateral lung, even though IBH resulted in reduced dose, it was only marginal. Dose to contralateral lung was not affected by different breathing conditions. For right breast cancer, DIBH resulted in excellent liver sparing.

**Conclusion:** Our results indicate that in patients with breast cancer, delivering radiation in deep inspiration breath-hold condition can considerably reduce the dose to the surrounding normal structures, particularly heart and liver.

This PDF is available for free download from a site hosted by Medknow Publications (www.medknow.com).

### O-48

### Yttrium Aluminum Garnet (YAG) a New OSL phosphor for radiation dosimetry

NS Rawat, DR Mishra, MB Kakade*, S Ramanathan*, MS Kulkarni. *Radiation Safety Systems Division, *Material Science Division, Bhabha Atomic Research Centre, Mumbai - 400 085, India*

**Introduction:** Yttrium Aluminum Garnet (YAG) is a well-known phosphor material with a wide variety of applications. However, the dosimetric applications of YAG for medical dosimetry using optically stimulated luminescence (OSL) techniques have not been reported so far. The recent report of OSL properties of YAG exposed to beta radiation[1] has generated a lot of interest for its dosimetric properties in environmental and high-energy dosimetry. The present paper discusses the YAG as a new phosphor material for its possible use in the high-energy dose measurement based on OSL technique.

**Materials and Methods:** The crystalline YAG powder was synthesized using the nitrates-urea solution combustion reaction[2] and given a pre-irradiation annealing treatment at 1000°C for 1 hr to remove the thermal stress and impurities during its preparation. The YAG samples weighing 20 mg each were irradiated to 1.25 MeV photons using a $^{60}$Co teletheraphy machine (Theratron 780E) to an absorbed dose in the range of 1 Gy to 1 kGy using in a PTW water phantom at a depth of 5 cm. The samples were also exposed to high-energy X-ray photons (6 MV to 18 MV) for an absorbed dose of 1 and 2 Gy in a tissue equivalent phantom at a depth of 5 cm using linear accelerators (Varian Clinac 2100C and Clinac 2100 CD). The

Table 1: Absolute and normalized OSL response of YAG with respect to 1.25MeV $^{60}$Co for an absorbed dose of 2Gy

| *Energy (MV)* | *Integrated OSL counts* | *Normalized OSL counts* |
|---|---|---|
| 1.25MeV | 31001 | 1 |
| 6 | 31200 | 1.00642 |
| 10 | 30542 | 0.98519 |
| 15 | 30817 | 0.99406 |
| 18 | 31701 | 1.02258 |

YAG - Yttrium Aluminum Garnet

[Downloaded free from http://www.jmp.org.in on Friday, July 29, 2011, IP: 130.14.254.25] || Click here to download free Android application for t

OSL measurements were done using photon-counting module in a programmable OSL reader interfaced to PC through an RS-232 serial interface. The samples were stimulated using continuous wave (CW) OSL mode with a 470 nm stimulation wavelength (light intensity > *200 mW/cm²*).

**Results:** The OSL response of the YAG phosphor for the $^{60}$Co in the dose range of 1 Gy to 1 kGy is shown in Figure 1. The YAG is found to be a sensitive OSL phosphor having a linear dose response in the dose range upto to 1 kGy. The phosphor was also found to have an energy independent response for photons in the range of 1.25 MeV ($^{60}$Co g-rays) to 18 MV (X-rays) as evident from Figures 2 and 3. The normalized and absolute OSL response of YAG for variable photon energies for an absorbed dose of 2 Gy is shown in Table 1. Although the YAG is not tissue equivalent material, small size YAG dosimeters can be used for in-phantom dosimetry in the therapeutic dose range for the relative dose measurements. The YAG can be used for leakage dose measurements and for determining radiation levels around radiotherapy installations. Fast OSL readouts are possible based on the YAG phosphor. Post irradiation OSL fading of the YAG samples was found to be negligible for a period of one month.

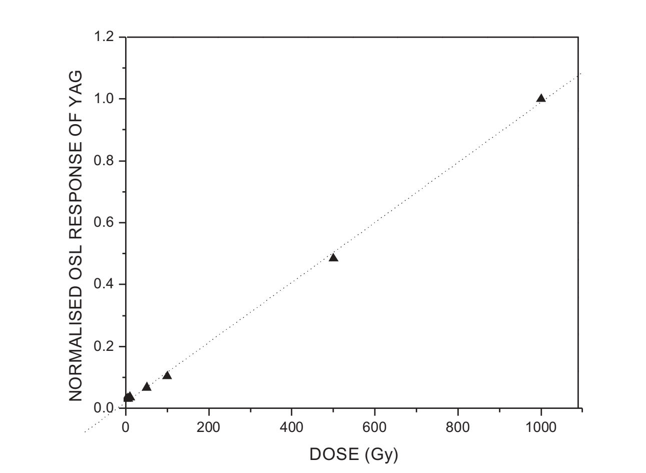

**Figure 1:** Dose linearity of YAG for $^{60}$Co

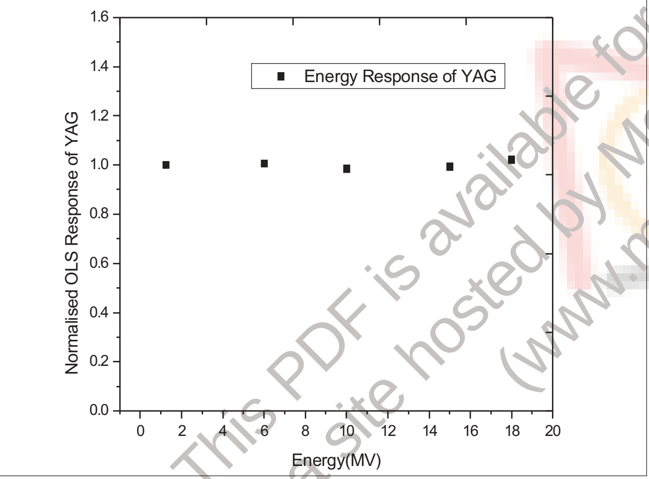

**Figure 2:** Photon energy response of YAG for an absorbed dose of 2Gy

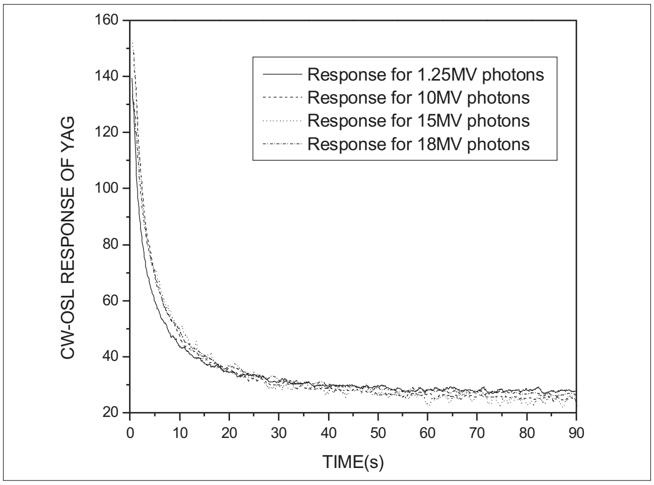

**Figure 3:** OSL curves of YAG corresponding to different photon energies

### References

1. De la Rosa E, Rodrìguez RA, Dìaz-Torres LA, Salas P, Meléndrez R, Baraboza-Flores M. Optically stimulated luminescence properties of nanocrystalline $Y_3Al_5O_{12}$ b radiation. Opt Mat 2005;27:1245-9.
2. Ramanathan S, Kakade MB, Roy SK, Kutty KK. Processing and characterization of combustion synthesized YAG powders. Ceramics Int 2003;29:477-84.

This PDF is available for free download from a site hosted by Medknow Publications (www.medknow.com).

### O-49

### A quantitative treatment planning study comparing radiograph and CT based multi-catheter interstitial intra operative implant dosimetry for accelerated partial breast irradiation using dose volume indices

Ritu Raj Upreti, Shamurailatpam Dayananda Sharma, Ashwini Budrukkar*, Anusheel Munshi*, Rakesh Jalali*, DD Deshpande.
*Department of Medical Physics, *Radiation Oncology, Tata Memorial Hospital, Mumbai - 400 012, India*
E-mail: rituraaj123@yahoo.com

**Introduction:** CT based dosimetry provides a clinically realistic evaluation of interstitial implant as compared to traditional orthogonal radiograph technique. In this study dosimetric outcome of radiograph and CT based planning of multi-catheter intestinal implant used for Accelerated Partial Breast Irradiation (APBI) were compared using different dose volume indices. The potential of interactive isodose optimization algorithm was tested for improved tumor coverage.

**Materials and Methods:** Intra-operative placement of flexible nylon tubes were performed on twelve consecutive patients of early stage breast cancer using the Paris system. For each patient an enface radiograph and axial CT images were taken and dosimetry was carried out on Plato Sunrise TPS. Lumpectomy cavity, CTV and ipsilateral breast were delineated on axial CT images following RTOG 0413 guideline. Catheter reconstruction was done on CT images. For each patient three plans were generated using active loading length measured from a) the radiograph (P1) and b) based on the CTV (P2). In both P1 and P2 geometrical optimization was done on volume. The reference prescription isodose of P2 was subsequently optimized interactively using graphical optimization tool and this yielded the plan P3. Dosimetric outcome were evaluated qualitatively and quantitatively using the dose volume indices which include coverage index (CI), external volume index (EI), relative dose homogeneity index (DHI), overdose volume index (OI) and conformal index (COIN).

**Results:** The median volume of lumpectomy cavity was 64.5 cc (range 32.7-141 cc), while the volume of CTV ranged from 77.5-257 cc (median 163 cc). The median (SD) CI of lumpectomy cavity estimated from P1, P2 and P3 was 0.82 (0.12), 0.83 (0.11) and 0.92 (0.07) respectively. The corresponding value for CTV was 0.70 (0.09), 0.74 (0.09) and 0.86 (0.08). Increase in the median CI of CTV, 23% (w.r.t. P1) and 16% (w.r.t. P2) observed in graphically optimized plan (P3) lead to decrease in median DHI from 0.82 to 0.73. In all patients $V_{150}$ and $V_{200}$ was lesser than 60 cc and 19 cc respectively; highest being observed in P3. Highest median OI of 0.09 (SD 0.03) was observed in P3 as compared to 0.04 of P1 and 0.05 of P2. EI was highest in P1 (median 0.23) as compared to 0.16 of P2 and 0.21 of P3. Significant improvement of COIN (median 0.71, SD 0.06) was observed in P3

[Downloaded free from http://www.jmp.org.in on Friday, July 29, 2011, IP: 130.14.254.25] || Click here to download free Android application for t over 0.51 and 0.60 of P1 and P2 respectively.

**Conclusion:** Dosimetry based on the active length measured from X-ray provides excessive irradiation of normal breast. Interactive graphical optimization allows the shaping of prescription isodose to the shape of target at the cost of high dose volume and dose inhomogeneity. Use of dose volume indices allow quantitative evaluation of different plans and can be use as a tool to correlate dosimetry with clinical outcome.

**O-50**

**Study of X-ray attenuation properties of indigenously developed shielding materials made of red mud and fly ash with barium compound**

Aarti R Kulkarni, Neeraj Dixit*, N Kadambini Devi, VK Shirva, SP Agarwal.
*Radiological Safety Division, Atomic Energy Regulatory Board, Mumbai, *Radiological Physics and Advisory Division, Bhabha Atomic Research Centre, Mumbai, India*

**Introduction:** Different structural shielding materials are used in the construction of X-ray installations and radiation facilities for the safety of radiation workers and general public. Currently concrete, brick and lead are used as structural shielding materials (brick is used only up to diagnostic X-ray energy range). We have studied the radiological properties of slabs of different samples of red mud and fly ash (developed with composition of barium compounds)[1] generated from aluminium industry waste. The red mud based shielding materials confirm to the requirement of compressive strength and impact strength as specified by Indian Standard for cementious shielding materials and ceramic tiles.[2] Red mud is the waste generated during aluminium production from bauxite. It is reported that production of 1 tonne of metallic aluminium generates about 2 tonnes of red mud. This paper presents the comparative study of X-ray attenuation properties of red mud based shielding materials with the conventionally used shielding materials such as concrete and lead. Methods: Attenuation characteristics of Red mud slabs of size 12 cm x 12 cm (of different compositions with barium compounds) and lead sheets were studied using Polydoros LX X-ray unit (total filtration 4 mm of Al). Unfors make solid state detector based (sealed silicon detector) dosemeter was used to measure the incident and transmitted dose ($I_0$ and $I_t$). Collimated X-ray beam of size 10 cm x 10 cm at target to dosemeter distance of 100 cm was used and the slabs were inserted between the target and dosemeter to measure the transmitted dose. Transmission measurements were carried out first for lead sheets of varying thickness and transmission curve was plotted. Similar measurements were carried out for each sample of red mud slab to find percentage transmission. Using transmission data of each sample, linear attenuation coefficient (μ) and half value thickness (HVT) are computed.

**Result and Conclusion:** The X-ray beam attenuation characteristics in terms of HVT for different red mud slabs at 100 kV energy of X-ray has been computed and compared with HVT's of conventional concrete and lead materials and are shown in Table 1. The HVT values of few red mud slabs are slightly higher than the HVT values of lead and HVT values of red mud slabs are significantly less than the HVT values of concrete for 100 kV X-rays which is used for medical diagnostic applications. Conventionally used lead is characterized by high toxicity in production and recycling and causes environmental pollution. Iron, barium and titanium are non-toxic and can be used effectively for shielding against X-rays as the next choice. The red mud generated in aluminium industry contains fairly high quantity of iron oxide, titanium oxide and aluminium oxide and hence composition of Red Mud with barium compound shows efficient shielding for X-rays. It appears from the measurements that the tested shielding materials can be used for the various shielding applications in diagnostic X-ray and CT-installations as well as for protective accessories used in radiation facilities.

Acknowledgement: The authors are thankful to Shri S.S. Amritphale and Shri Avneesh Anshul of Regional Research Laboratory, Bhopal for providing the red mud samples for this study.

**Reference**

1. Amritphale SS, Anshul A, Chandra N, Ramkrishnan N. A novel process for making radiopaque materials using bauxite-Red mud. J Eur Ceramic Soc 2006;10:1016/j.jeurceramsoc.2006.05.106.

**O-51**

**Current status of beta dosimetry for personnel monitoring TLD badge: Response for disc type and tape type cards based on $CaSO_4$: Dy teflon**

K Srivastava, G Varadharajan, S Chatterjee, AK Bakshi, RK Kher.
*Radiological Physics and Advisory Division, Bhabha Atomic Research Centre, CTCRS Building, Anushakti Nagar, Mumbai - 400 094, India*

Table 1: Linear attenuation coefficient and HVT values of RM based materials

| Sample | Density (gm/cc) | Thickness of the sample (cm) | % Trans-mission | Linear attenuation coefficient ($cm^{-1}$) | Half value thickness (cm) |
|---|---|---|---|---|---|
| RM+$BaCO_3$ (F) | 2.92 | 0.5 | 0.89 | 9.43 | 0.073 |
| RM+$BaSO_3$ (H) | 2.78 | 0.4 | 2.21 | 9.53 | 0.073 |
| RM+$BaSO_4$ (G) | 2.91 | 0.5 | 0.98 | 9.24 | 0.075 |
| $BaSi_2O_5$ (P) | 2.4 | 0.5 | 1.08 | 9.13 | 0.075 |
| RM+$BaSO_4$ (E) | 3.27 | 0.6 | 0.49 | 8.85 | 0.078 |
| G.RM.(green unsintered) | 3.4 | 0.7 | 0.25 | 8.56 | 0.08 |
| $BaTi_5O_{11}$(M) | 3.1 | 0.72 | 0.22 | 8.49 | 0.081 |
| $BaFe_4Ti_2O_{11}$(K) | 2.47 | 0.75 | 0.47 | 7.13 | 0.097 |
| $BaFe_2TiSi_2O_9$(Q) | 2.7 | 0.8 | 0.96 | 6.41 | 0.107 |
| $Ba_4Ti_{13}O_{30}$(R) | 2.4 | 0.9 | 0.37 | 6.19 | 0.11 |
| Fe+$BaCO_3$(I) | 2.15 | 0.9 | 0.45 | 6.0 | 0.12 |
| RF(raw fly ash) | 2.38 | 1.05 | 0.27 | 5.62 | 0.123 |
| RM+LDO(B) | 2.75 | 1.1 | 0.57 | 4.7 | 0.147 |
| $Ba_6Ti_3Fe_2O_{18}$(O) | 2.6 | 0.85 | 8.5 | 2.89 | 0.24 |
| RM (D) | 2.68 | 1.0 | 7.01 | 2.65 | 0.26 |
| RM+Fe-Pyrites(A) | 2.45 | 1.0 | 7.85 | 2.54 | 0.273 |
| FeTiO(U) | 2.6 | 0.85 | 8.65 | 2.93 | 0.32 |
| $TiO_2$(X) | 2.1 | 1.1 | 12.28 | 1.9 | 0.36 |
| Fe-Pyrites+LDO(C) | 1.46 | 1.5 | 31.81 | 0.76 | 0.907 |

HVT for lead: 0.045 cm (measured with 4 mm Al filtration), Standard HVT for concrete: 1.6 cm

This PDF is available for free download from a site hosted by Medknow Publications (www.medknow.com).

[Downloaded free from http://www.jmp.org.in on Friday, July 29, 2011, IP: 130.14.254.25] || Click here to download free Android application for t

Table 1: TL response of two types of cards for 5 mSv from various beta sources under different irradiation conditions. Relative response is given in the bracket.

| *Irradiation condition of dosimeter card* ↓ | *Sr/Y-90* | | *Kr-85* | | *Pm-147* | |
|---|---|---|---|---|---|---|
| *TL response (relative response)* → | *Disc card* | *Tape card* | *Disc card* | *Tape card* | *Disc card* | *Tape card* |
| Bare card | 5721 (1.00) | 5609 (1.0) | 1645 (1.00) | 2433 (1.00) | 1128 (1.00) | 195 (1.00) |
| Card in paper wrapper | 5696 (1.00) | 5649 (1.0) | 1230 (0.75) | 1670 (0.69) | 282 (0.25) | 195 (1.00) |
| Card in paper wrapper and polythene pouch | 5380 (0.94) | 5377 (0.96) | 935 (0.57) | 1377 (0.57) | 157 (0.14) | 101 (0.52) |
| Card in cassette (D3 reading) | 5256 (0.92) | 5462 (0.97) | 813 (0.49) | 1569 (0.64) | 112 (0.10) | 127 (0.65) |

Table 2: Disc ratios for both type of cards (Disc card and Tape Car) for two beta sources

| *Beta source* | *Disc card* | | | *Tape card* | | |
|---|---|---|---|---|---|---|
| | D2/D1 | D3/D2 | D3/D1 | D2/D1 | D3/D2 | D3/D1 |
| Sr/Y-90 | 46.5 | 1.72 | 80.04 | 133.7 | 1.7 | 230.2 |
| Kr-85 | 1.4 | 237.14 | 332.0 | 0.8 | 78.5 | 61.9 |

**Introduction:** Accurate measurement of beta radiation is important in personnel monitoring as significant number of workers receive beta doses specially those from the nuclear industries and research organizations. However, thermoluminesce dosimetric systems gives highly energy dependent response for beta radiation. Several methods have been proposed to minimize the beta energy dependence and improve the dose estimation of beta doses. An ideal beta dosemeter will be ultra-thin TL dosemeter made up of tissue equivalent material such as LiF covered by ~ 5 mg.cm$^{-2}$ thick tissue equivalent filter to measure Hp(0.07), which is an internationally accepted quantity for beta dose reporting (ICRU 47). However, ultra-thin TL dosemeters have certain limitations as they have high detection threshold, require very careful handling during readout, high quality control at production level for sensitivity variation and sophisticated instrumentation. In India, presently a three element $CaSO_4$: Dy Teflon disc (0.8 mm or 200 mg. cm$^{-2}$ thick) dosimeter is being used for photon as well as beta dosimetry since 1978.[1] The Badge is worn at either chest level or wrist/head level depending upon the working condition. Each Disc is prepared by cold pressing an individually weighted fixed mass of uniformly mixed $CaSO_4$: Dy and Teflon in ratio of 1:3. In order to make the preparation procedure less cumbersome and to take advantage of thin dosemeter, a skived tape type dosemeter was developed in 1992.[2] A single tape element (51 mm x 17 mm x 0.4 mm i.e., 80 mg. cm$^{-2}$) was used and an anti buckling device (mica strip 25 mg.cm$^{-2}$) was provided to avoid any buckling during heating. The readout time for each element is 30 sec for tape card compared to the 60 s needed for disc type card and this gives advantage in large scale monitoring. This paper gives the response of the two types of personnel dosemeters for ISO recommended new beta calibration sources (ISO 6980, 1997).

**Materials and Methods:** For the present study two type of personnel monitoring badges (three loose disc type and single tape element type dosemeter) were used to measure their response in terms of Hp(0.07). A secondary standard Beta Irradiation system BSS-2 was used for beta irradiations having three sources namely:- Sr /Y-90, Kr- 85 and Pm-147, which meets the ISO recommendations of standard beta sources. The dose rate of the sources provided by the Psysikalische-Techniche Bundesanstalt (PTB) are directly traceable to primary standard for the dose of beta radiation in tissue. Beta irradiations were carried out at distance of 30 cm for Sr/Y-90 and Kr-85 sources and at 20 cm for Pm-147 source. All irradiations were carried out with dosemeters attached on front surface of rectangular Perspex phantom (30 x 30 x 5 cm$^3$) at normal incidence. Appropriate beam flattening filters were used for all the sources as per the recommendation of ISO. For reader calibration purpose, both types of TLD cards were subjected to 5 mSv of Cs-137 gamma source. Irradiations were carried in four different conditions for both type of cards - i) bare card, ii) under paper wrapper, iii) under paper wrapper and polythene pouch and iv) loaded into TLD cassette. For each point in the Table, average reading of at least six cards was taken.

**Result and Discussion:** Table 1 gives the TL response and relative response (normalized against the response of bare card) for two types of TLD cards for different irradiation geometries using all the three beta sources. It is evident that both TLD systems are not suitable for measurement from Pm-147 source, as TL readings are in the range of background counts and lack any pattern to enable the dose estimation. However, It was found that when tape card was exposed with tape side facing the source, TL counts were more by factor of 1.01, 1.19 and 3.85 for Sr-Y-90, Kr-85 and Pm-147 respectively. Thus the presence of mica sheet as anti buckling device deprived the advantage of thinner dosemeter. Table 2 gives the Disc Ratios under different filter regions for Sr/Y-90 and Kr-85 source whereas for Pm-147 these ratios are not significant and hence not provided.

**Conclusion:** This study was undertaken as base work for the development of thin dosemeters to cover wide beta energy range. It is evident that present system is not suitable for beta energies less than 0.5 MeV. In order to cover the lower beta energies and improve the accuracy in dose estimation it is necessary to develop of thin dosemeter.

This PDF is available for free download from a site hosted by Medknow Publications (www.medknow.com)

**References**

1. Vohra KG, Bhatt RC, Bhuwan C, Pradhan AS, Lakshmanan AR, Shastry SS. A personnel dosimetry TLD badge based on $CaSO_4$: Dy Teflon TLD discs. Health Physics 1980;38:193-6.
2. Nagpal JS, Popli KL, Kher RK, Varadharajan G. Development of quality assurance procedures for Thermoluminescent $CaSO_4$: Dy teflon skived tape dosemeter. Radiat Protect Dosim 1992;40: 45-8.

**O-52**

**Film scanning generated planer dose profile and its usefulness in point off-axis dose calculations**

Shukla Rahul, Sachdeva Jaineet, MK Mahajan. *Christian Medical College and Hospital, Ludhiana, Punjab, India*

**Introduction:** Percentage depth dose data are generally generated for the central axis depth dose at various fields. Clarkson method is used to calculate depth dose distribution at any point within the field or outside the field but calculation is not simple; alternatively Day's method[1] can be used to calculate for rectangular field. In this method, percentage depth dose can be calculated at any point within the medium. Day's method can be extended further for determining dose distribution outside the field limit.

**Materials and Methods:** For the calculation purpose by applying Day's method, we require:

| | | |
|---|---|---|
| 1. | Output of teletherapy unit in air | Obtained by using SSD dosimeter |
| 2. | Off axis ratio across ($K_Q$) the central axis | Obtained by film scanning |
| 3. | Back scatter factor for other | Obtained from BJR |

[Downloaded free from http://www.jmp.org.in on Friday, July 29, 2011, IP: 130.14.254.25] || Click here to download free Android application for t

Table 1: Data generated by film scanning

| | | | | | | | | | | | | | | |
|---|---|---|---|---|---|---|---|---|---|---|---|---|---|---|
| 0.84 | 0.86 | 0.88 | 0.89 | 0.89 | 0.89 | 0.9 | 0.89 | 0.89 | 0.9 | 0.89 | 0.88 | 0.87 | 0.86 | 0.83 |
| 0.85 | 0.87 | 0.89 | 0.9 | 0.9 | 0.89 | 0.9 | 0.9 | 0.91 | 0.91 | 0.9 | 0.89 | 0.89 | 0.87 | 0.84 |
| 0.86 | 0.88 | 0.89 | 0.9 | 0.92 | 0.93 | 0.93 | 0.94 | 0.93 | 0.93 | 0.92 | 0.92 | 0.9 | 0.88 | 0.87 |
| 0.88 | 0.9 | 0.91 | 0.94 | 0.95 | 0.95 | 0.96 | 0.95 | 0.95 | 0.95 | 0.93 | 0.93 | 0.91 | 0.89 | 0.88 |
| 0.88 | 0.9 | 0.92 | 0.93 | 0.95 | 0.97 | 0.96 | 0.96 | 0.96 | 0.95 | 0.94 | 0.93 | 0.91 | 0.89 | 0.88 |
| 0.89 | 0.91 | 0.94 | 0.95 | 0.96 | 0.97 | 0.98 | 0.98 | 0.97 | 0.97 | 0.97 | 0.95 | 0.93 | 0.91 | 0.89 |
| 0.9 | 0.91 | 0.96 | 0.97 | 0.96 | 0.98 | 0.98 | 0.99 | 0.99 | 0.98 | 0.97 | 0.95 | 0.94 | 0.92 | 0.89 |
| 0.89 | 0.92 | 0.95 | 0.96 | 0.97 | 0.98 | 0.98 | 1 | 0.98 | 0.98 | 0.96 | 0.95 | 0.94 | 0.92 | 0.9 |
| 0.89 | 0.92 | 0.94 | 0.96 | 0.97 | 0.98 | 0.98 | 0.99 | 0.98 | 0.98 | 0.97 | 0.96 | 0.94 | 0.91 | 0.9 |
| 0.89 | 0.91 | 0.93 | 0.96 | 0.97 | 0.98 | 0.99 | 0.99 | 0.98 | 0.98 | 0.96 | 0.96 | 0.94 | 0.91 | 0.89 |
| 0.88 | 0.9 | 0.93 | 0.95 | 0.97 | 0.97 | 0.98 | 0.98 | 0.97 | 0.96 | 0.96 | 0.95 | 0.93 | 0.92 | 0.89 |
| 0.88 | 0.9 | 0.92 | 0.94 | 0.95 | 0.96 | 0.97 | 0.97 | 0.97 | 0.96 | 0.95 | 0.94 | 0.93 | 0.91 | 0.88 |
| 0.87 | 0.89 | 0.91 | 0.93 | 0.93 | 0.95 | 0.95 | 0.95 | 0.95 | 0.95 | 0.94 | 0.93 | 0.91 | 0.89 | 0.88 |
| 0.86 | 0.88 | 0.89 | 0.91 | 0.91 | 0.92 | 0.93 | 0.93 | 0.93 | 0.93 | 0.93 | 0.91 | 0.89 | 0.88 | 0.86 |
| 0.85 | 0.86 | 0.89 | 0.92 | 0.91 | 0.93 | 0.91 | 0.91 | 0.91 | 0.91 | 0.9 | 0.89 | 0.88 | 0.87 | 0.84 |

Table 2: Data generated by Secondary Standard Dosimeter

| | | | | |
|---|---|---|---|---|
| | | 0.97 | | |
| | | 0.99 | | |
| 0.95 | 0.98 | 1.00 | 1.00 | 0.98 |
| | | 1.00 | | |
| | | 0.95 | | |

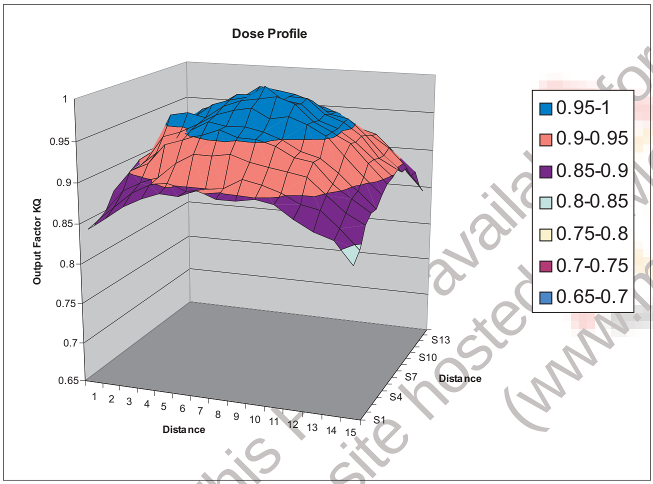

Dose profile is generated by using data of Table 1

| | | |
|---|---|---|
| | field sizes | |
| 4. | Percentage depth dose data along central axis | Obtained from BJR |

**Results:** We have exposed the film for 10 cGy and then we have divided whole film into sector of 1.7 cm, measurements has been taken at the middle of each sector. If we compare the highlighted points of Table 1 with values shown in Table 2, we can see that film scanning data shows better variation in output compared to the values obtained by Secondary Standard dosimeters for the same distances.

**Discussions:** Clarkson's technique is not practical for manual calculation. Day's method is found simple for manual calculation. $K_Q$ factor, which is simply ratio of the output at any point within the field to the output at the centre, also $K_Q$ is determined in air for the primary beam. Once it is generated rest of the calculation is simple and found useful for percentage depth dose which can be calculated at any point within the medium or outside the field limit by using Day's method.

**Conclusions:**

i. The values of $K_Q$ found from minimum 0.8327 to 1 maximum for 30 x 30 field size and are reproducible on other machines too because of ratio.

ii. These values are checked by using calibrated secondary standard dosimeter at few points, highlighted in Table 1.

iii. If primary beam profile is flat (not in case of Co-60 beam) then off axis ratio ($K_Q$) = 1 but PDD at off axis point will be less than that from center, this is because of reduced side scatter at that point.

iv. Data generated by film scanning shows point to point variation, but by using SSD shows no change in the region of 5 $cm^2$ near the centre.

This PDF is available for free download from a site hosted by Medknow Publications (www.medknow.com).

### References

1. Khan FM. The Physics of Radiation Therapy. 3rd ed.
2. Leung PM. The Physical Basis of Radiotherapy. Revised ed.

### O-53
### Monte Carlo calculations of different dose parameters for photon sources relevant in brachytherapy

T Palani Selvam, RS Vishwakarma, S Kannan. *Radiological Physics and Advisory Division, Bhabha Atomic Research Center, CT and CRS Building, Anushaktinagar, Mumbai - 400 094, India*
E-mail: vishwakarma_ram@yahoo.com

**Objective:** Monte Carlo-based codes such as EGSnrc, GEANT4, MCNP4A, 4B and 5C are widely employed in modeling of brachytherapy photon sources. These codes include transport of secondary electrons. However, there are many research institutions still use MCNP version 3.1 (MCNP3.1) which ignores transport of electrons. In the present study, a major investigation is attempted to verify how safely MCNP version 3.1 can be used for brachytherapy dose calculations. Towards this end, we repeated many published studies using MCNP3.1.

**Materials and Methods:** Monte Carlo calculations: Point source-based dosimetry data are still in use for realizing dose distributions in water around cylindrical brachy photon sources. Methods such as quantization and Sievert intergral make use of these data. Dose rate to water at radial distance, r in water medium due to a point photon source in water can be calculated from the following relation:

$$D(r) = S_k \left[\frac{\mu_{en}}{\rho}\right]_{air}^{wat} \frac{f(r)}{r^2} \qquad (1)$$

where $S_k$ is air-kerma strength of point photon source expressed in units of $cGycm^2h^{-1}$, $\left[\frac{\mu_{en}}{\rho}\right]_{air}^{wat}$ is the ratio mass-energy-absorption coefficient of water to that of air and f (r) is dose ratio function that accounts for combined effect of attenuation and energy absorption build-up factor in water. The ratio of water-kerma in water, $K_{w,w}(r)$ to water-kerma in free space, $K_{w,f}(r)$ gives f (r). The latest published study is calculation of point source-based radial dose function, g(r) and reference dose-rate in water (dose-rate at 1 cm) in a 50-cm radius water for $^{137}Cs$, $^{192}Ir$, $^{169}Yb$, $^{125}I$ and $^{103}Pd$ sources phantom using GEANT4 and MCNP5

[Downloaded free from http://www.jmp.org.in on Friday, July 29, 2011, IP: 130.14.254.25] || Click here to download free Android application for t

**Table 1: Comparison of dose ratio function, f(r), radial dose function, g(r) and values of reference dose rate, $D(r_0)$ for different point photon sources. The values of f(r) are normalized at 1 cm.**

*Dose ratio function, f (r) (normalized at 1 cm)*

| Distance r (cm) | $^{192}$Ir Messenger et al Radiology, Vol 90,1968 | $^{192}$Ir This work | $^{125}$I Angelopoulos et al Phys. Med. Biol, Vol. 36, 1991 Phantom radius = 10 cm | $^{125}$I This work |
|---|---|---|---|---|
| 0.5 | 0.998 | 0.995 | 1.017 | 1.014 |
| 1 | 1.000 | 1.000 | 1.000 | 1.000 |
| 2 | 1.001 | 1.007 | 0.888 | 0.888 |
| 5 | 0.989 | 1.001 | 0.440 | 0.467 |
| 7 | 0.967 | 0.978 | 0.252 | 0.277 |
| 10 | 0.910 | 0.905 | - | 0.121 |

| | *$^{137}$Cs Meisberger et al Radiology, Vol. 90,1968* | *$^{137}$Cs This work* | *$^{60}$Co Sakelliou et al Phys. Med. Biol. Vol. 37, 1992 Phantom radius = 10 cm* | *$^{60}$Co This work* |
|---|---|---|---|---|
| 0.5 | 1.005 | 1.004 | 1.007 | 1.008 |
| 1 | 1.000 | 1.000 | 1.000 | 1.000 |
| 2 | 0.989 | 0.990 | 0.985 | 0.981 |
| 5 | 0.952 | 0.951 | 0.935 | 0.934 |
| 7 | 0.919 | 0.915 | 0.897 | 0.897 |
| 10 | 0.855 | 0.846 | 0.833 | 0.834 |

*Radial dose function, g(r)*

| *r (cm)* | *Melhus et al, Med. Phys, Vol. 33, 2006* $^{137}$Cs | *Melhus et al, Med. Phys, Vol. 33, 2006* $^{192}$Ir | *This work* $^{137}$Cs | *This work* $^{192}$Ir |
|---|---|---|---|---|
| 0.5 | 1.004 | 0.994 | 1.003 | 0.995 |
| 1 | 1.000 | 1.000 | 1.000 | 1.000 |
| 2 | 0.990 | 1.009 | 0.990 | 1.008 |
| 5 | 0.951 | 1.013 | 0.953 | 1.013 |
| 7 | 0.920 | 0.997 | 0.923 | 1.002 |
| 10 | 0.866 | 0.949 | 0.870 | 0.961 |

| *Source* | *Reference dose rate ($cGyh^{-1}mCi^{-1}$)* *Melhus et al, Med. Phys, Vol. 33, 2006* | *This work* |
|---|---|---|
| $^{137}$Cs | 3.153 | 3.137 |
| $^{192}$Ir | 4.541 | 4.548 |
| $^{169}$Yb | 1.996 | 2.000 |
| $^{125}$I | 1.321 | 1.368 |
| $^{103}$Pd | 0.910 | 0.959 |

This PDF is available for free download from a site hosted by Medknow Publications (www.medknow.com).

codes (Melhus et al 2006). We repeated this work as well.

**Results and Conclusion:** All our dose results are based on F4 tally type using latest mass-energy-absorption coefficient $\mu_{en}/\rho$ data (Hubbell and Seltzer 1995). Although we have data for all sources investigated, Table 1 compares the values of f(r) (normalized at 1 cm), g(r) and reference dose rate (dose rate per mCi at 1 cm in water) for select sources and distances against the published values. An analysis of f(r) values suggests that our values are in good agreement with the published values for smaller distances. The disagreement at larger distances is attributed to phantom dimensions. For example, Angelopoulos *et al* (1991) used a 10 cm radius water phantom whereas in our calculations we used a 16 cm radius phantom. A comparison of Melhus *et al's* g(r) and $D(r_0)$ values with our data showed reasonable agreement for sources $^{169}$Yb, $^{192}$Ir and $^{137}$Cs. However, for low-energy sources, $^{125}$I (mean energy 30 keV) and $^{103}$Pd (mean energy 20 keV) our values resulted in overestimation. This may be attributed to the following reasons. Recent studies have shown that the photon cross-section library should be updated for low energy photons (10-60 keV) for low Z materials as NIST (Hubbell and Seltzer) has revised the cross-section data of total linear attenuation coefficient, $\mu$ and photoelectric effect. Our auxiliary Monte Carlo simulations showed that MCNP3.1-based $\mu$ values for water for low energy photons are lower by few percent when compared to the values reported by NIST. Another possible discrepancy with Melhus *et al's* work is the use of F6 tally type, which directly calculates energy absorbed per unit mass (MeV/gm) in a medium. This tally type uses built-in $\mu_{en}/\rho$ data. Our investigation in this regard shows that the built-in $\mu_{en}/\rho$ values of water used in MCNP version 3.1 code are significantly lower than that of latest values. At present we have no knowledge about MCNP5's built-in $\mu_{en}/\rho$ data. It is concluded that MCNP3.1 can safely be used for sources such as $^{192}$Ir, $^{169}$Yb, $^{137}$Cs and $^{60}$Co. However, for modeling $^{125}$I and $^{103}$Pd sources the photon cross-section data should be updated. Care should be taken while using F6 tally type.

### O-54

### Verification of treatment planning system parameters in tomotherapy using EBT radiochromic film

EB Rajmohan, Pratik Kumar, Bhudatt Paliwal#, David Westerly#, N Gopishankar*, RK Bisht*, D Tewatia#, KS Jothi Basu**, GK Rath**.
*Department of Medical Physics Unit, BRAIRCH, *Department of Neurosurgery, **Department of Radiotherapy, All India Institute of Medical Sciences, New Delhi - 110 029, India, #Department of Human Oncology, University of Wisconsin, Highland Avenue Madison, WI 53792-0001*

**Introduction:** To verify the planning parameters and dose distribution calculated with the tomotherapy treatment planning system for a

[Downloaded free from http://www.jmp.org.in on Friday, July 29, 2011, IP: 130.14.254.25] || Click here to download free Android application for t

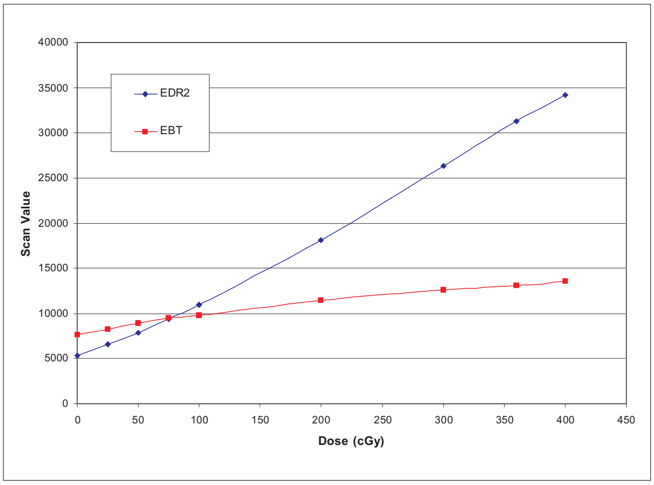

**Figure 1:** Film response vs. dose prostate patient using external beam therapy (EBT) radiochromic film. The recent development of intensity modulated radiotherapy (IMRT) using a linear accelerator (linac) offers a major advancement in radiation therapy by allowing for more conformal dose distributions to be delivered to target volumes; thereby allowing for greater sparing of normal tissues. Tomotherapy represents the next step in IMRT treatments. Tomotherapy, which literally means "slice therapy", uses a megavoltage linac mounted on a CT-like ring gantry which rotates and delivers fan beam of radiation as a patient is translated on a treatment couch through the bore of the machine. Intensity modulation is achieved using a binary multi-leaf collimator. In addition, a bank of megavoltage-CT (MVCT) detectors is located on the gantry, opposite the linac, to provide onboard MVCT imaging capabilities. While both conventional IMRT and Tomotherapy treatments offer significant advantages in terms of the ability to shape delivered dose distributions to the target, they do so at the cost of increasing the complexity of treatment delivery. This increased complexity in turn requires more rigorous and precise methods of quality assurance to ensure that the dose distribution delivered by the machine agrees with the distribution calculated at the time of planning. Current methods for the verification of the dose delivery of the tomotherapy involve delivering the treatment beams from an accepted patient plan to a solid-water phantom containing radiographic film(s). The use of radiographic films however presents various difficulties, including a strong deviation from tissue equivalence, cost escalation and time-consuming film processing. In order to avoid these difficulties, this study seeks to asses the potential of EBT radochromic film for the verification tomotherapy dose delivery.

**Materials and Methods:** Both EDR2 and EBT films were characterized by their response to a series of nine known dose values ranging from 0-400 cGy delivered with a Varian Clinac 2300 linear accelerator. The treatment plan for single patient, previously treated for prostate cancer with Tomotherapy, was selected and the treatment beams from the selected plan were applied to a cylindrical solid-water phantom, allowing for the dose to solid-water to be calculated. The treatment was then delivered a total of four times, twice with EDR2 films placed within the phantom and twice with EBT films. For each type of film, treatments were delivered with the films oriented in first the coronal and then the saggittal plane. Additional measurements were made during each treatment delivery using an A1SL ion chamber. The ion-chamber measurements were used for both point dose comparisons as well as a means of scaling the relative film values to an absolute dose measurement. Following treatment delivery, films were allowed to wait overnight before being developed and then scanned using a Vidar VXR-16 Dosimetry-Pro Film Scanner. The scanned films were then transferred to the Tomotherapy treatment planning system for analysis.

**Results and conclusions:** Film response curves for both EDR2 and EBT films are shown in Figure 1. This figure clearly shows the sensitivity of the EBT film to be considerably lower across the entire measured dose range as compared with the radiographic films. The iso-dose curves and film profiles taken with both EDR2 and EBT films in the coronal plane. This shows good agreement between the calculated and measured dose distributions in the high dose region using both films; however, the EBT film shows a large discrepancy in the low-dose region due to the reason that radiochromic films are sensitive and has a better peak of absorption in the range of 600 to 670+10 nm range of light. In addition to the iso-dose curves and film profiles, the Tomotherapy treatment planning software also provides a means for calculating a distance to agreement parameter, gamma. Gamma maps for both EDR2 and EBT films taken in the coronal plane are also calculated. The low dose discrepancy is likely also a result of low sensitivity. The low sensitivity required that large scaling factors (based on ion-chamber measurements made in the high dose region) be applied from this study the low sensitivity of EBT, the shape of the response curve for the EBT and scaling to ion-chamber readings taken in the high dose region rectifies the problem in the high dose regions, but fails to do the same for the low dose regions.

### O-55

### Study of response of personnel monitoring TLD badge for high energy photons prevalent in medical accelerators

Munish Kumar, G Sahni*, LC Prasad, Kanta Chhokra*, Reena P Devi[#], RK Kher. *Radiological Physics and Advisory Division, Bhabha Atomic Research Center, Mumbai - 85, *Radiological Safety Division, Atomic Energy Regulatory Board, Mumbai - 94, [#]Advaced Centre for Treatment, Research and Education for Cancer (ACTREC), Kharghar, Navi Mumbai, India*

This PDF is available for free download from a site hosted by Medknow Publications (www.medknow.com)

Table 1: Measurement data at 6MV photon energies

| *Depth (mm)* | *Dose measured using ppc-40 (cGy)* | *TLD Reading (cGy)* | | | *Readout pattern* | *Dose (cGy)* |
|---|---|---|---|---|---|---|
| | | *D3* | *D2* | *D1* | | |
| 0 | 7.31 | 7.47 | 10.77 | 14.25 | γ-β | 7.47-5.90 |
| 5 | 14.13 | 13.75 | 13.84 | 14.34 | γ | 13.75 |
| 10 | 15.63 | 14.15 | 15.87 | 15.28 | γ | 14.15 |
| 15 | 15.89 | 14.86 | 15.67 | 14.91 | γ | 14.86 |
| 20 | 15.83 | 14.75 | 15.79 | 15.30 | γ | 14.75 |
| 50 | 15.11 | 15.17 | 15.60 | 14.53 | γ | 14.53 |
| 100 | 13.29 | 13.75 | 14.23 | 13.51 | γ | 13.51 |
| 150 | 11.32 | 11.23 | 12.25 | 11.09 | g | 11.09 |

Table 2: Measurement data at 18MV photon energies

| *Depth (mm)* | *Dose measured using ppc-40 (cGy)* | *TLD reading (cGy)* | | | *Readout Pattern* | *Dose (cGy)* |
|---|---|---|---|---|---|---|
| | | *D3* | *D2* | *D1* | | |
| 0 | 5.03 | 4.66 | 7.07 | 15.48 | γ-β | 4.35-23.55 |
| 5 | 10.52 | 10.88 | 12.43 | 17.49 | γ-β | 10.87-8.35 |
| 10 | 13.14 | 11.66 | 13.66 | 18.03 | γ-β | 11.65-7.20 |
| 15 | 14.47 | 13.26 | 13.95 | 17.56 | γ-β | 13.26 |
| 20 | 15.08 | 14.28 | 15.39 | 17.70 | γ | 14.28 |
| 25 | 15.47 | 15.76 | 15.67 | 17.43 | γ | 15.76 |
| 30 | 15.59 | 16.28 | 16.41 | 18.55 | γ | 15.59 |
| 35 | 15.55 | 14.70 | 15.56 | 17.56 | γ | 14.70 |
| 45 | 15.37 | 14.89 | 15.73 | 18.01 | γ | 14.89 |
| 50 | 15.10 | 18.83 | 19.18 | 21.95 | γ | 18.83 |
| 100 | 14.83 | 16.42 | 17.48 | 19.53 | γ | 14.83 |
| 150 | 13.39 | 14.91 | 15.86 | 17.36 | γ | 14.91 |

[Downloaded free from http://www.jmp.org.in on Friday, July 29, 2011, IP: 130.14.254.25] || Click here to download free Android application for t

Corresponding Author: Dr. Munish Kumar,
E-mail: munish_pms@rediffmail.com

**Introduction:** With the drastic increase and use of high-energy radiation sources in various fields such as medical, industrial and research applications, the accurate measurement of radiation doses is a difficult and challenging task. In India, nearly 80 medical linear accelerators are in operation for radiation therapy and produce photon beam energies from 4 MV to 18 MV. All these facilities are covered under personnel monitoring services. However, there are reports indicating that the most of the radiation detectors used worldwide exhibit under response at high photon energies and the errors in measurement of radiation dose can be high enough if not corrected. In view of above consideration, the present study was carried out to investigate the response/behavior of the personnel monitoring TLD badge used in our countrywide personnel monitoring programme.

**Materials and Methods:** The TLD badge used in this study is same as is used in our countrywide personnel monitoring programme. For this study, the TLD cards having spread in sensitivity less than 5% were used. The measurements were carried out with siemens primus high medical linear accelerator having dual photon beam energies (6 and 18 MV). The TLD badges were kept at phantom (virtual water phantom) and were exposed to three different doses at 7.31, 12.18, 17.05 cGy and 5.03, 8.39 and 11.74 cGy for 6 and 18 MV photon beam energies respectively. The surface doses were measured with well calibrated parallel plate chamber ppc-40 and 0.6cc thimble type ionization chamber with electrometer supplied by scanditronix. For each measurement two TLD badges were used. This study was carried out at the surface of the phantom as well as with different buildup thicknesses. For 6MV photon beam, the exposures were carried out at surface of the solid phantom (30x30x17 $cm^3$) and with various buildup thicknesses 5, 10, 15 and 20 mm. Also for 18MV photon beam, exposures were carried out at surface and various buildup thicknesses vary from 5, 10, 15, 20, 25, 30, 35 and 45 mm. The buildup material used for the purpose was virtual water (tissue equivalent material) slab of different thicknesses. The exposed TLD's were read on a well-calibrated automatic hot gas TLD badge reader.

**Result and Discussion:** It is well established that with the increase of photon energy, the depth at which maximum dose is delivered increases from almost 15 mm for 6MV photon beam energy to almost 30 mm for 18MV photon beam energy and this fact is confirmed with ionization chamber as well as with the TLD badge. It has also been found that the TLD badge worn by a radiation worker in high-energy field can sometimes lead to a misleading radiation patterns. This is likely to be encountered with those TLD badges, which are used to measure and distinguish mixed radiations such as $\beta$ and $\gamma$ radiations. Hence proper precautions must be taken while processing such dosimeters and also during dose estimation. However such alarming situations are less likely to exist, as the radiation worker is never exposed to the direct beam. Further it has been found that at surface, the D1/D3=2.0 for 6 MV photons whereas for 18 MV photons D1/D3=3.3 and is the artifact of the use of filters, used to distinguish and measure the radiation doses in mixed fields of beta and gamma radiation. However, with the increase in buildup thickness, the ratio of D1/D3 decreases. Various readout patterns and estimated doses for 6 and 18 MV photos are shown in Tables 1 and 2 respectively.

**Conclusions:** The dosimetry for high-energy photons needs a comprehensive study so that proper normalization and weighting factors can be proposed for the measurement of equivalent doses. Further studies are planned in near future.

### O-56
### Influence of room-scatter on dose to patient and staff in a pet scanner room: A Monte Carlo study

Rupali Rohatgi, K Biju, T Palani Selvam, S Kannan. *Radiological Physics and Advisory Division, Bhabha Atomic Research Centre, Trombay, Mumbai - 94, India*
E-mail: rupalirohatgi@rediffmail.com

**Introduction:** Positron Emission Tomography (PET) is the latest diagnostic modality in Nuclear Medicine. It provides vital physiological information in the field of oncology, neurology and cardiology. PET uses radioisotopes, which are cyclotron produced. $^{18}F$, a commonly used PET isotope is a positron emitter producing annihilation photons of 511 KeV. It is injected into the patient for diagnosis of malignancies. Here, the patient is an active source and spends around 45 minutes in post-administration waiting area and half an hour in a PET scanner room. Hence, it is necessary to provide adequate structural shielding for these facilities in order to keep the radiation doses to the occupational workers and the public within the permissible levels. The staff assisting the patient, receives both direct dose from the patient and scatter dose from the walls, floor and ceiling (room components). The patient also receives this scattered dose. Hence, this paper presents and discusses the percentage of scatter dose, at various locations in a scanner room.

**Materials and Methods:** Monte Carlo computations were done using the MCNP code. A PET scanner room of dimension 8 m x 4 m x 3 m having 15 cm thick concrete walls with 2.5 mm lead lining on door and lead glass equivalence viewing window was simulated as shown in Figure 1. A cylindrical water phantom at a diatance of 1 m from the floor with dimensions of 70 cm length and 30 cm diameter having uniformly distributed 1 mCi source emitting 0.511MeV photons was modeled. The transport of the positrons is not considered in this work because the range of the positron of energy 634 keV is much less than the dimension of the phantom. Track length and point detector tallies were used to score the photon

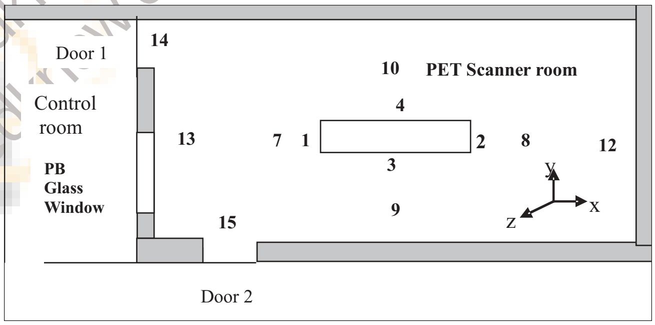

**Figure 1:** Cross-sectional view of a standard PET facility simulated using MCNP.

**Table 1:** Contribution of room-scatter at the different detector locations

| *Location no.* | *Detector location from phantom* | *% scatter* |
|---|---|---|
| 1 | At 5 cm | 6.27E-01 |
| 2 | At 5 cm | 9.37E-01 |
| 3 | At 5 cm | 4.15E-01 |
| 4 | At 5 cm | 3.49E-01 |
| 5* | At 5 cm above | 5.30E-01 |
| 6* | At 5 cm below | 6.26E-01 |
| 7 | At 1 m | 1.66E+01 |
| 8 | At 1 m | 1.70E+01 |
| 9 | At 1 m | 9.46E+00 |
| 10 | At 1 m | 9.57E+00 |
| 11* | At 1 m above | 7.88E+00 |
| 12 | At 4 m | 9.52E+01 |
| 13 | At 4 m | 1.04E+02 |
| 14 | Door 1 | 5.81E+01 |
| 15 | Door 2 | 4.61E+01 |

*: Locations 5,6 and11 on the z-axis are not shown in the Figure 1, The floor and ceiling lie on the z-axis.

This PDF is available for free download from a site hosted by Medknow Publications (www.medknow.com)

[Downloaded free from http://www.jmp.org.in on Friday, July 29, 2011, IP: 130.14.254.25] || Click here to download free Android application for t energy fluence spectrum at various locations. 1 million photon histories were followed. Using energy fluence spectrum, air-kerma rates were estimated around the phantom and the statistical errors are within 2%. In order to study the influence of walls, floor and ceiling, the calculation were repeated without these components. Comparison of air kerma values at each detector location with and without the room components gives an estimate of scatter contribution from walls, floor and ceiling.

**Results and Conclusion:** The percentage of room-scatter with respect to direct contribution from the patient is presented in the Table 1 for locations marked around the phantom as shown in Figure 1. The locations 1-6 are at 5 cm from the phantom. The Table shows that the scattered air kerma rate around the patient is only 0.6%. However, the dose to the patient is unaltered by the room-scattered photons. At 1 m depending upon the detector location the scatter varies from 9-16% of the direct air-kerma component from the patient. Near to walls the scatter contribution is almost 100% (see detector location 12 and 13). It is observed that the percentage room-scatter increases as the direct air-kerma rate component from the patient decreases due to dominance of the inverse square law. There may be an increase of scatter component as the area of the room decreases. From the results, it is seen that the technologist standing near the wall receives considerable amount of dose from scatter component scattered in comparison to the direct dose from patient. The study concludes that a PET room should have an adequate area to minimize the scatter to the staff involved.

### O-57
### Computer controlled multileaf collimator for Telecobalt machine

Rabi Raja Singh I, L Ebinezar, S Brindha, Subhashini John, Paul B Ravindran. *Dept of Radiotherapy, Christian Medical College, Vellore - 632 004, India*

**Introduction:** Multileaf collimators (MLC) are used to shape the beam for irregular shaped tumors which challenge the conventional techniques of radiotherapy and these are only available with linear accelerators. However the Isocentric $Co^{60}$ units are relatively inexpensive and found in most radiotherapy centers. Initially manual MLC was developed and later on it was motorized to accomplish the easy movement of the leaves. Now the motorized MLC is computerized in order to facilitate easy control of leaf movement. In this paper, the design and development of computer controlled MLC is discussed.

**Materials and Methods:** MLC with 15 sets of leaves is developed using low melting alloy of height 9 cm with tongue and groove arrangement to reduce interleaf leakage. These leaves are made to slide in a carriage made up of duralumin and the leaves are coupled to 30 planetary geared Swiss motors through lead screws. The coupling is made with flexible joint to get smooth transmission and is made up of brass. This MLC is attached with collimator mount made up of duralumin and is fixed to the conventional collimator of the telecobalt machine. To enable the movement of MLC leaves, a control circuit is developed which is connected to serial port of a personal computer. It consists of a serial port buffer MAX232, 1 PIC 16F88 microcontroller, 3 PIC 16F777 microcontrollers and 30 H Bridge integrated circuits TPIC0108B. The serial port of the computer is used to communicate the leaf position data to the Microcontrollers. PIC 16F88 receives all the leaf data and transmits it to the PIC 16F777. Each PIC 16F777 Microcontroller controls 10 leaves. This coding is incorporated into the microcontroller using C-language. The leaf movement is controlled digitally by the H Bridge IC TPIC 0108B. The circuit is powered with 5 Volts for buffer, microcontrollers and feed back and 12 Volts to power up all the motors. The feedback of leaf position is obtained using the linear potentiometers which is coupled with the leaves. Using potential divider method, the change in resistance is converted into change in analog voltage which is calibrated for the linear movement of the leaf. i.e., 0.1V = 1 mm. Software is developed using Visual Basic language (VB) to read the treatment planning data in ASCII format and is stored in the MLC control computer. This enables to separate the useful data from the raw data and is displayed on the screen of the MLC control computer. When the program is executed, it sends the leaf position data to the control circuit through serial port. The control circuit receives the data and calculates the error which is the difference of current position obtained from ADC and required position obtained from control software. The microcontroller nullifies the error by making the suitable adjustments in the leaf position for all leaves simultaneously.

**Results:** The performance such as field shaping, inter-leaf and intra-leaf transmission, dose delivery of Computerized MLC has been tested. The accuracy of leaf position corresponding to the field size is found to be ±1 mm.

**Conclusion:** The motorized MLC has been successfully computerized for shaping $Co^{60}$ beam precisely. This could be used as a substitute for conventional blocks in static fields, there by eliminating the effort and cost of fabricating customized blocks, the need for storage space for blocks and other practical difficulties during the process of the block making.

This PDF is available for free download from a site hosted by Medknow Publications (www.medknow.com).

### O-58
### A standard facility for the low energy photons using fluorescent X-ray beams

Sudhir Kumar, AK Mahant. *Radiation Standards Section, Radiation Safety Systems Division, Bhabha Atomic Research Centre, Mumbai -400 085, India*

**Introduction:** Fluorescent X-rays provide a near monoenergetic

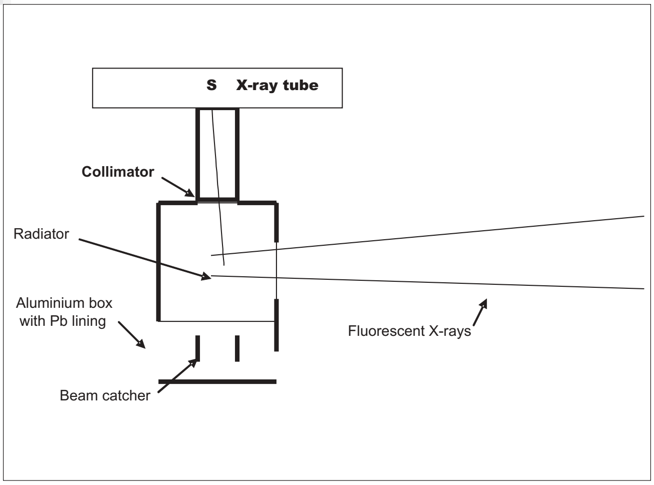

**Figure 1:** Set-up for generation of fluorescent X-rays

Table 1: Characteristics of the fluorescent X-ray beam (kVp = 80, mA = 30)

| *Radiator type* | *$HVL_1$ (mm Al)* | *$HVL_2$ (mm Al)* | *HF* | *$E_{eff}$ (keV)* | *$E_{avg}$ (keV)* | *$E_{eff}$ (keV) (Ellery et al)* | *$E_{eff}$ (keV) (Vijyamet al)* | *$K_{air}m$ Gy/min@28.7 cm* | *H' (0.07) mSv/min@28.7 cm* |
|---|---|---|---|---|---|---|---|---|---|
| Mo | 0.51 | 0.52 | 0.97 | 17.64 | 17.78 | 17.78 | 17.60 | 71.87 | 73.31 |
| Cd | 1.11 | 1.14 | 0.97 | 22.70 | 22.98 | 22.61 | 22.40 | 69.10 | 75.32 |
| Sn | 1.42 | 1.46 | 0.97 | 24.85 | 24.82 | 24.71 | 24.60 | 63.60 | 71.87 |

[Downloaded free from http://www.jmp.org.in on Friday, July 29, 2011, IP: 130.14.254.25] || Click here to download free Android application for t source of low energy photons, which can be used for the calibration of radiation monitors and detectors. Radiation measuring devices and instruments generally show a prominent energy response characteristics at low energy, hence it is important to measure their energy response in this region, specially for those detectors and radiation monitors which are specially designed to be used at low energy. Methods of generation of fluorescent X-rays has been given in the literature and experimental studies were carried out in the past.[1,2] Now a permanent set up has been made based on the earlier studies and various characteristic properties of these beams were measured to ascertain their suitability for the calibration work. This paper gives the methodology, details of the set up and the results of measurements.

**Materials and Methods:** Facility for the production of fluorescent X-rays has been established with the help of a dosimetry grade X-ray machine and three radiators viz. Cd, Mo and Sn. The potential of the X-ray machine is continuously variable from 15-320kV and the current is also adjustable from 0-30mA. Fluorescence X-rays are obtained by irradiating a radiator in the X-ray beam. The experimental set up is shown in the Figure 1. A 20 cm x 20 cm x 20 cm aluminium box having inner surfaces lined with 3 mm lead sheets has the arrangement to mount the radiators in the X-ray beams at an angle of 45°. The beam catcher is essentially a lead cylinder having 3 mm wall thickness used to minimise the contribution of scattered X-rays. Primary X-ray beam enters the box through a collimator assembly and the fluorescent X-rays are emitted in a direction perpendicular to it and pass through a collimating aperture. A 30 cc spherical reference ionisation chamber ($A_4$, Exradin, USA) was used to measure the output of the fluorescent X-rays in term of $K_{air}$ and directional dose equivalent H' (0.07). The beam homogeneity factor was determined through measurement of first and second HVL. The first HVL was also used to evaluate the effective energy and the beam uniformity was ascertained with the help of radiographic film. Spectrum measurements were carried out using a spectrometer system with Cadmium Telluride detector.

**Results:** Table 1 gives the measured value of various characteristic parameters of the fluorescent beams obtained with the three types of radiators. The Table also gives the typical measured value of $K_{air}$ and H' (0.07). The effective energy value measured by two other workers is also given for the comparison purpose. Beams were found to be uniform within 0.5% in a region of 5 cm radius around the central axis at 15 cm from the surface of the aluminium box. This ensures practically uniform radiation field over the dimensions of commonly used detectors.

**Conclusion:** The results of measurements show that the fluorescent beams fulfil all the requirements viz. Homogeneity, uniformity, effective energy and the radiation field intensity for the calibration of radiation monitors and detectors in the low energy region.

### References

1. Storm E, Lier DW, Israel HI. Photon sources for instrument calibration. Health Phys 1974;26:179-89.
2. Vijayam M, Shigwan JB, Dixit BS, Shah VV. Radiation protection and environment. 2001;24:716-21.

**O-59**

**High energy radiation dosimetry using α-$Al_2O_3$: C OSL phosphor**

DR Mishra, MS Kulkarni, NS Rawat, KP Muthe*, SD Sharma**, SK Gupta*. *Radiation Safety Systems Division, *Technical Physics and Prototype Engineering Division, **Radiological Physics and Advisory Division, Bhabha Atomic Research Centre, Mumbai - 400 085, India*

**Introduction:** Worldwide the conventional thermoluminescence (TL) approach towards radiation dosimetry is being gradually replaced by α-$Al_2O_3$:C based optically stimulated luminescence (OSL) dosimetry.[1] The OSL technique has found wide range of applications in the field of medical dosimetry for the *in-vivo* and *in-situ* dose measurements. We at BARC have recently developed an indigenous method for the synthesis of α-$Al_2O_3$:C phosphor[2] and related OSL readers for dose measurements for their applications in radiation dosimetry. The photon energy response (< 1.25 MeV) of this developed material is found to be linear up to 1Gy. The implications of these developments for high-energy medical dosimetry are discussed in the paper.

**Materials and Methods:** The α-$Al_2O_3$:C OSL grade material is synthesized using post growth Thermal Impurification Technique (PGTI) on crystalline $Al_2O_3$ plates in reducing environment in presence of graphite. The α-$Al_2O_3$:C material so produced is found to have excellent OSL response in a wide dynamic range of 50 mGy to 1Gy for $^{60}$Co irradiation. To find out the high energy response of the α-$Al_2O_3$:C, the samples were optically bleached for 30 minutes under high intensity blue light (> 200 mW/cm$^2$), to erase the residual OSL signal, if any. The high energy photon irradiations (6 to 15 MV) were carried out on the α-$Al_2O_3$:C samples in full scatter condition, using tissue equivalent phantom, at a depth of 5 cm using three medical Linear Accelerators (Varian Clinac 2100C and Clinac 2100 CD and Siemens Primus) for an absorbed dose of 1 Gy. The samples were also irradiated to 1.25 MeV photons using $^{60}$Co teletherapy machine (Theratron 780E) to an absorbed dose of 1Gy. The OSL measurements were done using photon-counting module in a programmable OSL reader interfaced to PC through an RS-232 serial interface. The samples were stimulated using continuous wave (CW) OSL mode with a 470 nm stimulation wavelength (Δλ≈20 nm, light intensity > 200 mW/cm$^2$).

**Result and Discussions:** Relative response of α-$Al_2O_3$:C samples is shown in Table 1. Normalized OSL response of α-$Al_2O_3$:C for different photon energies is also shown in Figure 1. It can be seen from the Table 1 that the response of this material is nearly the same (within

**Table 1: Integrated and normalized OSL response of the $Al_2O_3$:C crystals to high energy photons**

| *Energy (MV)* | *Integrated OSL area/counts* | *Normalized OSL response w.r.t response for 1.25 MeV photons* |
|---|---|---|
| 1.25 MeV | 2523865 | 1 |
| 6 | 2554830 | 1.0122 |
| 10 | 2496260 | 0.9890 |
| 15 | 2623390 | 1.0394 |

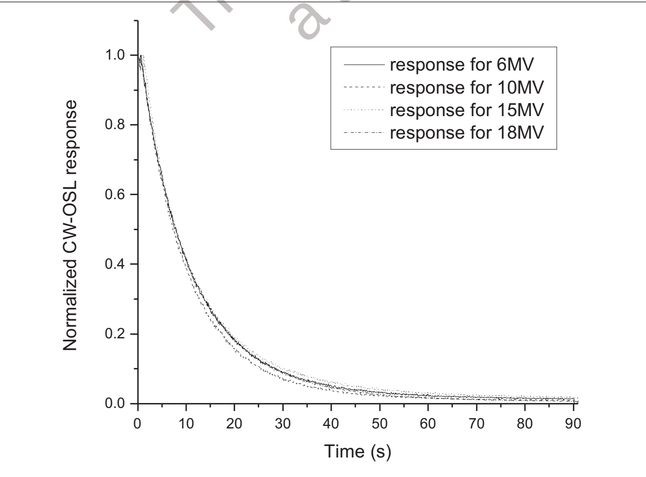

**Figure 1:** Normalized CW-OSL response of α-$Al_2O_3$:C crystals observed for various photon energies

This PDF is available for free download from a site hosted by Medknow Publications (www.medknow.com).

[Downloaded free from http://www.jmp.org.in on Friday, July 29, 2011, IP: 130.14.254.25] || Click here to download free Android application for t

±5 %) at the depth of 5 cm in the phantom. Since the size of the detectors is very small and the technique of OSL is dynamic, while making online *in-situ* measurements, $\alpha$-$Al_2O_3$:C OSL phosphor could be used for in-phantom dose measurements in medical physics applications.

**Acknowledgements:** The authors are thankful to Mrs. S. Jamema, Asst. Medical Physicist, TMH Mumbai and Mrs. Ph. Reenadevi, Medical Physicist ACTREC, Navi Mumbai for making available the Linear Accelerators.

### References

1. McKeever SW. Optically stimulated luminescence dosimetry. Nucl Instr Met Phys Res B 2001;184:29-54.
2. Kulkarni MS, Mishra DR, Muthe KP, Singh A, Roy M, Gupta SK, *et al*. An alternate method of preparation of dosimetric grade $\alpha$-$Al_2O_3$: C by vacuum-assisted post-growth thermal impurification technique. Radiat Meas 2005;39:277-82.

### O-60

### Commissioning of enhanced dynamic wedges using LDA-11

R Suresh, K Ramalingam, G Arun, M Dinesh Kumar, M Babaiah. *Yashoda Cancer Institute, Secunderabad, A.P, India*

**Introduction:** Enhanced Dynamic Wedge (EDW) an option in linear accelerators in which wedge shaped dose profile is created by the sweeping action of the Y-jaw from open to closed position while the beam is ON. Varian Linacs implement dynamic wedges using Segmented Treatment Tables (STTs) which defines monitor units as a function of the moving jaw position. For producing smaller wedge angles open field dose distribution are combined with a single 60° dynamic wedge STT known as EDW. Commissioning of EDW includes verifying the accuracy of both the isodose distributions and the monitor units (MUs) generated by the treatment planning system. The dynamic nature of nonphysical wedges requires the use of a static linear array of detectors. The aim of our work is to commission the EDW configured ECLIPSE planning system using LDA-11.

**Materials and Methods:** Our institute is equipped with Varian Clinac DMX with millennium 26 pair DMLC and EDW in which the Y jaws moves to create dynamically wedge shaped dose distribution. The EDW profiles are measured in Scanditronix RFA-300 using Linear Detector Array LDA-11 in dose integration mode. The LDA-11 has 11 detectors spaced at 2.5 cm interval. When used in dose mode the electrometer integrates the dose for a complete EDW treatment for fixed MUs. Then the detector is moved to a next position and the dose integration is repeated for same MU thus profiles were obtained with 0.5 cm resolution. For 6 MV beam, profiles for EDW angles 10°, 15°, 20°, 25°, 30°, 45° and 60° are measured at Dmax and 10 cm. For 60º EDW the profiles are measured for five different depths. The measured EDW beam profiles are compared with ECLIPSE TPS calculated dose profiles. For all wedges the effective wedge factors were measured for 6 MV photons for various symmetric field sizes using FC65G 0.6cc ionization chamber placed in SP100 water phantom at 10 cm depth with 100 cm SSD and compared with TPS values. For 10x10 $cm^2$ field percentage depth doses for a open field, 60° EDW and physical 60º wedge were measured manually using parallel plate chamber in SP100 30x30x30 $cm^3$ water phantom and compared.

**Results and Discussion:** The EDW profiles measured using LDA-11 was compared with TPS calculated dose profiles. In high dose regions the dose differences was around ±3% and in penumbra regions the DTA was about 1 to 2 mm which might due to uncertainties in measurements. The measured effective EDW factors mostly agreed within 1 to 1.5% with TPS calculated wedge factors except for 45° and 60° wedges where for field size 20x20 $cm^2$ the factor varied by 2%. The EDWF decreases with increase in field size and wedge angle. The PDD for open and EDW field were almost the same. The variation of depth dose measurements for EDW agreed within 1% with respect to depth and for physical wedge the PDD slightly increases with depth because of beam hardening. Measured profiles with LDA-11 and measured wedge factors showed good agreement with TPS values. The use of linear array chamber offered an efficient fast and reliable method for direct dynamic wedge beam profile measurements.

**Conclusion:** LDA-11 offered a fast and reliable method for commissioning EDW.

### O-61

### Investigation on the role of computed radiography as a tool for quality assurance and dosimetry in radiotherapy

Timothy Peace S, S Brindha, Paul B Ravindran. *Department of Radiotherapy, Christian Medical College, Vellore, India*

**Introduction:** To investigate the role of computed radiography with photostimulable phosphor plates (PSP) as a tool replacing conventional film for i) quality assurance and ii) dosimetry in radiotherapy. Introduction: Computed Radiography (CR) is a form of digital radiography which has been used for diagnostic imaging since 1983 and for portal imaging in radiotherapy with considerable success in recent times. Its digital nature gives several advantages over conventional films such as easier acquisition of images, economic running costs, filmless storage, quick transfer and multiple viewing of images using a Picture Archiving Communications System (PACS) network and easy analysis of images using suitable software. CR is more versatile than Electronic Portal Imaging (EPI) in that the CR plate itself is physically film-like so that it can be placed inside a phantom and irradiated from any direction.

**Materials and Methods:** Two MD40 CR cassettes (Agfa-Gevaert, Belgium) were used to acquire the images. Each cassette of size 14x17" (pixel matrix size, 2048 x 2494) consisted of a Ba.Fl.Br ($Eu^{2+}$ doped) PSP plate. For this study, the dual energy (6 and 15MV) Primus and Mevatron linear accelerators (Siemens, Germany) and the Theratron 780C telecobalt machine were used. The cassettes were read-out using the CR reader, CR75 (Agfa-Gevaert, Belgium) and the images in Digital Communication (DICOM) format were transferred through an exclusive Radiotherapy PACS Network. Quality Assurance: Quality assurance tests were performed using the PSP plates to check the field congruence, field size and field shaping of Multi-leaf Collimators (MLC). Star shots were performed to measure the isocentric shift for collimator, gantry and couch rotations. Dosimetry: The two PSP plates were individually calibrated at a source to detector distance of 150 cm. A water-filled penta step-phantom of perspex was fabricated for calibrating the PSP plates. Absolute dosimetry was performed using a calibrated 0.14 cc ion chamber (Capintec, USA). The calibration procedure involved: i) Acquisition of CR images with step-phantom positioned isocentrically for 1 and 2 MU settings. ii) Measurement of dose profile below the step-phantom with the ion chamber placed at pre-determined positions on the polystyrene slab iii) Acquisition of a CR image with step-phantom positioned isocentrically and the polystyrene slab placed on the surface of the PSP plate. This was used to locate the positions of the ion chamber with respect to the step-phantom. iv) Plotting the calibration graph for pixel values (average value within the ROI of 8 mm x 10 mm) and dose. Dosimetric tests were done using PSP plates to find out the dependence on field size, energy and dose rate, percentage depth dose curves, beam profiles and inter-leaf and intra-leaf transmission for MLCs and Micro Multi-leaf Collimators ($\mu$MLC). MATLAB and imageJ were used to analyze the images.

**Results and Discussion:** The quality assurance tests mentioned were performed and the CR images obtained were analyzed. Compared to films i) it was much easier to acquire the CR images and ii) to accurately quantify the variations. The results were found to be comparable with those of films. The calibration graphs for each of the PSP plates were plotted relating pixel values and dose. The results for the dosimetric tests were encouraging but not as satisfactory as for the QA tests. This was due the automatic post processing of the images done by the CR 75 reader and though this could be minimized it could not be completely turned off without

This PDF is available for free download from a site hosted by Medknow Publications (www.medknow.com).

[Downloaded free from http://www.jmp.org.in on Friday, July 29, 2011, IP: 130.14.254.25] || Click here to download free Android application for t disturbing the work flow of the Radiology department where the images were read out. Conclusion: Therefore it is concluded that CR using PSP plates is an excellent alternative for conventional films for quality assurance and a potential tool for dosimetry in radiotherapy.

**O-62**

**Commissioning of enhance dynamic wedge in eclipse treatment planning system**

Smriti Sharma, Shamurailatpam Dayananda Sharma, DD Deshpande. *Department of Medical Physics, Tata Memorial Hospital, Mumbai - 400 012, India*
E-mail: smriti_jp2@rediffmail.com

**Introduction:** Enhance dynamic wedge (EDW) produce wedge shape dose distribution by computer controlled movement of one of the collimator jaws under simultaneous adjustment of dose rate. The relation between delivered dose and moving jaw position is defined by a reference table called "Golden segmented treatment table (GSTT)". Eclipse treatment planning system (TPS) uses pre-existing open beam data and GSTT to generate the Enhanced Dynamic Wedge fields. Our aim is to study the characteristic of EDW on the Linear accelerator (LA) and validate the absolute dose and profiles modeled by Eclipse TPS for EDW against measured data.

**Materials and Methods:** Effective wedge factor (EWF) corresponding to EDW angles of 10, 15, 20, 25, 30, 45 and 60 degree were measured as a function of field sizes ranging from 4×4 $cm^2$ to 20×20 $cm^2$ on Clinac 6-EX LA for 6 MV X-rays using a 0.13 cc ionization chamber positioned at 5 cm depth in a 35×35×30 $cm^3$ water phantom. Depth dependence of EWF was studied for 10×10 $cm^2$ field and for all EDW angles by repeating the measurement at 10 cm depth. Off-axis profiles were measured both for physical and EDW having same standard wedge angles of 15, 30, 45 and 60 degrees using a 2D profiler kept at the isocenter of the machine. The accurate representation of EDW in Eclipse TPS was verified through absolute and relative dose measurement. Eclipse TPS calculated MUs for 200 cGy at 5 cm depth were validated for all available EDW angles using ionometric measurement on Clinac 6-EX LA. TPS generated profiles across the wedge direction was validated by comparing with the measured profile on the treatment machine. TPS calculated EWF were compared with the corresponding data measured on the linear accelerator. Results: For each EDW angle, effective wedge factor (EWF) decreases with increase in field size and found independent of the depth of measurement. The decrease in EWF per $cm^2$ ranges from 0.0066 of $10^0$ wedge to 0.0275 of $60^0$ wedge. For 10 x 10 $cm^2$ field, the EWF measured at 5 and 10 cm depth agrees within ±1% for all wedge angles. EWF calculated using standard formula available in the literature agrees with the measured value within ±1% for all wedges and up to 12x12 $cm^2$ field. This variation was found increasing up to ± 2% for field sizes up to 16x16 $cm^2$. For field size larger than 16x16 $cm^2$ variation in EWF for $60^o$ wedge angle was 3%. Off-axis profile measured for EDW and physical wedges of same angle was found to be identical except in the penumbral region corresponding to Y-stop position. TPS calculated and measured EWF agrees within ±1% for all wedge angle and field sizes. The MUs generated by Eclipse TPS are also within ±1.5% of those calculated independently. Off-axis profile generated from the TPS for EDW and physical wedge of same angle was comparable. The mean variation between measured and TPS calculated dose at 5 cm depth with EDW of all available angle was found to be ± 3.0% (SD 3.5%).

**Conclusion:** EDW provides same dose profile as from physical wedge of identical angle. EDW have the advantages over the physical wedges because of technician convenience, option of multiple angles with 5° increment. The implementation of EDW in TPS provides an additional effective tool for conformal radiotherapy treatment planning. Furthermore, it improves efficiency by removing the need to mount and un-mount physical devices. Modeling of enhance dynamic wedges in the Eclipse TPS is satisfactory for clinical use.

**O-63**

**Commissioning of motorized wedge of first telecobalt theratron Equinox-80 in eclipse 3D treatment planning system**

RA Kinhikar, S Sharma, R Upreti, CM Tambe, DD Deshpande, SK Shrivastava*, KA Dinshaw*. *Department of Medical Physics, *Radiation Oncology, Tata Memorial Hospital, Parel, Mumbai - 400 012, India*

**Introduction:** Typically, a given telecobalt machine would have a set of wedges with different field size and different angle that would be attached to the telecobalt head. A number of developments in collimator head design has brought the concept of the "universal and motorized wedge." Recently a new model of the telecobalt unit, (Theratron Equinox-80, Theratronics, Canada) with a 80-cm source to axis distance (SAD), has been first installed at Tata Memorial Hospital in June 2007. This machine apart from the fully computer controlled operations employ the unique features like asymmetric jaws (both X and Y), universal wedges (UW), a motorized wedge (MW) and a couch with the carbon fiber top. The MW filter consists of a 60-degree wedge mounted in the asymmetric collimator below the lead leaves and above the tungsten trimmer bars. The leading edge of the wedge is positioned at 43 cm from the source surface. The lead leaves and the trimmer bars move independently of the MW. The MW rotates together with the collimator and is able to travel between two fixed positions: in and out. Objective: Before clinical introduction of MW, it was required to verify the performance of the treatment planning process. In our department, the Eclipse, (Version 6.5) 3 dimensional treatment planning system (3DTPS) system (Varian, USA) was used for this purpose. The main objective of this paper was to report the methodology adopted to commission the motorized wedge in Eclipse.

**Materials and Methods:** Dosimetric measurements in Equinox-80. Measurements for motorized 60 degree wedge and universal wedges (15, 30, 45 and 60 degrees) were carried out using a blue water phantom (Scanditronix Welhofer, Germany). Central axis percentage depth doses (CAPDD) and off-axis cross-plane profiles were measured. For various filed sizes (2, 3, 4, 5, 6, 8, 10, 12, 15 and 15x20 $cm^2$) for the motorized 60 degree wedge. The MW factor was measured for various filed sizes (5, 10, 15 and 15x20 $cm^2$). A 0.6 CC cylindrical ionization chamber with and PTW Unidos electrometer was used for this purpose. Commissioning and Quality Assurance (QA) of MW in Eclipse: A hybrid generic water phantom (30 x 30 x 30 $cm^2$) was used. Two beams of 10 x 10 $cm^2$ with a UW and MW each were placed at source to surface distance (SSD) of 80 cm. A dose of 2 Gy was prescribed and normalized at the 10 cm depth. The desired motorized wedge angle was obtained with optimizing the weight of the motorized beam. The calculated CAPDD and the dose profiles for motorized wedge beam were then compared with that of the UW beam for various field sizes and for standard wedge angles of 15, 30, 45 and 60 degrees. The dose was measured for a field size of 10 x 10 $cm^2$ only in a MEDTECH water phantom (30 x 30 x 30 $cm^2$) at 10 cm depth with a CC13 ion chamber (Scanditronix Wellhofer, Germany) and a NE electrometer (Nuclear Enterprises, UK).

**Results and Conclusion:** The mean variation between measured and planned dose at 10 cm depth with MW was found to be 3.3% (SD 1.3). Figures 1 and 2 show the comparison of measured and calculated CAPDD and off-axis profiles for MW and UW respectively. The MW data for Equinox-80 was successfully commissioned in Eclipse TPS. It provides the capability of modifying the isodose characteristics of the radiation beam same as the UW. Thus MW can safely be used for clinical applications. Motorized wedges have the advantages over the standard (universal) wedges because of operator convenience. Further work, using chamber array, profiler, film dosimetry and TLD are planned to validate the present ionometric data.

This PDF is available for free download from a site hosted by Medknow Publications (www.medknow.com).
